# Supplementary material for: Bipolar‐associated miR‐499‐5p controls neuroplasticity by downregulating the Cav1.2 subunit CACNB2
Source: EMBO Rep. 2022 Aug 15;23(10):e54420. doi: 10.15252/embr.202154420 (PMC9535808; doi:10.15252/embr.202154420)
Supplement: Supplementary file 1 — Appendix [file EMBR-23-e54420-s003.pdf]

Table of Contents

Appendix Figure S1. Validation of rAAV expression via GFP in rat hippocampus 2

Appendix Figure S2. miR-499-5p expression in WT and *Cacna1c*$^{+/-}$ in rat hippocampus upon rAAV injection 3

Appendix Figure S3. Correlation analyses between peripheral miR-499-5p expression and CTQ, BDI and HAMD scores 4

Appendix Figure S4. miR-499-5p expression in human female and male subjects 6

Appendix Table S1a and S1b. Clinical data of human patients (Control, BD, and MDD) 9

Appendix Table S2. Bipolar Disorder subtypes and mood states of BD patients 11

Appendix Table S3. Clinical data of human healthy controls 11

Appendix Figure S1

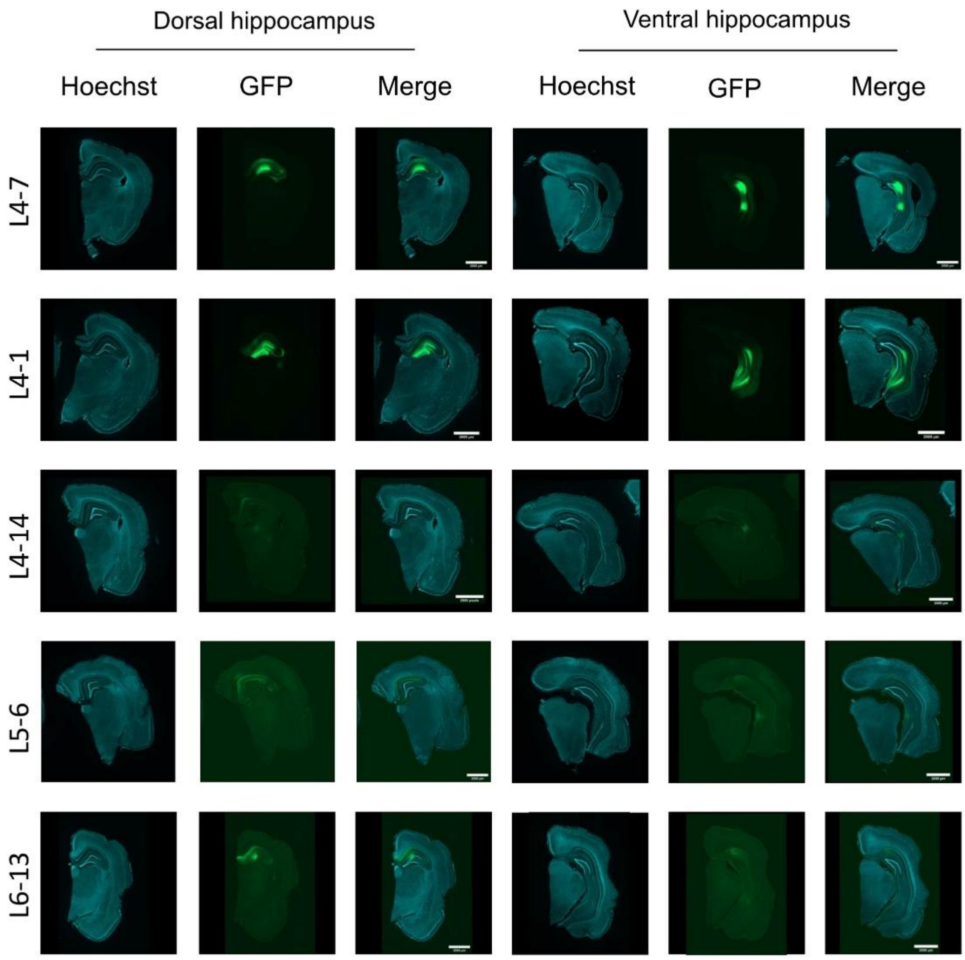

Validation of hippocampal rAAV expression by the presence of GFP fluorescence in the left hemisphere of rats expressing chimeric hairpins. Left hemispheres were dissected from rats injected bilaterally in the dorsal and ventral hippocampus with either miRNA-499 hairpin or control hairpin. Coronal brain sections of 80 µm were stained with Hoechst. Green fluorescence represents the GFP native signal of the chimeric hairpin; L4-7, L4-1, L4-14, L5-6 and L6-13: left hemisphere of rats injected with the chimeric miR-499 hairpin showing strong GFP fluorescence in both dorsal and ventral hippocampi. Scale bar: 2000 µm.

Appendix Figure S2

A-D) Fold expression of miR-499-5p in WT (A and C) and *Cacna1c*$^{+/-}$ (B and D) rats relative to control sequence expression from chimeric hairpins (blue: AAV-control; green: AAV-miR-499.Red arrows highlight animals that were excluded from behavioral analysis since miR-499-5p expression was lower than the average miR-499-5p expression of animals injected with the control hairpin + 2 S.D.

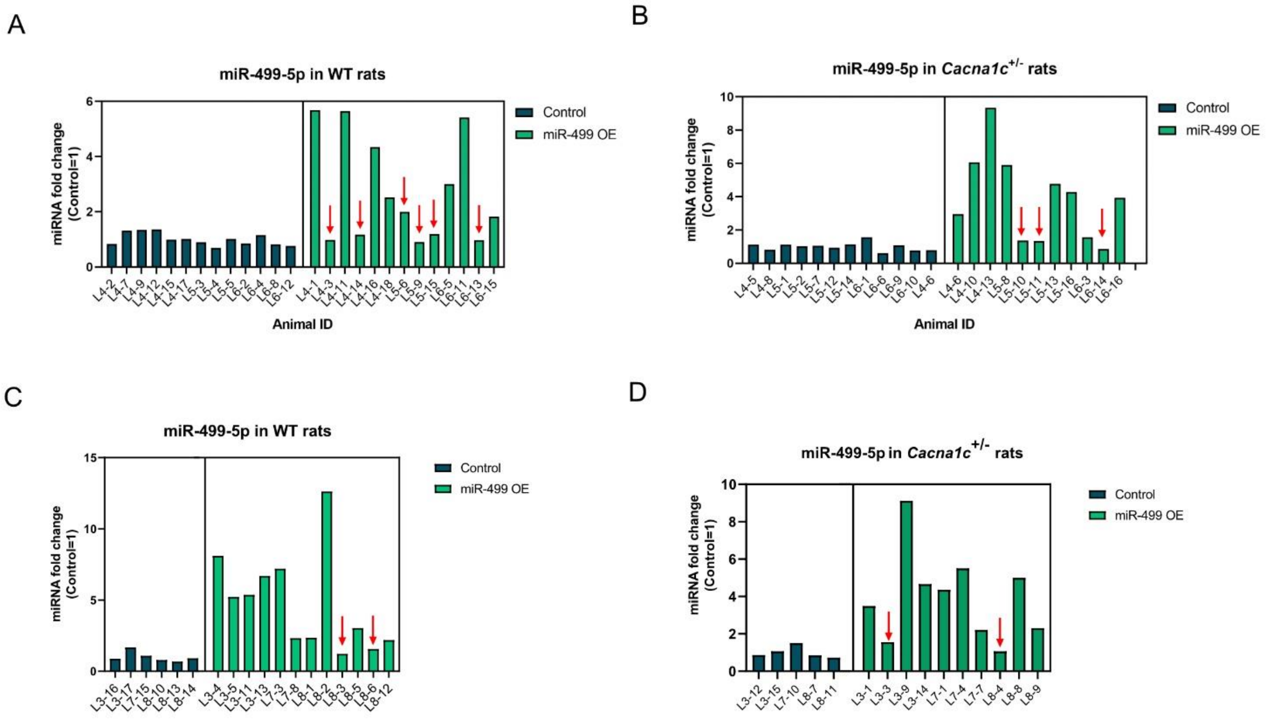

Appendix Figure S3

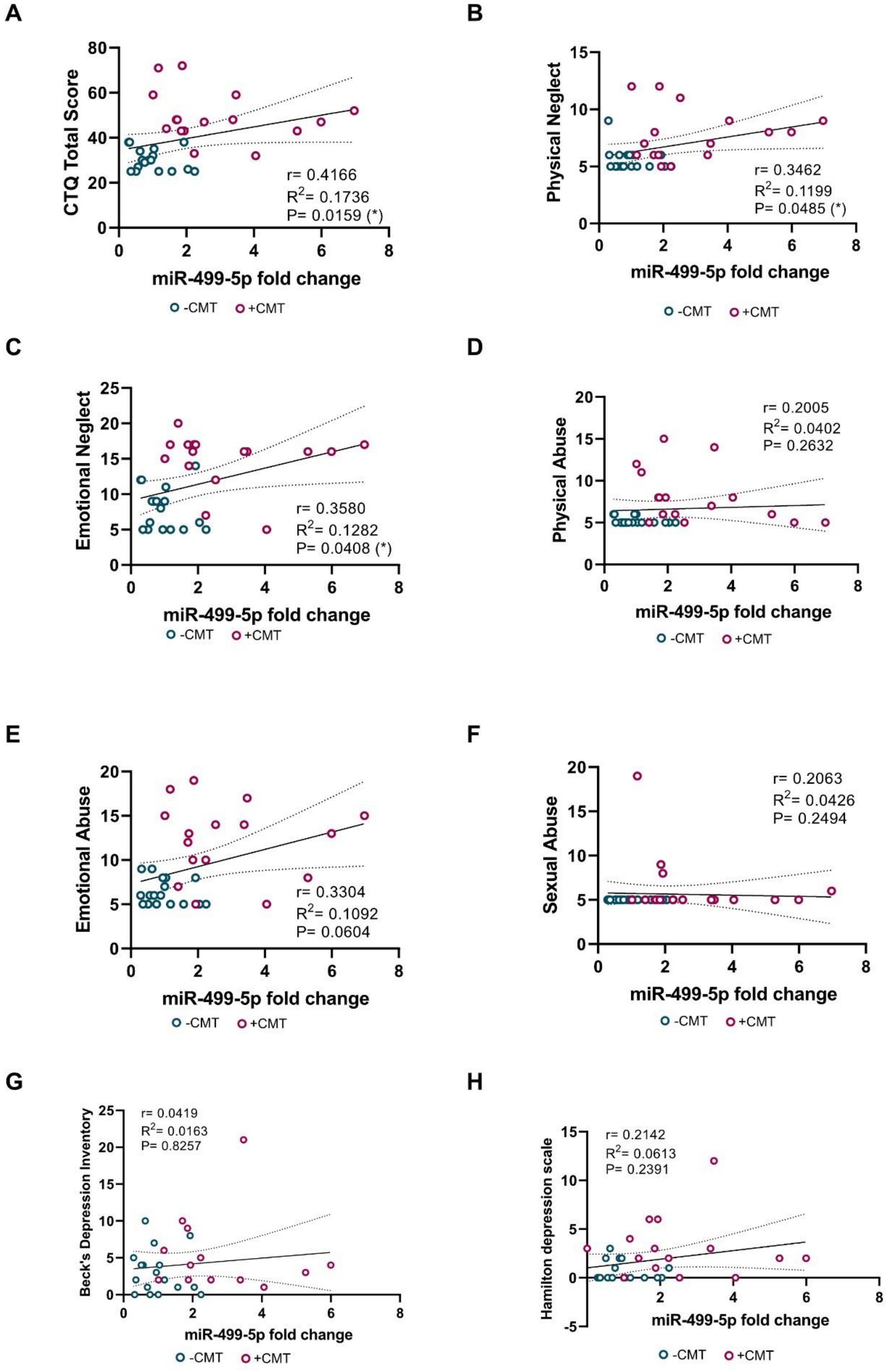

A-F) Positive correlation between the peripheral levels of miR-499-5p in control (-CMT) (blue) or maltreated (+CMT) (red) healthy individuals and A) the sum of all subscale CTQ scores (CTQ Total Score), B) the scores for physical neglect, and C) the scores for emotional neglect. No significant correlation was found between the peripheral levels of miR-499-5p and the scores for D) physical abuse, E) emotional abuse and F) sexual abuse. Spearman correlation coefficient with two-tailed analysis. Data are presented as XY tables.

G-H) No correlation between the peripheral levels of miR-499-5p in control (-CMT) (blue) or maltreated (+CMT) (red) healthy individuals and G) Becks's depression Inventory (BDI) and H) Hamilton depression scale (HAMD) scores. Spearman correlation coefficient with two-tailed analysis. Data are presented as XY tables.

Appendix Figure S4

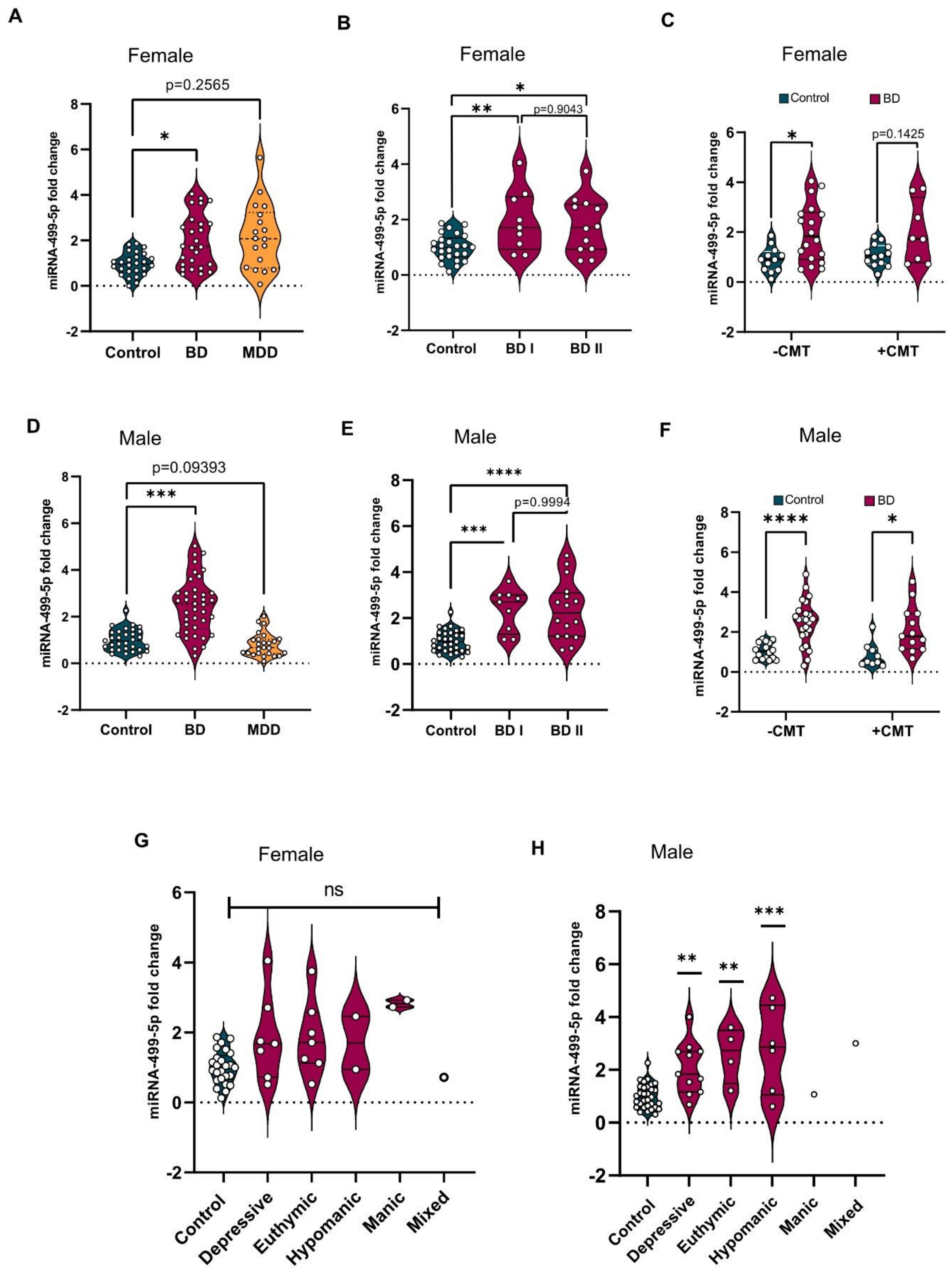

A) miR-499-5p qPCR analysis of total RNA isolated from PBMCs of female subjects (control, n= 26; BD, n=26; MDD, n=18). Peripheral miR-499-5p is significantly up-regulated in BD patients (**p=0.00824, Wilcoxon rank-sum test) but not in MDD patients (p=0.11457, Wilcoxon rank-sum test) compared to control subjects after correction for age and antidepressant treatment (linear model of the form FC ~ Group + Age + Antidepressant treatment). Data are presented as violin plots with median, quartiles and data points.

B) miR-499-5p qPCR analysis of total RNA isolated from PBMCs of female subjects (control, n=26; BD I, n=8; BD II, n=12). One-way ANOVA, Post hoc Tukey test: Control vs BD I: **p=0.0099, Control vs BD II: *p=0.0170, BD I and BD II p=0.9043). Data are presented as violin plots with median, quartiles and data points.

C) miR-499-5p qPCR analysis of total RNA isolated from PBMCs of female subjects (control -CMT: n=10; control +CMT: n= 11; BD -CMT: n=18; BD +CMT: n=8). Two-way ANOVA, Post-hoc Tukey Test. Main effect CMT: p= 0.74151, main effect Group: ***p=0.0006. Control -CMT vs BD -CMT: *p=0.0146; Control +CMT vs BD +CMT: ns, p=0.1425. Data are presented as violin plots with median, quartiles and data points.

D) miR-499-5p qPCR analysis of total RNA isolated from PBMCs male subjects (control, n= 31; BD, n=37; MDD, n=24). Peripheral miR-499-5p is significantly up-regulated in BD patients (****p= 4.38e-07, Wilcoxon rank-sum test) but not in MDD patients (p= 0.09393, Wilcoxon rank-sum test) compared to control subjects after correction for age and antidepressant treatment (linear model of the form delta FC~ Group + Age + Antidepressant treatment). Data are presented as violin plots with median, quartiles and data points.

E) miR-499-5p qPCR analysis of total RNA isolated from PBMCs of male subjects (control, n=31; BDI, n= 9; BD II, n=16). One-way ANOVA, Post hoc Tukey test: Control vs BD I: ***p=0.0003, Control vs BD II: ****p=0.00001, BD I and BD II p=0.9994). Data are presented as violin plots with median, quartiles and data points.

F) miR-499-5p qPCR analysis of total RNA isolated from PBMCs of male subjects (control -CMT: n=16; control +CMT: n= 9; BD -CMT: n=24; BD +CMT: n=13). Two-way ANOVA, Post-hoc Tukey Test. Main effect CMT: p= 0.2593, main effect Group: ****p= 4.01e-07, interaction CMT x Group: ns, p=0.790. Control -CMT vs BD -CMT: ****p=0.00010; Control +CMT vs BD +CMT: *, p= 0.0120. Data are presented as violin plots with median, quartiles and data points.

G) miR-499-5p qPCR analysis of total RNA isolated from PBMCs of female subjects (control, n= 26) and BD subjects in different mood states (depressive, n=7; euthymic, n= 7; hypomanic, n=2; manic, n=2; mixed, n=1). One-way ANOVA, Post hoc Tukey test. Data are presented as violin plots with median, quartiles and data points.

H) miR-499-5p qPCR analysis of total RNA isolated from PBMCs of male subjects (control, n= 31) and BD subjects in different mood states (depressive, n=11; euthymic, n= 4; hypomanic, n=6; manic, n=1; mixed, n=1). One-way ANOVA, Post hoc Tukey test: Control vs. Depressive, **p=0.0071; Control vs. Euthymic, **p=0.0098; Control vs. Hypomanic, ***p=0.0002. Data are presented as violin plots with median, quartiles and data points.

Appendix Table S1A and S1B

Subjects used for miR-499-5p expression analysis on psychiatrically healthy controls (Control), Bipolar disorder patients (BD) or Major Depressive Disorder patients (MDD). One-way-ANOVA was performed to evaluate significant differences between groups. S.D.: standard deviation, CTQ: Childhood Maltreatment Questionnaire, HAMD: Hamilton Depression Rating Scale, YMRS: Young Mania Rating Scale, BDI: Beck's Depression Inventory, AD: antidepressant use.

**Table S1A**	**Control**	**BD**	**MDD**	**P value**
**n**	26	26	18	N/A
**Sex**	Female	Female	Female	N/A
**Age ± S.D.**	29.4 ± 5.4	29.9 ± 5.4	29.6± 4.9	0.94
**CTQ ± S.D. (%)**	40.7 ± 11.4 (53.8%)	42.3 ± 13.2 (30.8%)	50.2 ± 20.2 (61%)	0.1067
**Family history of Affective Disorders (%)**	8 (30.8%)	10 (38.5%)	5 (27.8%)	N/A
**HAMD ±S.D.**	2.7 ± 3.9	7.5 ± 6.2	14.8 ± 6.7	<0.0001
**YMRS ± S.D.**	0.5 ± 1.1	2.4 ± 2.9	1.4 ± 1.6	<0.01
**BDI ± S.D.**	6.1 ± 6.1	12.9 ± 11.1	26.6 ± 11.0	<0.0001
**AD (%)**	0 (0%)	7 (26.9%)	16 (88.9%)	N/A
**Antipsychotic (%)**	0 (0%)	12 (46.15%)	7 (38.89%)	N/A
**Lithium (%)**	0 (0%)	4 (15.38%)	1 (5.56%)	N/A
**Anticonvulsive (%)**	0 (0%)	6 (23.08%)	1 (5.56%)	N/A
**Stimulants (%)**	0 (0%)	0 (0%)	0 (0%)	N/A
**Benzodiazepine (%)**	0 (0%)	0 (0%)	1 (5.56%)	N/A
**Z substance (%)**	0 (0%)	0 (0%)	1 (5.56%)	N/A

Table S1B	Control	BD	MDD	P value
n	26	26	18	N/A
**Sex**	Female	Female	Female	N/A
**Age ± S.D.**	29.4 ± 5.4	29.9 ± 5.4	29.6± 4.9	0.94
**CTQ ± S.D. (%)**	40.7 ± 11.4 (53.8%)	42.3 ± 13.2 (30.8%)	50.2 ± 20.2 (61%)	0.1067
**Family history of Affective Disorders (%)**	8 (30.8%)	10 (38.5%)	5 (27.8%)	N/A
**HAMD ±S.D.**	2.7 ± 3.9	7.5 ± 6.2	14.8 ± 6.7	<0.0001
**YMRS ± S.D.**	0.5 ± 1.1	2.4 ± 2.9	1.4 ± 1.6	<0.01
**BDI ± S.D.**	6.1 ± 6.1	12.9 ± 11.1	26.6 ± 11.0	<0.0001
**AD (%)**	0 (0%)	7 (26.9%)	16 (88.9%)	N/A
**Antipsychotic (%)**	0 (0%)	12 (46.15%)	7 (38.89%)	N/A
**Lithium (%)**	0 (0%)	4 (15.38%)	1 (5.56%)	N/A
**Anticonvulsive (%)**	0 (0%)	6 (23.08%)	1 (5.56%)	N/A
**Stimulants (%)**	0 (0%)	0 (0%)	0 (0%)	N/A
**Benzodiazepine (%)**	0 (0%)	0 (0%)	1 (5.56%)	N/A
**Z substance (%)**	0 (0%)	0 (0%)	1 (5.56%)	N/A

Appendix Table S2

BD subtype (I/II/NA) and Mood state (d: depressive, e: euthymic, h: hypomanic, m: manic; mi: mixed) of the BD subjects used for miR-499-5p expression analysis; NA: information not available.

**Table S2**	**Female subjects**	**Male subjects**
**BD subtype (I/II/NA)**	9/12/5	11/16/10
**Mood state (d/e/h/m/mi/NA)**	7/7/2/2/1/7	11/4/6/1/1/14

Appendix Table S3

Subjects used for miR-499-5p expression analysis in healthy controls (Control) and subjects with a history of childhood maltreatment (Maltreated). Unpaired t-Test was performed to evaluate significant differences between groups. S.D.: standard deviation, CTQ: Childhood Maltreatment Questionnaire, AD: antidepressant use.

**Table S3**	**Control**	**Maltreated**	**P value**
n	18	17	N/A
**Sex**	Female	Female	N/A
**Age ± S.D.**	27.4 ± 7.2	32.3 ± 10.0	0.11
**CTQ ± S.D. (%)**	29.8 ± 4.9 (0%)	49.3 ± 10.9 (100%)	<0.0001
