# Supplementary material for: Bipolar‐associated miR‐499‐5p controls neuroplasticity by downregulating the Cav1.2 subunit CACNB2
Source: EMBO Rep. 2022 Aug 15;23(10):e54420. doi: 10.15252/embr.202154420 (PMC9535808; doi:10.15252/embr.202154420)
Supplement: Supplementary file 2 — Expanded View Figures PDF [file EMBR-23-e54420-s001.pdf]

Expanded View Figures

Figure EV1. Gene expression analysis in the JSI rat model and primary rat hippocampal neurons.

A Schematic representation of juvenile social isolation (JSI) experiment.

B–E qPCR analysis for c-fos (B), arc (C), miR-146b (D), and miR-30e-5p (E) using total RNA isolated from the hippocampus of male rats that were either group-housed or socially isolated for 4 weeks postweaning (n = 9 rats per group). Data are represented as box plot with whiskers (+: mean, line: median; whiskers: Tukey) (*P = 0.0207 (c-fos), *P = 0.0207 (arc); ***P = 0.0006 (miR-146-5p); P = 0.1049 (miR-30e-5p); Mann–Whitney U-test). Fold changes represent changes in gene expression relative to the control condition. U6 snRNA was used for normalization. ns = not significant.

F Relative expression of miR-499-5p in primary hippocampal neurons at different *DIVs* (n = 3 independent experiments). Total RNA was obtained from nontreated developing hippocampal neurons or treated from DIV 3 with FUDR to stop the proliferation of nonneuronal glial cells at the six indicated time points. Fold changes represent changes in miR-499-5p expression relative to DIV 4. Data are represented on XY graph as mean ± SD.

G Relative expression of miR-499-5p is significantly induced in hippocampal neurons treated with DEX compared to DMSO-treated neurons (n = 4; Paired two-sample t-test, *P = 0.0462). Data are represented as scattered dot plots with bar, mean ± SD. Fold change represents changes in miR-499-5p expression of DEX-treated neurons relative to DMSO-treated neurons.

H Relative expression of miR-499-5p is significantly induced in hippocampal neurons transfected with miR-499-5p mimics (n = 3 independent experiments; Ratio paired t-test, ***P = 0.003). Data are represented as scattered dot plots with bar, mean ± SD. Fold change represents changes in miR-499-5p expression of miR-499-5p mimic transfected-neurons relative to control mimic-transfected-neurons.

I Mean of the Sholl profile averages from biological replicates of Fig 1D and E. Data are represented on XY graph as mean ± SD. *$P < 0.05$.

© 2022 The Authors Published under the terms of the CC BY 4.0 license

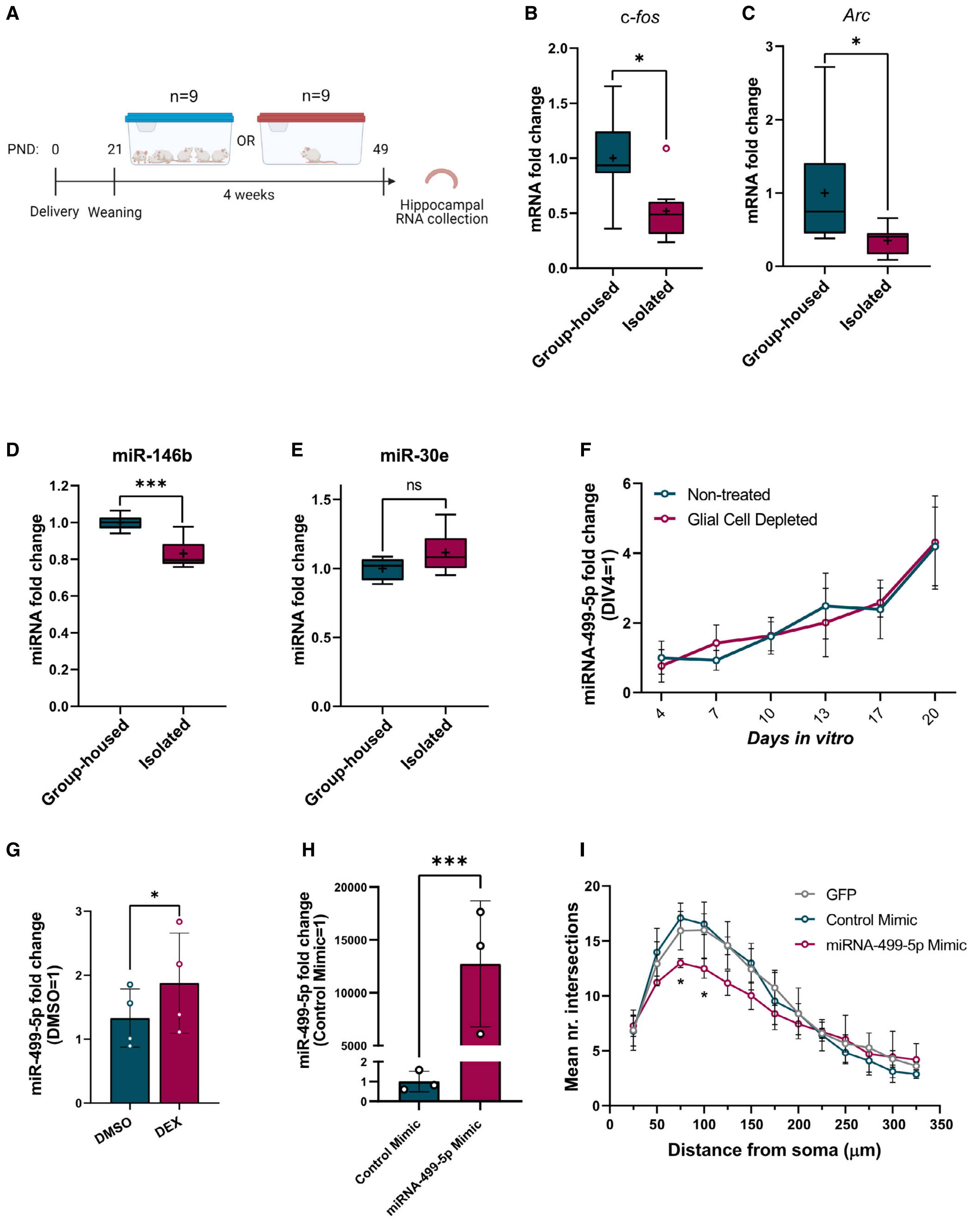

Figure EV1.

 © 2022 The Authors Published under the terms of the CC BY 4.0 license

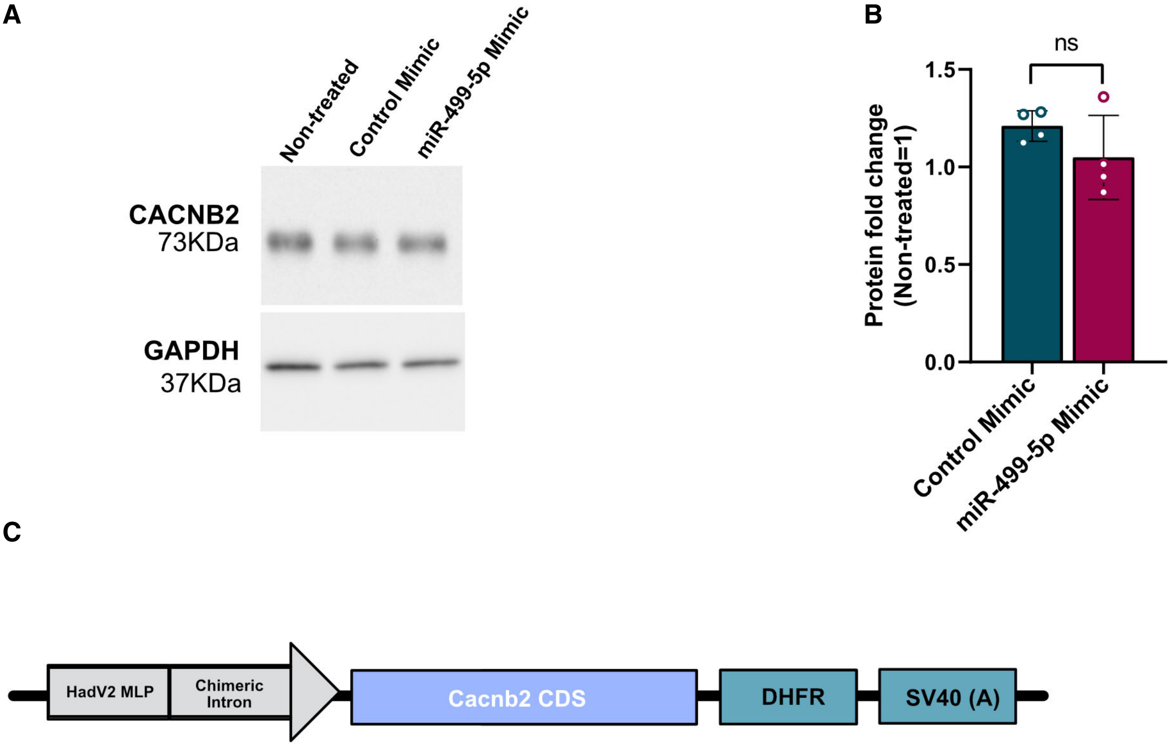

Figure EV2. CACNB2 protein expression upon miR-499-5p overexpression in rat hippocampal neurons.

A Representative western blot image of CACNB2 (upper panel) and GAPDH (lower panel) protein expression levels in hippocampal neurons (DIV14) that were transfected with the miR-499-5p mimic or with the control mimic at DIV 7. GAPDH was used as a loading control.

B Western blot analysis showed that miR-499-5p overexpression did not significantly change the CACNB2 expression compared to neurons expressing the control mimic (n = 4 independent experiments; Unpaired two-sample t-test, P = 0.2094). Data are represented as scattered dot plots with bar, mean ± SD.

C Schematic illustration of the pMT2-Cacnb2 overexpressing construct (hadV2 MLP: HadV2 Major Late Promoter; Cacnb2 CDS: Cacnb2 Coding Sequence; DHFR: mouse Dihydrofolate reductase).

Figure EV3. Quantification of Ca$_v$1.2 cell surface expression and channel properties upon miR-499-5p overexpression in hippocampal neurons.

A, B Quantification of the total levels of Cav1.2 channels of live-stained neurons transfected as in 3A. Neurons transfected with miR-499-5p mimic showed significant reductions in (A) the integrated density of surface Cav1.2-HA (n = 4; Paired two-sample t-test *P = 0.0385) and (B) the area of surface Cav1.2-HA (n = 4 independent experiments; Paired two-sample t-test *P = 0.0381). Data are represented as scattered dot plots with bar, mean ± SD.

C Representative images of *DIV* 19 rat hippocampal neurons co-transfected with GFP (green channel) and Cav1.2-HA (red channel), together with either control or miR-499-5p mimics. After 12–13 days of expression, labeling with Anti-HA antibodies was performed under permeabilized conditions to identify total levels of Cav1.2 channels. Scale bars = 20 μm.

D–F Quantification of the total levels of Cav1.2 channels of permeabilized neurons transfected as in Fig EV3C. No significant changes were found for (D) the total levels of Cav1.2 (n = 4 independent experiments; Paired two-sample t-test, P = 0.9776), (E) the integrated density (n = 4 independent experiments; Paired two-sample t-test, P = 0.3213), (F) the area (n = 4 independent experiments; Paired two-sample t-test, P = 0.3182) of total Cav1.2 channels. Data are represented as scattered dot plots with bar, mean ± SD.

G I/V curves before and after bath application with the LVGCC blocker Nifedipine (20 μM) (n = 3 cells nontreated vs. n = 3 cells treated with 20 μM Nifedipine). Data are represented on XY graph as mean ± SEM.

H Hippocampal neurons transfected with the miR-499-5p mimic did not show a different activation curve compared to the activation curves of cells transfected with the Control mimic or GFP alone (GFP: n = 9; NC mimic: n = 10, miR-499-5p mimic: n = 10). Data are represented on XY graph as mean ± SEM.

I Hippocampal neurons overexpressing miR-499-5p did not show a different inactivation curve compared to the inactivation curves of control cells (GFP: n = 9; NC mimic: n = 10, miR-499-5p mimic: n = 10). Data are represented on XY graph as mean ± SEM.

J MiR-499-5p overexpression tended to decrease capacitance (GFP: n = 9; NC mimic: n = 10, miR-499-5p mimic: n = 10; Unpaired two-sample t-test, P = 0.0698). Data are represented as box plot with whiskers and data points (line: median; whiskers: minimum and maximum values).

© 2022 The Authors Published under the terms of the CC BY 4.0 license

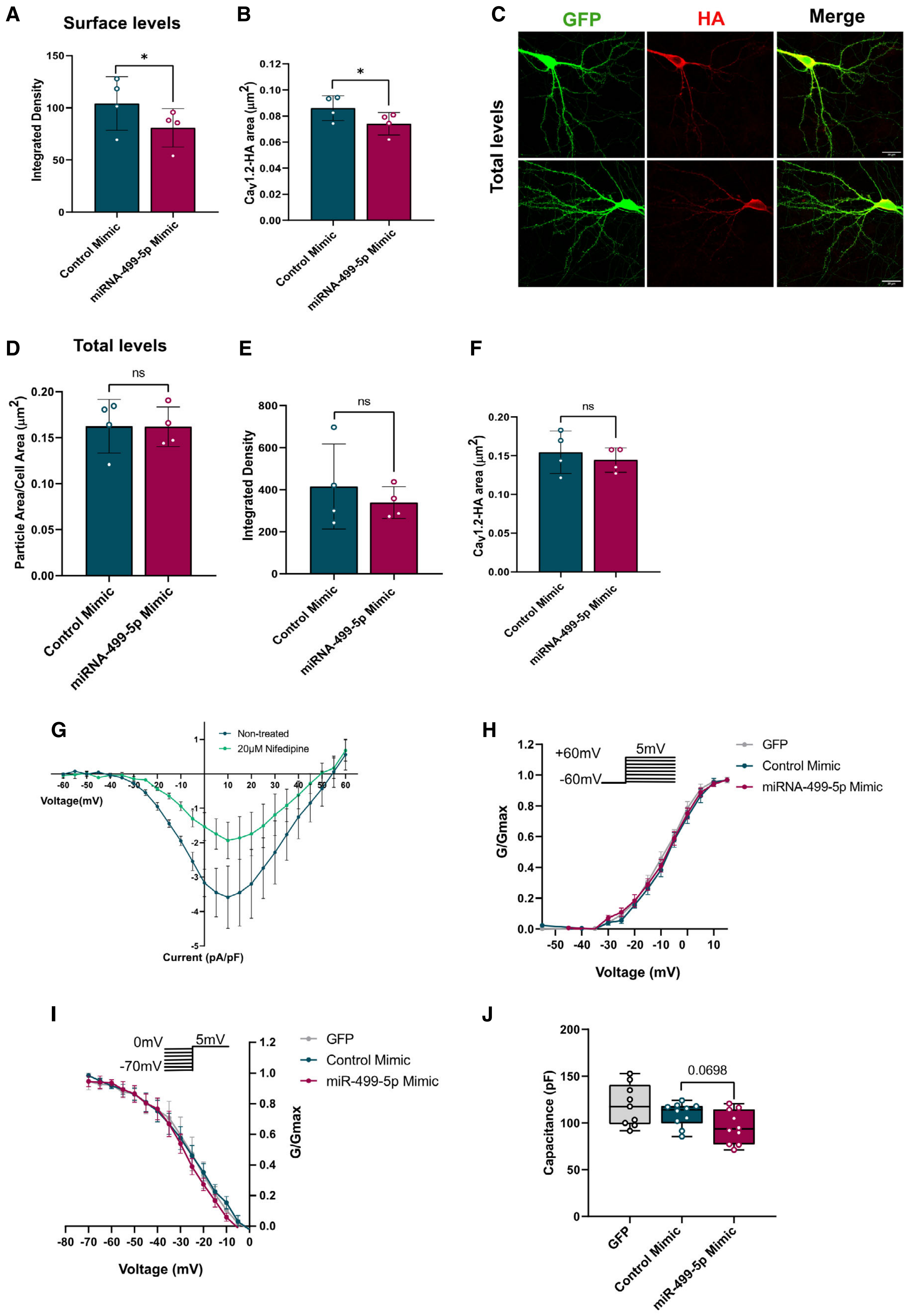

Figure EV3.

 © 2022 The Authors Published under the terms of the CC BY 4.0 license

Figure EV4. Additional biochemical and behavioral characterization of WT and Cacna1c$^{+/-}$ rats overexpressing miR-499-5p.

A, B Significant negative correlation between relative miR-499-5p expression and *CACNB2* protein levels in the hippocampus of WT (A) and Cacna1c$^{+/-}$ rats (B) injected with AAV-Control (red) and AAV-miR-499 (blue). Spearman correlation coefficient with two-tailed analysis is provided in the legend.

C Object acquisition task. Total time (seconds) WT or *Cacna1c*$^{+/-}$ rats injected with the indicated AAV spent exploring the objects. Data are presented as box and whisker plot with median, mean, and minimum and maximum values. ns: not significant, one-way ANOVA; WT_Control: $n = 18$ (eight males, 10 females); WT_miR-499: $n = 16$ (seven males, nine females); *Cacna1c*$^{+/-}$_Control: $n = 15$ (five males, 10 females); *Cacna1c*$^{+/-}$_miR-499: $n = 16$ (10 males, six females). Main effect Genotype: $F_{1, 61} = 0.501$, $P = 0.482$; main effect Treatment: $F_{1, 61} = 0.929$, $P = 0.339$; Interaction Genotype × Treatment: $F_{1, 61} = 0.098$, $P = 0.756$.

D Total arms entries during Elevated Plus Maze test did not differ between genotypes and treatment groups (as in Fig 4H). Data are presented as box and whisker plot with median, mean, and minimum and maximum values. Ns: not significant, one-way ANOVA. Main effect Genotype: $F_{1, 64} = 0.257$, $P = 0.614$, main effect Treatment: $F_{1, 64} = 0.007$, $P = 0.934$, interaction Genotype × Treatment: $F_{1, 64} = 0.005$, $P = 0.946$.

E Open field test. Total distance traveled (cm) by WT or *Cacna1c*$^{+/-}$ rats injected with the indicated AAV over a time course of 10 min on 2 consecutive days. Data are presented as means ± SD. Repeated measures ANOVA; WT_Control: $n = 19$ (nine males, 10 females); WT_miR-499: $n = 16$ (seven males, nine females); *Cacna1c*$^{+/-}$_Control: $n = 17$ (seven males, 10 females); *Cacna1c*$^{+/-}$_miR-499: $n = 16$ (10 males, six females). Main effect Genotype: $P = 0.138$; main effect Treatment: $P = 0.672$; interaction Genotype × Treatment: $P = 0.124$.

© 2022 The Authors Published under the terms of the CC BY 4.0 license

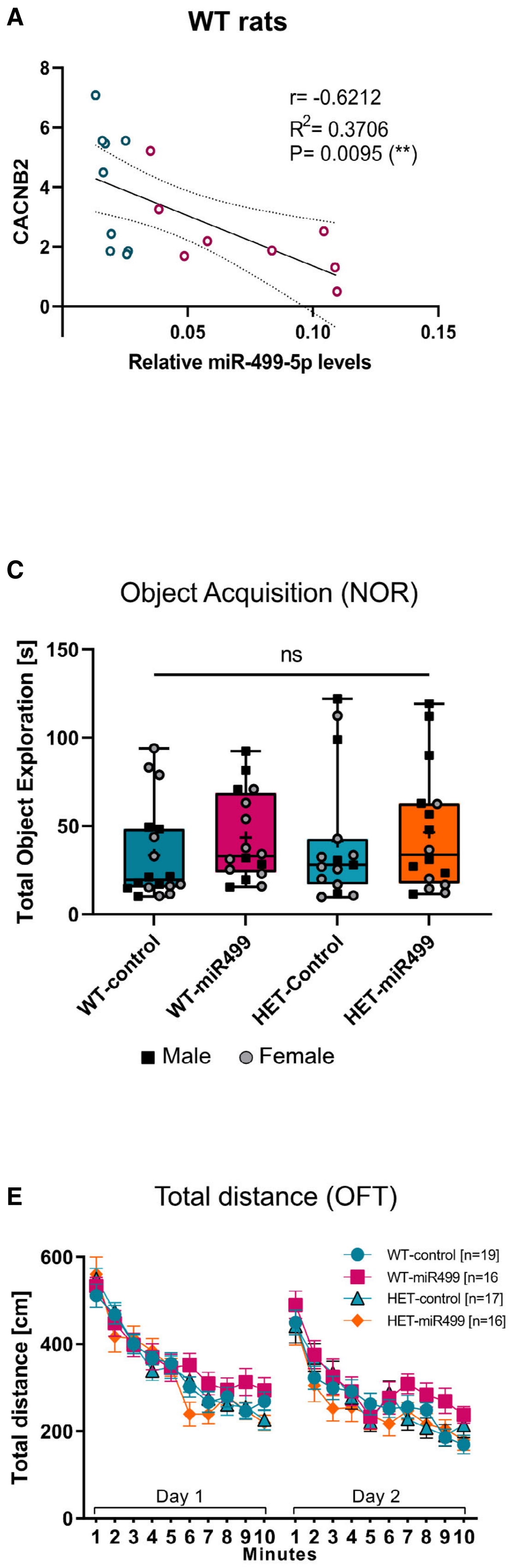

Figure EV4.

© 2022 The Authors Published under the terms of the CC BY 4.0 license

Figure EV5. Additional analysis of miR-499-5p expression in different healthy control and patient subgroups.

A Schematic illustration of the experimental workflow. Total RNA was isolated from PBMCs of psychiatrically healthy subjects (Controls or maltreated), BD and MDD patients for miRNA expression analysis.

B miR-499-5p qPCR analysis of total RNA isolated from PBMCs of control (female = 26, male = 31), BD subjects (female = 19, male = 21), or BD subjects under Antidepressant (AD) treatment (female = 7, male = 16) subjects. Two-way ANOVA, Main effect Sex: $P = 0.378$; main effect Group: ****$P = 1.68e-11$; Interaction group × sex: $P = 0.663$. Tukey's HSD: control versus BD and control versus BD + AD: ****$P < 0.00001$; BD versus BD + AD *$P = 0.02526$. Data are presented as violin plots with median, quartiles and data points.

C miR-499-5p qPCR analysis of total RNA isolated from PBMCs of control (female = 26, male = 31), MDD subjects (female = 1, male = 9), or MDD subjects under Antidepressant (AD) treatment (female = 15, male = 16) subjects. Two-way ANOVA, Main effect Sex: **$P = 0.003247$; main effect Group: *$P = 0.010018$; Interaction group × sex: ***$P = 0.000297$. Tukey's HSD: control versus MDD: $P = 0.5190$; control versus MDD + AD: *$P = 0.0306$; MDD versus MDD + AD: *$P = 0.0274$. Data are presented as violin plots with median, quartiles, and data points.

D miR-499-5p qPCR analysis of total RNA isolated from PBMCs of control −CMT (female = 10, male = 16), control +CMT (female = 11, male = 9), BD −CMT (female = 18, male = 24), and BD + CMT (female = 8, male = 13). Three-way ANOVA, main effect Sex: $P = 0.4991$, main effect CMT: $P = 0.5434$; main effect Group: $P < 0.0001$; interaction CMT × Group: $P = 0.7532$; interaction CMT × Sex: $P = 0.3259$; interaction Group × Sex: $P = 0.3259$; interaction CMT × Group × Sex: $P = 0.9703$. Tukey's HSD: control CMT− versus BD CMT−: ****$P < 0.0001$; control CMT+ versus BD CMT+: **$P = 0.0013$. Data are presented as violin plots with median, quartiles, and data points.

E, F miR-499-5p expression does not correlate with Young Mania Rating Scale or Beck's Depression Inventory scores from BD patients. Spearman correlation coefficient with two-tailed analysis is provided in the legend. Squares: males, circles: females.

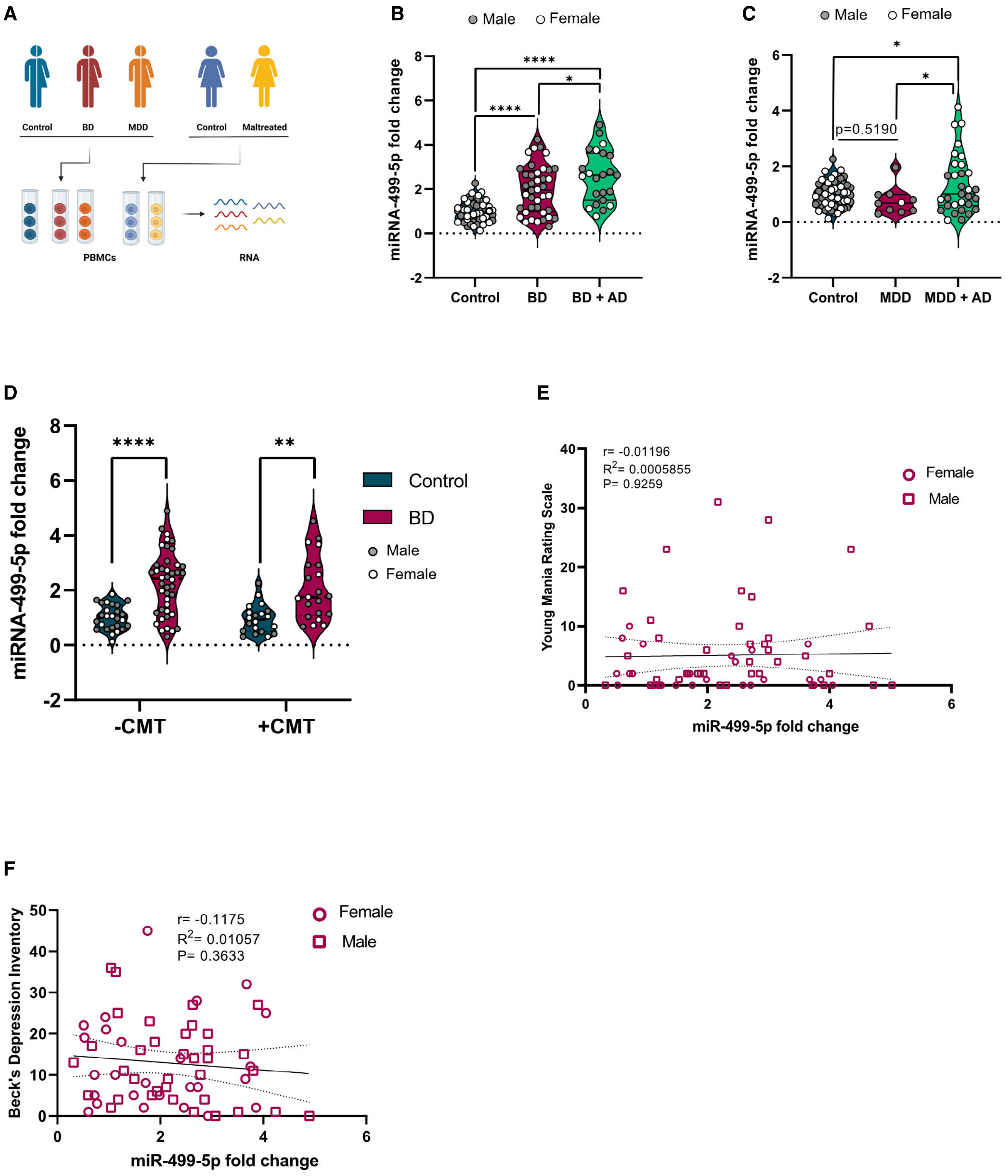

Figure EV5.

© 2022 The Authors Published under the terms of the CC BY 4.0 license
