# Supplementary material for: Bipolar‐associated miR‐499‐5p controls neuroplasticity by downregulating the Cav1.2 subunit CACNB2
Source: EMBO Rep. 2022 Aug 15;23(10):e54420. doi: 10.15252/embr.202154420 (PMC9535808; doi:10.15252/embr.202154420)
Supplement: Supplementary file 3 — PDF+ [file EMBR-23-e54420-s004.pdf]

Article

TRANSPARENT PROCESS OPEN ACCESS

EMBO reports

Bipolar-associated miR-499-5p controls neuroplasticity by downregulating the Cav1.2 subunit CACNB2

Helena C Martins[1,†], Carlotta Gilardi[1,†], A Özge Sungur[2,3], Jochen Winterer[1], Michael A Pelzl[4,9], Silvia Bicker[1], Fridolin Gross[1], Theresa M Kisko[2], Natalia Malikowska-Racia[2,10], Moria D Braun[2], Katharina Brosch[5], Igor Nenadic[5], Frederike Stein[5], Susanne Meinert[6], Rainer K W Schwarting[2,3], Udo Dannlowski[6], Tilo Kircher[5], Markus Wöhr[2,3,7,8] & Gerhard Schratt[1,*]

Abstract

Bipolar disorder (BD) is a chronic mood disorder characterized by manic and depressive episodes. Dysregulation of neuroplasticity and calcium homeostasis are frequently observed in BD patients, but the underlying molecular mechanisms are largely unknown. Here, we show that miR-499-5p regulates dendritogenesis and cognitive function by downregulating the BD risk gene CACNB2. miR-499-5p expression is increased in peripheral blood of BD patients, as well as in the hippocampus of rats which underwent juvenile social isolation. In rat hippocampal neurons, miR-499-5p impairs dendritogenesis and reduces surface expression and activity of the L-type calcium channel Cav1.2. We further identified CACNB2, which encodes a regulatory β-subunit of Cav1.2, as a direct functional target of miR-499-5p in neurons. miR-499-5p overexpression in the hippocampus *in vivo* induces short-term memory impairments selectively in rats haploinsufficient for the Cav1.2 pore forming subunit Cacna1c. In humans, miR-499-5p expression is negatively associated with gray matter volumes of the left superior temporal gyrus, a region implicated in auditory and emotional processing. We propose that stress-induced miR-499-5p overexpression contributes to dendritic impairments, deregulated calcium homeostasis, and neurocognitive dysfunction in BD.

Keywords bipolar disorder; calcium channel; cognitive function; microRNA; neuroplasticity

Subject Categories Molecular Biology of Disease; Neuroscience; RNA Biology

DOI 10.15252/embr.202154420 | Received 1 December 2021 | Revised 21 July 2022 | Accepted 26 July 2022 | Published online 15 August 2022

EMBO Reports (2022) 23: e54420

Introduction

Bipolar disorder (BD) is a severe and chronic mood disorder defined by recurring (hypo)manic and depressive episodes. It represents a highly debilitating condition leading to cognitive impairments and an especially high risk for suicide deaths (Solé *et al*, 2017; Dong *et al*, 2020). BD has one of the highest heritability rates among mental illnesses (Johansson *et al*, 2019). As a result, genetic studies found important susceptibility genes but also revealed a complex and heterogeneous genetic architecture where no single gene variation by itself is sufficient to cause the disorder. Therefore, according to the current consensus, an interaction between genetic and environmental (GxE) risk factors is required for a full manifestation of the disease in affected individuals.

Regarding the genetic component, recent GWAS and analysis of *de novo* mutations found strong associations between single nucleotide polymorphisms (SNPs) in L-type voltage-gated calcium channel (LVGCC) coding genes and BD. This includes genetic variants in the CACNA1C (Moskvina *et al*, 2009) and CACNB2 (Cross-Disorder Group of the Psychiatric Genomics Consortium *et al*, 2013) loci,

1 Lab of Systems Neuroscience, Department of Health Science and Technology, Institute for Neuroscience, Swiss Federal Institute of Technology ETH, Zurich, Switzerland
2 Behavioural Neuroscience, Experimental and Biological Psychology, Faculty of Psychology, Philipps-University of Marburg, Marburg, Germany
3 Center for Mind, Brain, and Behavior, Philipps-University of Marburg, Marburg, Germany
4 Institute for Physiological Chemistry, Biochemical-Pharmacological Center Marburg, Philipps-University of Marburg, Marburg, Germany
5 Department of Psychiatry and Psychotherapy, University of Marburg, Marburg, Germany
6 Institute for Translational Psychiatry, University of Münster, Münster, Germany
7 Social and Affective Neuroscience Research Group, Laboratory of Biological Psychology, Research Unit Brain and Cognition, Faculty of Psychology and Educational Sciences, KU Leuven, Leuven, Belgium
8 Leuven Brain Institute, KU Leuven, Leuven, Belgium
9 Present address: Psychiatry and Psychotherapy, University of Tübingen, Tübingen, Germany
10 Present address: Department of Behavioral Neuroscience and Drug Development, Maj Institute of Pharmacology, Polish Academy of Sciences, Krakow, Poland
*Corresponding author. Tel: +41 44 633 81 32; E-mail: gerhard.schratt@hest.ethz.ch
†These authors contributed equally to this work

© 2022 The Authors. Published under the terms of the CC BY 4.0 license

which encode the α_1 pore subunit and the auxiliary β subunit of the LVGCC $Ca_v1.2$, respectively. $Ca_v1.2$ channels are the primary mediators of depolarization-induced calcium entry into neurons. As such they play critical roles in the regulation of neuronal excitability (Lacinova *et al*, 2008), synaptic plasticity (Moosmang, 2005), learning and memory (White *et al*, 2008), and gene transcription (Dolmetsch, 2003). Particularly, well studied is their function in the promotion of dendrite growth and arborization in response to neuronal activity (Redmond & Ghosh, 2005). Thus, the convergence of several genetic associations into one common pathway implicates $Ca_v1.2$ channel dysfunction as a main genetic risk factor of BD. Concerning environmental factors, early life adversity, particularly childhood abuse and neglect, is known to correlate with unfavorable clinical outcomes in BD (Agnew-Blais & Danese, 2016). A common cellular endpoint of GxE risk factors in BD is defective neuroplasticity, in particular reductions of dendritic arborization and synapse density in several brain areas of BD patients, including the hippocampus (Phillips & Swartz, 2014). However, the molecular pathways which integrate GxE risk factors to induce impairments in neuroplasticity during BD are largely unknown.

MicroRNAs (miRNAs) are a large family of small, noncoding RNAs which act as posttranscriptional repressors of gene expression by binding to partially complementary sequences in the 3′ untranslated region (UTR) of target mRNAs (Bartel, 2018). In animals, miRNAs are widely expressed in the brain where they regulate various aspects of neuroplasticity, for example, dendritogenesis and dendritic spine development (Schratt, 2009), in an activity-dependent manner. A putative involvement of miRNAs in BD etiology is supported by several recent observations. First, differential expression of miRNAs is found in *post mortem* brain and blood of BD patients (Fries *et al*, 2018). Second, both circulating and brain miRNA levels are modulated by the intake of antidepressants (Lopez *et al*, 2017) and mood stabilizers (Chen *et al*, 2009; Zhou *et al*, 2009). Third, *in vivo* manipulation of specific miRNA candidates in rodents led to behavioral phenotypes with relevance to affect regulation and cognitive functioning via the modulation of serotonin, glucocorticoid, neurotrophic factor, and Wnt signaling pathways (Martins & Schratt, 2021). Lastly, variations in miRNA genes that confer susceptibility to BD have recently been identified (Forstner *et al*, 2015). However, it is still unknown how the dysregulation of specific miRNAs and associated pathways impairs neuronal function and contributes to BD.

In this study, we focused on miR-499-5p, a miRNA which after correction for multiple testing showed a nominally significant association with BD in a recent GWAS (Forstner *et al*, 2015). Interestingly, miR-499-5p plays important roles in the physiology and pathology of the cardiovascular system (van Rooij *et al*, 2009), but its function in the nervous system is completely unexplored. We found that miR-499-5p levels are strongly up-regulated in the blood of BD patients as well as in the hippocampus of socially isolated rats, an animal model of mental disorders. In hippocampal neurons, miR-499-5p targets the recently identified BD risk gene *Cacnb2* and controls dendritic development, $Ca_v1.2$ surface expression, and current density. In the *Cacna1c*$^{+/-}$ mental disorder rat model, overexpression of miR-499-5p in the hippocampus induced deficits in short-term recognition memory, providing a potential link between GxE risk factors. miR-499-5p levels are inversely correlated with gray matter volume (GMV) in the transverse/superior temporal gyrus of healthy subjects, suggesting a role for miR-499-5p in human neuroplasticity and cognition. Together, this suggests a novel mechanism whereby early life adversity induces excessive miR-499-5p expression, which in turn negatively impacts neuronal calcium homeostasis, neuroplasticity, and cognitive function.

Results

BD-associated miR-499-5p is expressed in rat hippocampal pyramidal neurons and functions as a negative regulator of dendritogenesis

Recently, variants in the *MIR499* gene have been associated with BD (Forstner *et al*, 2015). The main mature miRNA expressed from the MIR499 locus is miR-499-5p, whose function has been extensively studied in the cardiovascular system but very little in the brain. To investigate a possible involvement of miR-499-5p in the molecular pathophysiology of BD, we decided to study its function in the rat model which is easily amenable for experimental manipulation. We first measured miR-499-5p levels in different brain regions of the adult rat brain using qPCR. The expression of miR-499-5p was stably detected in all the tested brain regions (Fig 1A), including areas involved in the regulation of affect regulation and cognitive functioning, such as the hippocampus and frontal cortex (Millan *et al*, 2016; Toda *et al*, 2019). Juvenile social isolation (JSI) in rats is a common environmental model to study different facets of mental disorders, such as anxiety, drug addiction, and cognitive performance (Fig EV1A; Fone & Porkess, 2008; Valluy *et al*, 2015; Braun *et al*, 2019). As expected, JSI rats showed significantly lower

Figure 1. miR-499-5p is expressed in rat hippocampal pyramidal neurons and functions as a negative regulator of dendritogenesis. ▶

A miR-499-5p qPCR analysis using total RNA isolated from different areas of the adult female rat brain. U6 snRNA was used for normalization. Data are represented as bar graphs, mean ± SD (n = 3 animals).

B miR-499-5p qPCR analysis using total RNA isolated from the hippocampus of male rats that were either group-housed or socially isolated for 4 weeks postweaning. Data are represented as box plot with whiskers (+: mean, line: median; whiskers: Tukey) (n = 9 rats per group; Mann–Whitney U-test; ***P = 0.0008). Fold changes represent changes in gene expression relative to the control condition. U6 snRNA was used for normalization.

C Representative picture of single-molecule fluorescence *in situ* hybridization (smFISH) performed in rat hippocampal neurons at DIV7 using probes directed against miR-499-5p (red channel) and CamK2a (green channel) to identify excitatory neurons. Scale bar: 20 mm.

D Representative gray-scale images of primary rat hippocampal neurons (DIV 10) transfected with GFP and the indicated miRNA mimics. Scale bars: 50 μm.

E Quantification of the mean number of intersections by Sholl analysis. GFP-only transfected conditions were set to one in each experiment. Data are represented as scattered dot plots with bar, mean ± SD (n = 3; Unpaired two-sample t-test, *P = 0.0435).

F Representative gray-scale images of GFP-transfected *Cacna1c*$^{+/+}$ (WT) or *Cacna1c*$^{+/-}$ primary rat hippocampal neurons (DIV 10). Scale bars = 50 μm.

G Quantification of the mean number of intersections by Sholl analysis. Data are represented as scattered dot plots with bar, mean ± SD (n = 3 independent experiments; Unpaired two-sample t-test, **P = 0.0096). Fold changes represent changes in dendritic complexity relative to the control condition.

 © 2022 The Authors Published under the terms of the CC BY 4.0 license

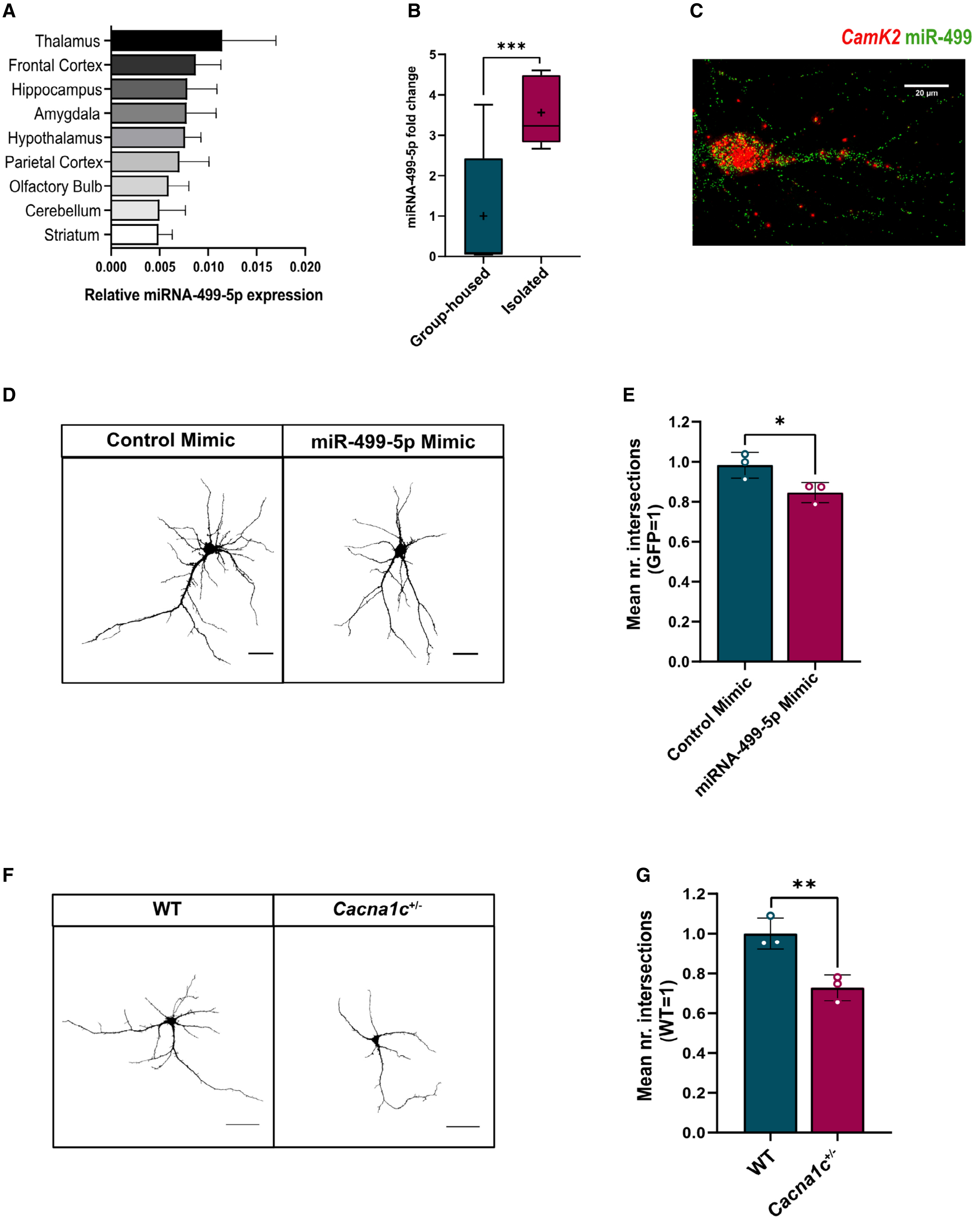
A
Thalamus
Frontal Cortex
Hippocampus
Amygdala
Hypothalamus
Parietal Cortex
Olfactory Bulb
Cerebellum
Striatum
0.000 0.005 0.010 0.015 0.020
Relative miRNA-499-5p expression
B
miRNA-499-5p fold change
***
Group-housed
Isolated
C
CamK2 miR-499
20 µm
D
Control Mimic
miR-499-5p Mimic
E
Mean nr. intersections (GFP=1)
*
Control Mimic
miRNA-499-5p Mimic
F
WT
Cacna1c+/-
G
Mean nr. intersections (WT=1)
**
WT
Cacna1c+/-

Figure 1.

hippocampal *c-fos* and *Arc* expression (Fig EV1B and C), neural activity-induced genes which are known to be down-regulated upon social isolation in the rat hippocampus (Pisu *et al*, 2011; Begni *et al*, 2020). Importantly, JSI induced a highly significant up-regulation (≈ 3.5 fold) in the expression of miR-499-5p compared to group-housed animals (Fig 1B). The JSI-mediated miRNA upregulation was specific for miR-499-5p, since hippocampal levels of two miRNAs previously implicated in psychiatric disorders (Xu *et al*, 2010; Lopez *et al*, 2017) were either reduced (miR-146a) or unaltered (miR-30e; Fig EV1D and E) upon JSI. Thus, upregulation of miR-499-5p in BD patients is paralleled by elevated miR-499 levels in the JSI rat model of mental disorders.

We next decided to study the function of miR-499-5p at the level of individual brain cells. Using smFISH in dissociated rat primary hippocampal neuron cultures, we detected miR-499-5p positive puncta (red, Fig 1C) in the soma and, to a lesser extent, dendrites of excitatory pyramidal neurons which co-expressed the marker Camk2a mRNA (green, Fig 1C). miR-499-5p expression increases during a time-course of hippocampal neuronal development from *DIV* 4–20 as assessed by qPCR (Fig EV1F), consistent with a function of miR-499-5p in dendrite and/or synapse development. This increase is also observed in glia-depleted cultures (Fig EV1F), demonstrating the neuronal source of miR-499-5p expression. Given our results obtained from JSI in rats (Fig 1B), we studied a potential regulation of miR-499-5p by stress signaling in rat hippocampal neuron cultures. Glucocorticoids (GCs) are released by the hypothalamic–pituitary–adrenocortical (HPA) axis in response to stressful events and act on neurons via mineralocorticoid (MRs) and glucocorticoid receptors (GRs) to modulate adaptive responses to stress (Finsterwald & Alberini, 2014; Herman *et al*, 2016). Moreover, high chronic exposure to GCs results in neurotoxicity in models of chronic stress and major depressive disorder (MDD; Dienes *et al*, 2013). We observed that a 5-day treatment of developing hippocampal neurons with the GR agonist Dexamethasone (DEX) significantly induced the expression of miR-499-5p (Fig EV1G), which suggests that stress signaling triggers miR-499-5p expression in neurons. Next, we explored potential effects of excessive expression of miR-499-5p, that is, observed by early life adversity or the activation of stress signaling on neuroplasticity. Therefore, we studied dendritogenesis as a paradigm since defects in dendritic arborization in the hippocampus are frequently observed in BD patients. To model excessive miR-499-5p activity, we transfected hippocampal neurons with miR-499-5p duplex oligonucleotides ("mimics"), which leads to a strong miR-499-5p overexpression (Fig EV1H). Sholl analysis revealed that hippocampal neurons transfected with a miR-499-5p mimic displayed a reduced number of intersections compared to control conditions (Figs 1D and E, and EV1I), indicating reduced dendritic arborization upon miR-499-5p overexpression. Calcium influx through LVGCCs is required for activity-dependent dendritogenesis (Redmond & Ghosh, 2005). In addition, both the $Ca_v1.2$ pore-forming *Cacna1c* and the regulatory beta-subunit *Cacnb2* are BD risk genes, and impaired cellular calcium homeostasis is a hallmark of BD (Harrison *et al*, 2019).This led us to hypothesize that $Ca_v1.2$ activity could be downstream of miR-499-5p in the control of dendritogenesis. In agreement with this hypothesis, hippocampal pyramidal neurons prepared from constitutive heterozygous *Cacna1c*$^{+/-}$ rats, a well-established mental disease animal model (Braun *et al*, 2018), displayed less complex dendritic branches than WT controls (Fig 1F and G), thereby mimicking the effect of miR-499-5p overexpression.

The BD risk gene *Cacbn2* is a downstream target of miR-499-5p in the regulation of hippocampal neuron dendritogenesis

We decided to further explore a potential direct regulation of $Ca_v1.2$ activity by miR-499-5p. By applying miRNA-binding site prediction tools to genes encoding $Ca_v1.2$ subunits, we detected a highly conserved miR-499-5p-binding site in the 3′UTR of *Cacnb2*, but not *Cacna1c* (Fig 2A). CACNB2 is a validated target of miR-499-5p in cardiomyocytes (Ling *et al*, 2017) and in a very recent GWAS with more than 40,000 BD patients, the CACNB2 locus was significantly associated with BD (Mullins *et al*, 2021). Therefore, we decided to investigate whether miR-499-5p and CACNB2 functionally interact in rat hippocampal neurons to control dendritogenesis.

We first studied the effect of miR-499-5p overexpression on Cacnb2 expression. Hippocampal neurons transfected with a miR-499-5p mimic exhibited a significant reduction in the levels of *Cacnb2* mRNA compared to neurons expressing the control mimic

Figure 2. The BD risk gene *Cacbn2* is a downstream target of miR-499-5p in the regulation of hippocampal neuron dendritogenesis.

A Nucleotide base pairing of miR-499-5p with *Cacnb2* 3′UTR (left) and schematic representation of the *Cacnb2* luciferase reporter (right).

B qPCR analysis of *Cacnb2* mRNA levels using total RNA isolated from rat hippocampal neurons transfected with either a control or miR-499-5p mimic. Data are represented as scattered dot plots with bar, mean ± SD (n = 3 independent experiments; Unpaired two-sample t-test, *P = 0.0363).

C qPCR analysis of *Cacnb2* mRNA levels using total RNA from the hippocampus of either socially isolated or group-housed rats. Data are represented as box plot with whiskers (+: mean, line: median; whiskers: Tukey) (n = 9 rats per group; Unpaired two-sample t-test, **P = 0.0020).

D Relative luciferase activity of rat hippocampal neurons transfected with the indicated miRNA mimics and expressing either a *Cacnb2* WT or MUT reporter. Data are represented as scattered dot plots with bar, mean ± SD (n = 4 independent experiments; Two-way ANOVA: main effect of the miRNA mimic P = 0.0087, of the *Cacnb2* luciferase reporter P < 0.0001 and of the miRNA mimic by Cacnb2 luciferase reporter interaction P < 0.0001, Tukey's HSD: ***P = 0.0002).

E Relative luciferase activity of rat hippocampal neurons transfected with the indicated pLNAs and expressing either a *Cacnb2* WT or MUT reporter. Data are represented as scattered dot plots with bar, mean ± SD (n = 4 independent experiments; Two-way ANOVA: main effect of the miRNA pLNA P = 0.0061, no main effect of the *Cacnb2* luciferase reporter P = 0.3590 or the miRNA pLNA by *Cacnb2* luciferase reporter P = 0.0910, Tukey's HSD: *P = 0.0152). Fold changes represent changes in relative luciferase activity of transfected neurons relative to the nontransfected neurons.

F Representative images of *DIV* 10 hippocampal neurons co-transfected with control or miR-499-5p mimics and a pMT2-*CACNB2* expression plasmid (*CACNB2*). Scale bars = 50 μm.

G Quantification of the mean number of intersections by Sholl analysis. Data are represented as scattered dot plots with bar, mean ± SD (n = 3 independent experiments; Two-way ANOVA: no main effect of the *CACNB2* transfection P = 0.5027 or the miRNA mimics P = 0.0805, main effect of the *CACBN2* transfection by miRNA mimics interaction P = 0.0213, Tukey's HSD: *P = 0.0303). Fold changes represent the changes in dendritic complexity of transfected neurons compared to nontreated control neurons. ns = not significant.

© 2022 The Authors Published under the terms of the CC BY 4.0 license

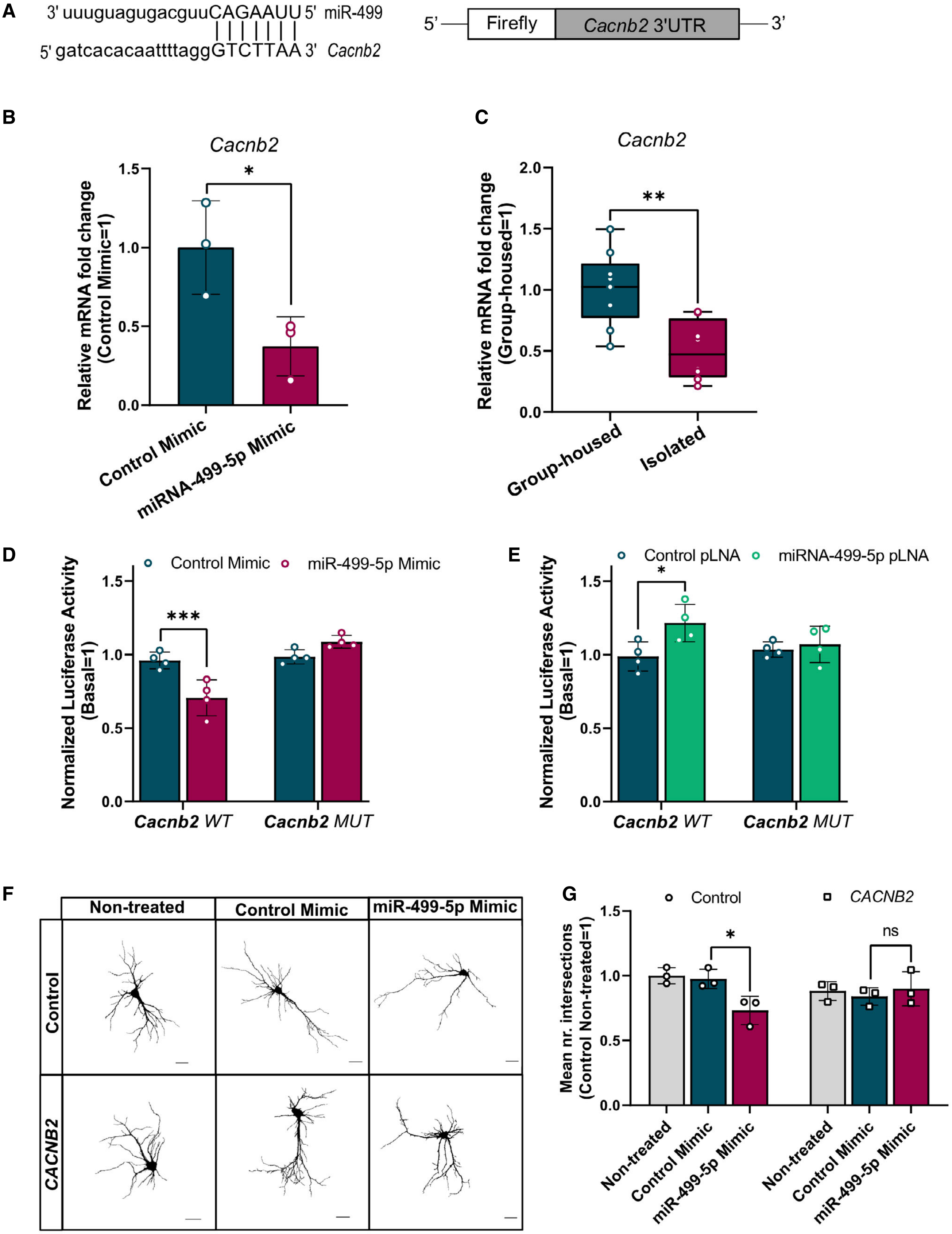
A
3' uuuguagugacguuCAGAAUU 5' miR-499
5' gatcacacaattttaggGTCTTAA 3' Cacnb2
5' Firefly Cacnb2 3'UTR 3'
B
Cacnb2
Relative mRNA fold change (Control Mimic=1)
Control Mimic
miRNA-499-5p Mimic
C
Cacnb2
Relative mRNA fold change (Group-housed=1)
Group-housed
Isolated
D
Control Mimic
miR-499-5p Mimic
Normalized Luciferase Activity (Basal=1)
Cacnb2 WT
Cacnb2 MUT
E
Control pLNA
miRNA-499-5p pLNA
Normalized Luciferase Activity (Basal=1)
Cacnb2 WT
Cacnb2 MUT
F
Non-treated
Control Mimic
miR-499-5p Mimic
Control
CACNB2
G
Control
CACNB2
Mean nr. intersections (Control Non-treated=1)
ns
Non-treated
Control Mimic
miR-499-5p Mimic

Figure 2.

as judged by qPCR (Fig 2B). The same treatment led to reduced Cacnb2 protein levels as assessed by Western blot, although the difference between miR-499-5p mimic and control transfected neurons did not reach statistical significance (Fig EV2A and B). Furthermore, *Cacnb2* mRNA levels were significantly lower in the hippocampus of JSI compared to group-housed rats (Fig 2C). The direction of change in socially isolated rats is opposite to the one observed for miR-499-5p (Fig 1B), consistent with a negative regulatory role for miR-499-5p in Cacnb2 expression. To test a possible direct interaction of miR-499-5p with the *Cacnb2* 3′UTR, we performed reporter

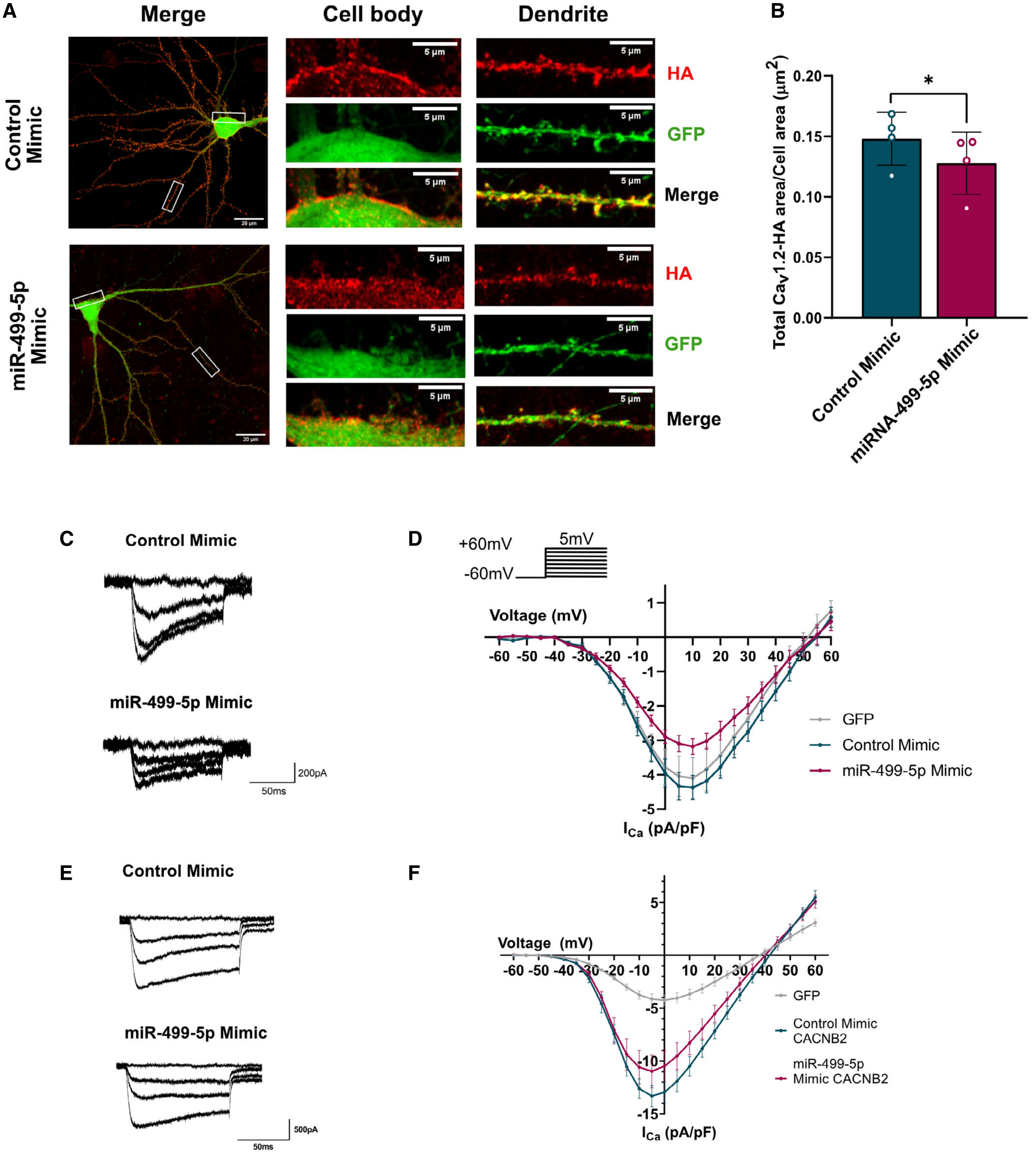

Figure 3.

 © 2022 The Authors Published under the terms of the CC BY 4.0 license

Figure 3. Elevated levels of miR-499-5p in hippocampal neurons impair cell surface expression and activity of $Ca_v1.2$ channels.

A Representative images of *DIV* 19 rat hippocampal neurons co-transfected with GFP (green channel) and Cav1.2-HA (red channel), together with either control or miR-499-5p mimics. After 12–13 days of expression, labeling with Anti-HA antibodies was performed in live conditions to identify cell surface Cav1.2 channels. Whole cells: scale bars = 20 μm; cell body and dendrite insets: scale bars = 5 μm.

B Quantification of the area occupied by surface Cav1.2 fluorescence normalized to the total cell area (GFP fluorescence) from nonpermeabilized neurons transfected as in (A). Data are represented as scattered dot plots with bar, mean ± SD (n = 4 independent experiments; Paired two-sample t-test *P = 0.0318).

C Representative calcium current traces from neurons transfected with the indicated miRNA mimics.

D I/V curves (current density against voltage) of calcium peak currents from hippocampal neurons co-transfected with GFP (n = 9 cells) and the Control (n = 10 cells) or miR-499-5p mimic (n = 10 cells) (P = 0.0188 for miR-499-5p mimic vs. Control mimic between −10 and + 30 mV, Unpaired two-sample t-test). Data are represented on XY graph as mean ± SEM.

E Representative calcium current traces from neurons transfected with the indicated miRNA mimics and pMT2-Cacnb2.

F I/V curves (current density against voltage) of calcium peak currents from hippocampal neurons co-transfected with GFP (n = 11 cells) and pMT2-Cacnb2 with the Control (n = 11 cells) or with miR-499-5p mimic (n = 12 cells) (n.s. P = 0.5379 for miR-499-5p mimic vs. Control mimic between −60 and + 60 mV, Unpaired two-sample t-test). Data are represented on XY graph as mean ± SEM.

gene assays in primary hippocampal neurons using luciferase genes fused to either the wild-type (WT) Cacnb2 3′UTR or Cacnb2 3′UTR containing mutations in the predicted miR-499-5p seed match (MUT; Fig 3C). Overexpression of miR-499-5p resulted in a significant repression of luciferase reporter gene expression for the *Cacnb2* WT plasmid (Fig 2D), consistent with a posttranscriptional, 3′UTR-dependent repressive effect of miR-499-5p on Cacnb2. This effect was not observed when the *Cacnb2* MUT plasmid was transfected (Fig 2D), demonstrating that miR-499-5p mediated its repressive effect via direct interaction with the seed match site in the *Cacnb2* 3′UTR. On the other hand, the inhibition of endogenous miR-499-5p in neurons by transfection of an LNA-modified antisense oligonucleotide (miR-499-5p pLNA) led to a significant increase in the expression of the *Cacnb2* WT, but not Cacnb2 MUT, luciferase reporter gene (Fig 2E). Together, these experiments establish Cacnb2 as a *bona-fide* miR-499-5p target gene in rat hippocampal neurons. We next investigated whether miR-499-5p-mediated regulation of Cacnb2 is functionally involved in dendritogenesis. Therefore, we transfected hippocampal neurons with a miR-499-5p mimic and examined whether restoring *CACNB2* expression by co-transfection of a Cacnb2 expression plasmid which lacks the 3′UTR (pMT2-CACNB2; Fig EV2C) was able to rescue impaired dendritogenesis observed upon miR-499-5p overexpression. Notably, whereas overexpression of miR-499-5p reduced dendritic complexity as expected, dendritic complexity of neurons co-transfected with miR-499-5p and pMT2-CACNB2 was not significantly different compared to control conditions (Fig 2F and G). This result provides strong evidence that *Cacnb2* is an important downstream component of the miR-499-5p-mediated regulation of dendritic development.

Elevated levels of miR-499-5p in hippocampal neurons impair cell surface expression and activity of $Ca_v1.2$ channels

The auxiliary β_2 subunit of LVGCCs encoded by the *Cacnb2* gene is an essential regulator of $Ca_v1.2$ cell surface expression (Bichet *et al*, 2000). To assess whether the regulation of *Cacnb2* by miR-499-5p had an effect on the surface expression of $Ca_v1.2$ channels, we performed anti-HA immunostaining on nonpermeabilized rat hippocampal neurons ("live staining") which had been transfected with an HA-tagged α_1 pore-forming subunit (Cacna1c-HA; Altier *et al*, 2002) together with miR-499-5p mimic. Since the Cacna1c-HA-epitope is only recognized once exposed on the cell surface, this procedure allows to measure $Ca_v1.2$ cell surface expression. In accordance with previous work showing a localization of $Ca_v1.2$ in proximal dendrites and cell bodies (Hell *et al*, 1993), we detected $Ca_v1.2$-HA puncta in both of these compartments (Fig 3A). However, surface expression of $Ca_v1.2$-HA was reduced upon overexpression of miR-499-5p as quantified by a significant decrease in the cell area covered by cell surface clusters of Cav1.2-HA channels (Fig 3B), integrated density (Fig EV3A), and $Ca_v1.2$-HA area (Fig EV3B). We did not observe any changes in cell area covered by total $Ca_v1.2$-HA, integrated density, or total Cav1.2 area (Fig EV3C–F) when we performed the HA staining under permeabilized conditions, suggesting that miR-499-5p overexpression did not affect overall levels of recombinant HA-tagged *Cacna1c*. Since $Ca_v1.2$ is the predominant LVGCC isoform in hippocampal neurons (54), we hypothesized that the observed reduction in cell surface expression of $Ca_v1.2$ channels translated into a corresponding loss of LVGCC currents ($I_{Ca,L}$). To test this hypothesis, whole-cell patch-clamp recordings were performed on hippocampal neurons overexpressing miR-499-5p. Calcium currents were evoked in conditions that allowed isolation of $I_{Ca,L}$ as indicated by a ≈ 50% reduction in current density by nifedipine treatment (Fig EV3G). Consistent with our results from immunostaining (Fig 3A and B), hippocampal neurons overexpressing miR-499-5p showed significantly smaller LVGCC current density than control neurons (Fig 3C and D). Neither channel activation (Fig EV3H) nor inactivation (Fig EV3I) was significantly altered by miR-499-5p overexpression, suggesting that miR-499-5p affected LVGCC activity primarily by regulating $Ca_v1.2$ membrane incorporation. Furthermore, transfection of the miR-499-5p mimic led to a decrease in membrane capacitance, which however did not reach statistical significance (Fig EV3J). This finding correlates well with reduced dendrite arborization observed upon miR-499-5p overexpression (Fig 1F and G), which is supposed to lead to an overall decrease in neuronal membrane area. We next asked whether re-expression of Cacnb2 in the context of miR-499 mimic was able to rescue impaired LVGCC currents, similar to our results obtained for dendritogenesis. In fact, Cacnb2 expression effectively prevented miR-499-5p-mediated reduction of LVGCC currents (Fig 3E and F), supporting the idea that Cacnb2 is an important functional target of miR-499-5p. Moreover, LVGCC currents were strongly elevated upon Cacnb2 overexpression in both control- and miR-499-5p mimic transfected neurons compared to controls, suggesting a gain-of-function effect. We conclude that miR-499-5p negatively

© 2022 The Authors Published under the terms of the CC BY 4.0 license

regulates the function of $Ca_v1.2$ calcium channels most likely by reducing their surface expression due to an inhibition of Cacnb2 expression.

miR-499-5p represses CACNB2 expression in the rat hippocampus *in vivo* and impairs short-term recognition memory in *Cacna1c*$^{+/-}$ rats

To further explore the impact of miR-499-5p dysregulation and $Ca_v1.2$ calcium channel dysfunction *in vivo*, we decided to overexpress miR-499-5p in the rat hippocampus. Therefore, a recombinant adeno-associated virus (rAAV) expressing a miR-499-5p precursor RNA together with eGFP under the control of the human synapsin promoter (AAV-miR-499) was bilaterally injected into the rat dorsal and ventral hippocampus of adult WT or Cacna1c$^{+/-}$ rats (Fig 4A; Appendix Fig S1). rAAV-mediated miR-499-5p delivery led to significant overexpression of miR-499-5p in both genotypes (Fig 4B and C; Appendix Fig S2A–D). CACNB2 protein levels, as assessed by Western blot, were significantly reduced in the hippocampus of both WT and Cacna1c$^{+/-}$ rats overexpressing miR-499-5p and negatively correlated with miR-499-5p levels (Figs 4D–F and EV4A and B). These findings confirm the negative regulation of CACNB2 by miR-499-5p in the rat brain *in vivo*.

We went on to investigate a potential role of elevated miR-499-5p for behavioral phenotypes associated with BD. For these experiments, we initially focused on the assessment of memory and anxiety, due to the known involvement of the hippocampus and LVGCC activity in these domains (Moosmang, 2005; Kabir *et al*, 2017; Dedic *et al*, 2018; Smedler *et al*, 2022). In the novel object recognition (NOR) test, which is affected by hippocampal lesions (Broadbent *et al*, 2010), both WT and Cacna1c$^{+/-}$ rats typically spent significantly more time exploring the novel object than the familiar one as measured by the increase in the time spent sniffing (percentage of total exploration) the novel object compared to the familiar object (Fig 4G). Overexpression of miR-499-5p in the hippocampus of WT animals did not alter the animal's preference for the novel object as the animals still spent significantly more time exploring the novel object. However, in *Cacna1c*$^{+/-}$ rats overexpressing miR-499-5p, the preference for the novel object was almost completely lost (Fig 4G). No differences were observed in the object acquisition phase of the test (Fig EV4C). To assess anxiety, we used the elevated plus maze (EPM) test, which is considered a gold standard in this domain. Neither the *Cacna1c* genotype nor miR-499-5p had an impact on the time rats spent in the open arm of the EPM (Fig 4H) or on their total entries into the open arms (Fig EV4D), indicating that anxiety-related behavior was not affected by miR-499-5p overexpression. Finally, we assessed motor activity as a potential confounder of the performance of rats in the NOR. No significant genotype- or miR-499-5p overexpression effects on the total distance traveled in the open field were observed (Fig EV4E), ruling out that impaired performance in the NOR test was due to motor dysfunction. When comparing male and female data (symbols), we did not obtain any indication for sex-dependent effects on any of the studied behaviors, although the sample size does not provide sufficient power for a conclusive statistical assessment. Taken together, miR-499-5p overexpression selectively impairs short-term recognition memory in the context of reduced $Ca_v1.2$ expression.

miR-499-5p expression is increased in the periphery of human BD patients

If altered miR-499-5p function is indeed involved in human BD etiology, one might expect alterations in miR-499-5p expression in BD patients compared to healthy controls. To address this hypothesis, we first assessed the levels of miR-499-5p in PBMCs obtained from BD patients, and as a comparison from MDD and SZ patients (Figs 5A and B, and EV5A; Appendix Tables S1 and S2). Peripheral levels of miR-499-5p were significantly increased in BD (Fig 5A),

Figure 4. miR-499-5p represses CACNB2 expression in the rat hippocampus *in vivo* and impairs short-term recognition memory in *Cacna1c*$^{+/-}$ rats. ▶

- A Schematic illustration of the hSyn-eGFP-scramble control (AAV-Control) and hSyn-eGFP-chimeric miR-499-5p hairpin (AAV-miR-499), and timeline of virus infection experiments.
- B miR-499-5p qPCR analysis from total RNA isolated from the right hippocampus of wild type rats injected with either AAV-control or AAV-miR-499. Data are represented as box plot with whiskers and data points (+: mean, line: median; whiskers: minimum and maximum values) (****$P < 0.0001$, Unpaired two-sample t-test; $n = 18$ rats in the AAV-Control group, $n = 16$ rats in the AAV-miR-499 group).
- C miR-499-5p qPCR analysis from total RNA isolated from the right hippocampus of Cacna1c$^{+/-}$ rats injected with either AAV-control or AAV-miR-499. Data are represented as box plot with whiskers and data points (+: mean, line: median; whiskers: minimum and maximum values) (****$P < 0.0001$, Unpaired two-sample t-test; $n = 15$ rats in the AAV-Control group, $n = 16$ rats in the AAV-miR-499 group).
- D Representative Western blot images of *CACNB2* (upper panel) and *GAPDH* (lower panel) protein expression levels in the hippocampus of WT rats and Cacna1c$^{+/-}$ rats injected with the AAV-Control or AAV-miR-499 hairpin viruses. GAPDH was used as a loading control.
- E Quantification of CACNB2 Western blots using hippocampal protein lysate from wild-type rats injected with either AAV-control or AAV-miR-499. Data are represented as box plot with whiskers and data points (+: mean, line: median; whiskers: minimum and maximum values) (*$P = 0.0103$, Unpaired two-sample t-test; $n = 7$ rats in the AAV-Control group, $n = 6$ rats in the AAV-miR-499 group).
- F Quantification of CACNB2 Western blots using hippocampal protein lysate from Cacna1c$^{+/-}$ rats injected with either AAV-control or AAV-miR-499. Data are represented as box plot with whiskers and data points (+: mean, line: median; whiskers: minimum and maximum values) (**$P = 0.0044$, Unpaired two-sample t-test; $n = 8$ rats in the AAV-Control group, $n = 7$ rats in the AAV-miR-499 group).
- G Novel object recognition task. Percentage of time WT or *Cacna1c*$^{+/-}$ rats injected with the indicated AAV explored either the familiar or novel object. Data are represented as box plot with whiskers and data points (+: mean, line: median; whiskers: minimum and maximum values) (WT_Control: $n = 18$ (eight males, 10 females); WT_miR-499: $n = 16$ (seven males, nine females); *Cacna1c*$^{+/-}$_Control: $n = 15$ (five males, 10 females); *Cacna1c*$^{+/-}$_miR-499: $n = 16$ (10 males, six females). Paired t-test – novel versus familiar object exploration percentage: WT_Control: $T_{17} = 2.383$, $P = 0.029$*; WT_miR-499: $T_{15} = 2.734$, $P = 0.015$*; *Cacna1c*$^{+/-}$_Control: $T_{14} = 2.849$, $P = 0.013$*; *Cacna1c*$^{+/-}$_miR-499: $T_{15} = 0.960$, $P = 0.352$).
- H Elevated plus maze test. Time spent in open arms did not differ between genotypes and treatment groups. Data are represented as box plot with whiskers and data points (+: mean, line: median; whiskers: minimum and maximum values) (One-way ANOVA; WT_Control: $n = 19$ (nine males, 10 females); WT_miR-499: $n = 16$ (seven males, nine females); *Cacna1c*$^{+/-}$_Control: $n = 17$ (seven males, 10 females); *Cacna1c*$^{+/-}$_miR-499: $n = 16$ (10 males, six females). Main effect Genotype: $F_{1, 64} = 0.044$, $P = 0.834$; main effect Treatment: $F_{1, 64} = 1.171$, $P = 0.283$; Interaction Genotype × Treatment: $F_{1, 64} = 0.076$, $P = 0.784$).

 © 2022 The Authors Published under the terms of the CC BY 4.0 license

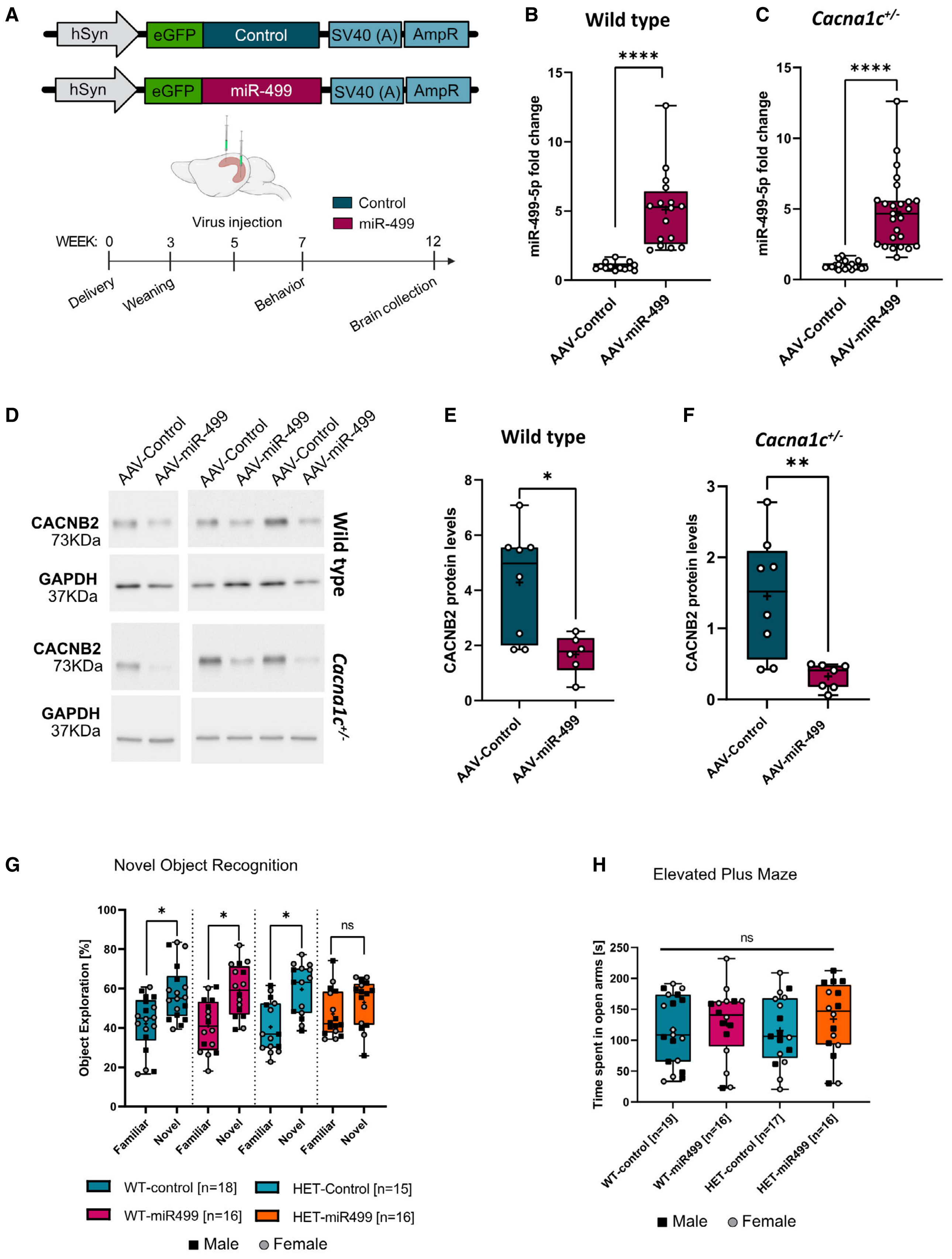
A
hSyn
eGFP
Control
SV40 (A)
AmpR
hSyn
eGFP
miR-499
SV40 (A)
AmpR
Control
miR-499
Virus injection
WEEK: 0 3 5 7 12
Delivery
Weaning
Behavior
Brain collection
B
Wild type
miR-499-5p fold change
AAV-Control
AAV-miR-499
C
Cacna1c+/-
D
CACNB2 73KDa
GAPDH 37KDa
Wild type
Cacna1c+/-
E
Wild type
CACNB2 protein levels
F
Cacna1c+/-
G
Novel Object Recognition
Object Exploration [%]
Familiar
Novel
ns
WT-control [n=18]
HET-Control [n=15]
WT-miR499 [n=16]
HET-miR499 [n=16]
Male
Female
H
Elevated Plus Maze
Time spent in open arms [s]
WT-control [n=19]
WT-miR499 [n=16]
HET-control [n=17]
HET-miR499 [n=16]

Figure 4.

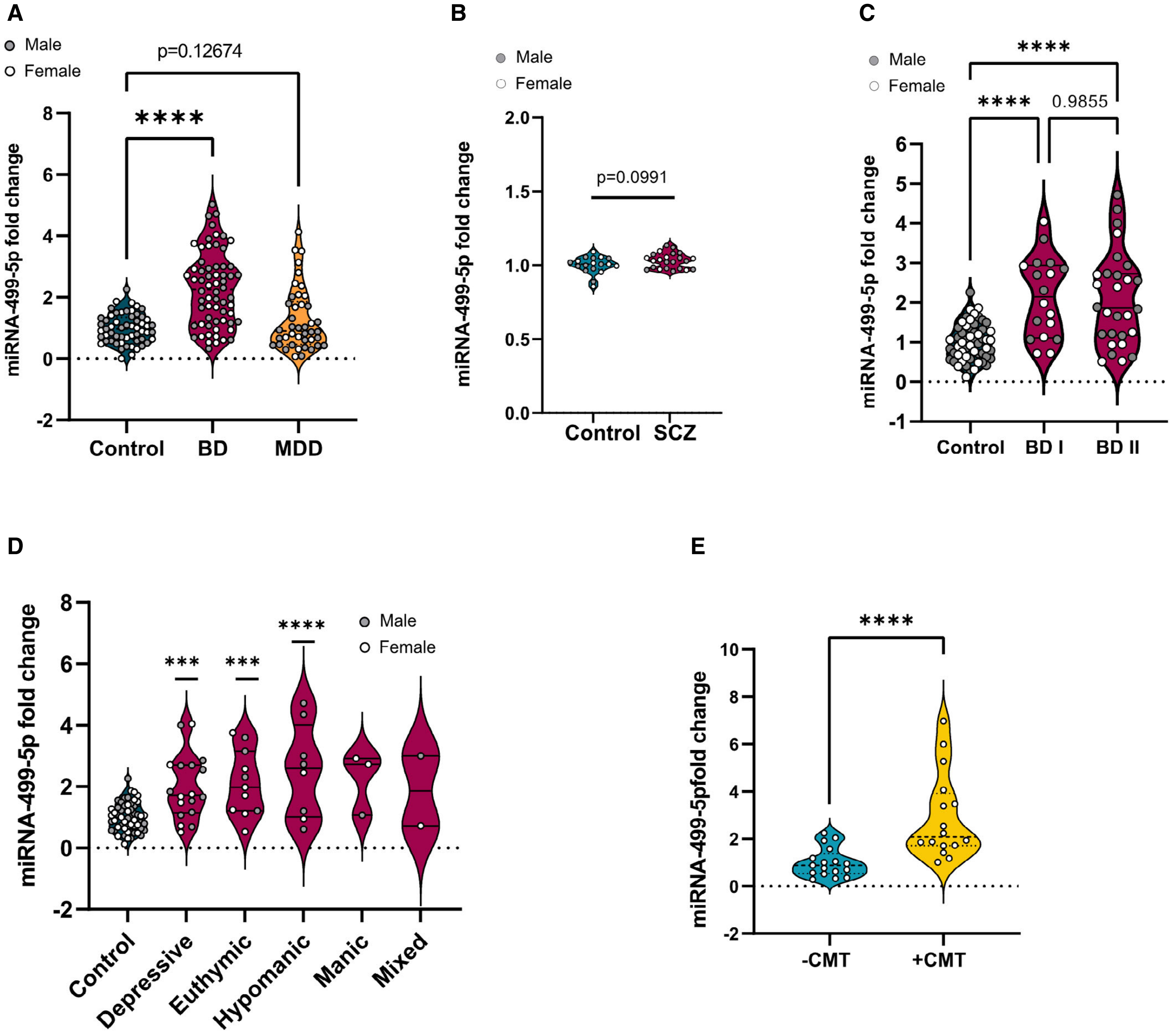

Figure 5. miR-499-5p expression is increased in human BD patients.

A miR-499-5p qPCR analysis of total RNA isolated from PBMCs of control (female = 26, male = 31), BD (female = 26, male = 37), or MDD (female = 18, male = 24) subjects. Wilcoxon rank sum test after correction for age and antidepressant treatment (linear model of the form Fold Change ~ Group*Sex + Age + Antidepressant treatment). Two-way ANOVA with correction for sex, age, and antidepressant treatment (linear model of the form Fold Change ~ Group*Sex + Age + Antidepressant treatment). Control versus BD: ****P = 8.89e-07; control versus MDD: n.s. P = 0.12674. Data are presented as violin plots with median, quartiles, and data points.

B miR-499-5p qPCR analysis of total RNA isolated from PBMCs of control (female = 8, male = 10) and SZ (female = 8, male = 15) subjects. Two-way ANOVA, Main effect Sex: P = 0.9785; main effect Group: P = 0.0924; main effect Interaction: P = 0.9802. Tukey's HSD: P = 0.0924, ns. Data are presented as violin plots with median, quartiles, and data points.

C miR-499-5p qPCR analysis of total RNA isolated from PBMCs of control (female = 26, male = 31), BD I (female = 9, male = 9), or BDI II (female = 12, male = 16) subjects. Two-way ANOVA, Main effect Sex: P = 0.174; main effect Group: ****P = 4.07e-09; Interaction Group × Sex: P = 0.311. Tukey's HSD: control versus BDI: ****P = 0.0000037, Control versus BDII: P = 0.0000002. Data are presented as violin plots with median, quartiles, and data points.

D miR-499-5p qPCR analysis of total RNA isolated from PBMCs of control subjects (female = 26, male = 31), and BD subjects in different mood states (depressive (female = 7, male = 11), euthymic (female = 7, male = 4), hypomanic (female = 2, male = 6), manic (female = 2, male = 1), mixed (female = 1, male = 1). Two-way ANOVA, main effect Sex: P = 0.260; main effect Group: P = 1.26e-07; Interaction Group × Sex: P = 0.059; Tukey's HSD: control versus depressive ***P = 0.0002621, control versus euthymic ***P = 0.0008533, control versus hypomanic ****P = 0.0000470. Data are presented as violin plots with median, quartiles, and data points.

E miR-499-5p qPCR analysis of total RNA isolated from PBMCs of healthy control subjects (−CMT, n = 17) and healthy subjects with a history of childhood maltreatment (+CMT, n = 17) (****P < 0.0001, Mann–Whitney U-test). CMT: Childhood maltreatment. Data are presented as violin plots with median, quartiles, and data points.

© 2022 The Authors Published under the terms of the CC BY 4.0 license

but not MDD and SZ patients (Fig 5B) compared to healthy controls (HC), after correction for age and antidepressant treatment (Fig EV5B and C). miR-499-5p was upregulated in both main BD subtypes, BDI and BDII (Fig 5C), and at different phases of BD (i.e., depressive, euthymic, hypomanic; Fig 5D). miR-499 upregulation in BD patients was observed independent of early life history, for example, childhood maltreatment (CMT; Fig EV5D), which by itself was however sufficient to trigger miR-499-5p expression in healthy subjects (Fig 5E, Appendix Table S3, Appendix Fig S3A–H). miR-499-5p levels in BD patients were not significantly correlated with the degree of manic symptomatology (as assessed by the Young Mania Rating Scale (YMRS; Fig EV5E)) and the severity of depressive symptoms (as assessed by the Beckś depression inventory (BDI; Fig EV5F)). Overall, no major differences in miR-499-5p expression between female and male BD patients were observed (Appendix Fig S4A–H).

In conclusion, peripheral miR-499-5p expression is specifically upregulated in BD patients independent of disease state or severity, supporting a role for miR-499-5p in human BD pathogenesis and identifying miR-499-5p as a novel trait biomarker candidate for BD.

miR-499-5p expression is negatively correlated with GMV of the left transverse/superior temporal gyrus in healthy humans

Given the negative regulatory role of miR-499-5p in dendritogenesis and cognition in rats, we explored a potential association between miR-499-5p levels and GMV in HC and BD subjects (Appendix Tables S1–S3), by performing multiple regression analyses with PBMC qPCR and structural MRI data (see Materials and Methods). In HC subjects, miR-499-5p levels were negatively associated with a GMV cluster comprising parts of the left Wernicke language area (i.e., temporal transverse gyrus (42%), the left parietal operculum (28%), and the left superior temporal gyrus (6%); $k = 1{,}090$, $x/y/z = -42/-30/15$, $t = 4.7$, $P = 0.045$ FWE cluster-level corrected; Fig 6A and B). No association was present for BD patients only and within the BD + HC analyses at the suggested statistical threshold. Our results are consistent with a role for miR-499-5p in the regulation of neuroplasticity in human brain areas associated with cognitive processing.

Discussion

A miR499-Cacnb2 pathway controls neuroplasticity and calcium homeostasis

Bipolar disorder is one of the most heritable neuropsychiatric disorders, and recent GWAS identified numerous genes whose products might be involved in the regulation of cellular functions related to BD, that is, calcium homeostasis and neuroplasticity. Nevertheless, we still know relatively little about specific gene regulatory pathways which link the expression of BD risk genes to cellular function and ultimately BD pathology. Here, we show that excessively high levels of the BD-associated miR-499-5p reduce expression of the auxiliary LVGCC subunit Cacnb2, which in turn leads to diminished surface expression of functional LVGCCs and reduced calcium currents. This likely results in a decrease in intracellular calcium, which in turn might impair downstream signaling events (e.g., CREB-dependent transcription) required for important aspects of neural circuit development, for example, activity-dependent dendritogenesis. The resulting defects in neuroplasticity might underlie cognitive and behavioral phenotypes associated with BD (Fig 6C).

Our result that the negative effects of miR-499-5p overexpression on dendritogenesis and Cav1.2-mediated calcium currents were rescued by co-expression of *CACNB2* strongly implicates LVGCC activity as an important downstream component of miR-499-dependent regulation of neuroplasticity. Previous studies already demonstrated an important role of LVGCC signaling in dendritogenesis in the context of neuropsychiatric disease. For example, inhibition of LVGCCs with Nifedipine significantly reduced dendritic growth (Redmond *et al*, 2002), and activation of the $Ca_v1.2$ downstream effectors CaMK, MAPK, and CREB are critical for dendritic growth (Redmond & Ghosh, 2005). Interestingly, iPSC-derived neurons from Timothy syndrome (TS) patients, which carry a gain-of-function mutation in the CACNA1C gene, are prone to activity-dependent dendrite retraction (Tian *et al*, 2014). Intriguingly, this phenotype does not depend on calcium influx through $Ca_v1.2$, but rather on the recruitment of the small Rho-family GTPAse Gem to $Ca_v1.2$ (Krey *et al*, 2013). By analogy, the reduction in *CACNB2* may impair dendritic growth by destabilizing the interaction between Gem and $Ca_v1.2$ calcium channels. In addition, the β_2 subunit is necessary for the trafficking of fully matured channel complexes from their site of folding to the cell membrane. Consistent with these observations, overexpression of miR-499-5p in hippocampal neurons led to reduced $Ca_v1.2$ cell surface expression and current density. In HEK293 cells, Cacnb2 increased the surface levels of Cav1.2 channels by preventing channel ubiquitination by the E3 ubiquitin ligase Ret Finger Protein 2 (RFP2) and entry into the ER-associated protein degradation system (Altier *et al*, 2011). Therefore, in addition to effects on forward trafficking, miR-499-5p overexpression might lead to increased Cacnb2 endocytosis followed by proteasome-mediated degradation.

Although an imbalance in cellular calcium homeostasis is widely accepted in the BD field, it is still controversial in which direction calcium concentrations are changed. Intracellular calcium levels are overall increased in the plasma and lymphocytes of BD patients but decreased in olfactory neurons (Harrison *et al*, 2019). In human iPSC-derived neurons from individuals carrying the *CACNA1C* risk allele rs1006737, higher mRNA levels of *CACNA1C* and higher L-type calcium currents compared to neurons carrying the nonrisk variant are observed (Yoshimizu *et al*, 2015). However, the *CACNA1C* risk allele rs1006737 has been shown to result in both gain- and loss-of-function of *CACNA1C* gene expression (Bigos *et al*, 2010; Gershon *et al*, 2014), depending on the brain region examined. Importantly, the effect of *Cacna1c* haploinsufficiency seems to depend on the developmental stage. Embryonic deletion of *Cacna1c* in forebrain glutamatergic neurons resulted in increased chronic stress susceptibility while during adulthood produced stress resilience (Dedic *et al*, 2018). Taken together, these observations suggest that calcium signaling alterations in both directions are possibly detrimental and can lead to defects in neuroplasticity, for example, dendritic impairments.

Mechanisms of miR-499-5p upregulation in the context of BD

Our observations that JSI induced the expression of miR-499-5p in rat hippocampus and that miR-499-5p is upregulated upon

© 2022 The Authors Published under the terms of the CC BY 4.0 license

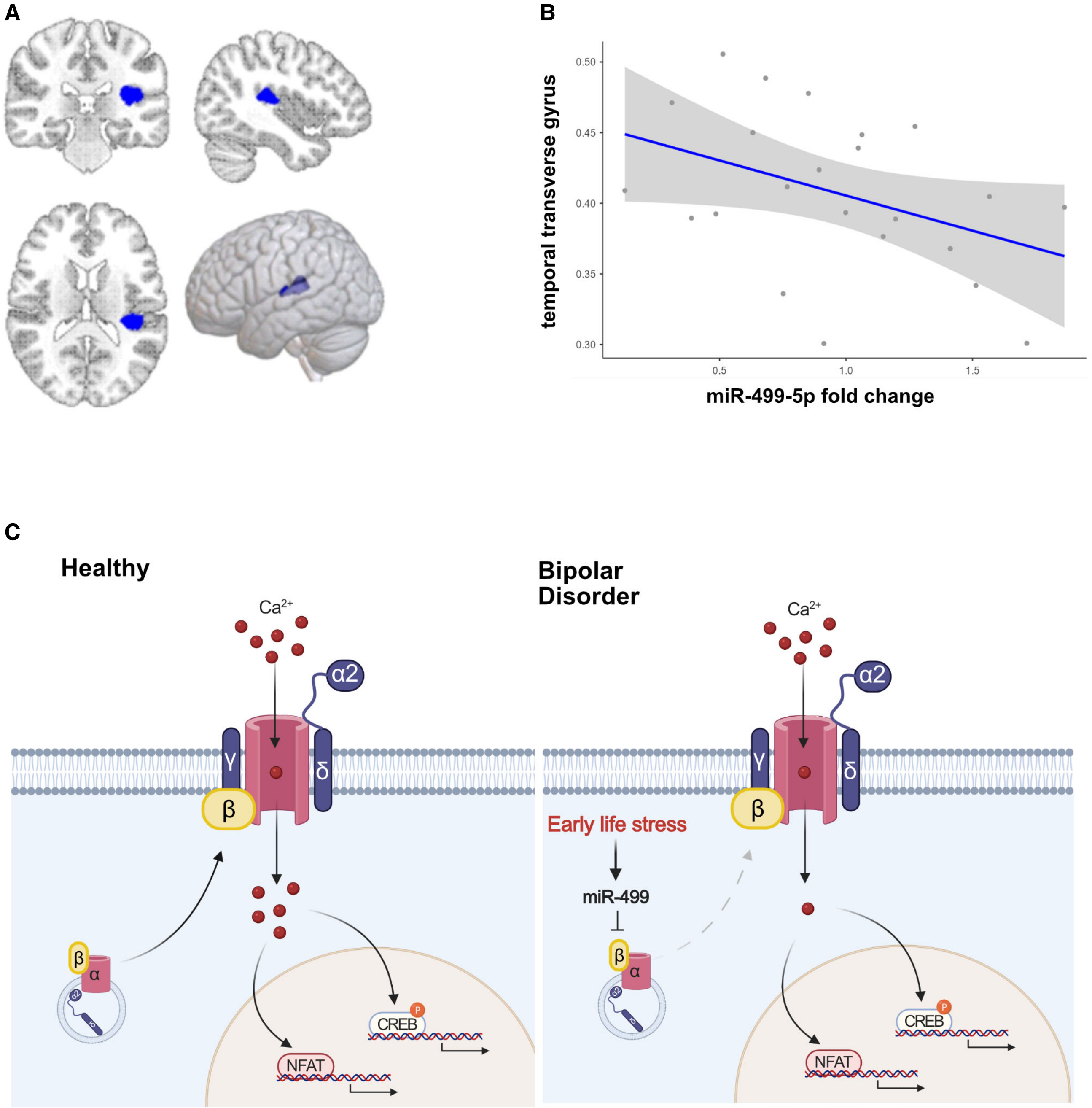

Figure 6. miR-499-5p expression is negatively correlated with GMV in healthy human subjects.

A, B Association of miR-499-5p expression and GMV in HC (n = 23): miR-499-5p levels were negatively correlated with a GMV cluster comprising parts of the left Wernicke language area (i.e., transverse temporal gyrus, the left parietal operculum, and the left superior temporal gyrus) (in blue) at $P < 0.05$ cluster-level family-wise error-corrected (FWE) for multiple comparisons after an initial threshold of $P < 0.001$ uncorrected (k = 1,090, $x/y/z$ = −42/−30/15, t = 4.7, P = 0.045).

C Proposed model for the mechanism of miR-499-5p dysregulation in BD. Early life stress induces the expression of miR-499-5p in human PBMCs and rat hippocampus, which impairs dendritic development and Cav1.2 calcium channel cell surface expression and activity by inhibiting the expression of an auxiliary subunit of Cav1.2 calcium channels and risk gene for BD, CACNB2.

dexamethasone treatment in primary rat neurons is consistent with a cell-autonomous regulation of miR-499-5p by stress-associated signaling in neurons. Interestingly, CMT similarly triggers miR-499-5p in PBMCs, suggesting that the gene regulatory pathways leading to miR-499-5p upregulation could be conserved between different cell types. However, miR-499-5p expression is increased in PBMCs of BD patients irrespective of a previous exposure to CMT (Fig 5D), suggesting that CMT is not the main driver of miR-499-5p

 © 2022 The Authors Published under the terms of the CC BY 4.0 license

expression in BD. Clearly, additional genetic and environmental factors involved in the control of miR-499-5p expression need to be revealed in future experiments. In this context, it is worth mentioning that re-sequencing of the miR-499-5p locus revealed several rare variants which are overrepresented in BD patients and might affect miR-499-5p biogenesis (Tielke A, Martins HC, Pelzl, MA, Maaser-Hecker A, David FS, Reinbold CS, Streit F, Sirignano L, Schwarz M, Vedder H, Kammerer-Ciernioch J, Albus M, Borrmann-Hassenbach M, Hautzinger M, Hünten K, Degenhardt F, Fischer SB, Beins EC, Herms S, Hoffmann P, Schulze TG, Witt SH, Rietschel M, Cichon S, Nöthen MN, Schratt G, Forstner AJ, unpublished data).

Activation of the MYH7B/MIR499 gene by environmental factors, for example, stress, could involve epigenetic modifications, since Tsumagari *et al* (2013) showed that exons 10, 17, and 18 of the MYH7B/MIR499 gene were hypomethylated in heart tissue compared to muscle progenitor cells, which correlated with higher miR-499-5p levels. On the other hand, miR-499-5p displays particularly high expression in the cardiovascular system (van Rooij *et al*, 2009). Thus, chronic stress might alternatively lead to aberrant miRNA-499-5p expression in the heart and secretion of miR-499-5p into the bloodstream inside exosomes that then reach the brain. In agreement with this hypothesis, dysregulated expression of miR-499-5p in prefrontal cortex (PFC) exosomes was previously reported in SZ and BD patients (Banigan *et al*, 2013). Exosomal transfer of miR-499-5p between the cardiovascular system and the brain would be especially relevant in light of the high co-morbidity between heart disease and BD (McIntyre *et al*, 2020). Indeed, higher levels of circulating miR-499-5p were reported in patients of acute myocardial infarction (Chen *et al*, 2015), cardiomyopathy (Matkovich *et al*, 2012), and atrial fibrillation (Ling *et al*, 2017).

miR-499-5p in neuropsychiatric disease

A few previous studies already indicated that alterations in miR-499-5p expression could contribute to the development of psychiatric disorders. For example, miR-499 is downregulated In exosomes derived from the PFC of SZ and BD patients (Banigan *et al*, 2013) and leukocytes from BD patients in depressive episodes (Banach *et al*, 2017). On the other hand, *post mortem* PFC of depressed subjects showed higher levels of miR-499-5p (Smalheiser *et al*, 2014). Here, we report upregulation of miR-499-5p in PBMCs of BD patients, but no significant effects in SZ or MDD patients, arguing for a rather specific involvement of miR-499-5p upregulation in BD. These inconsistencies between the different studies might be explained by the different organs (brain, blood) and miRNA sources (whole tissue, cells, exosomes) analyzed, as well as by the small sample sizes ($n < 15$) of the previous studies compared to ours. To further establish miR-499-5p as a BD trait biomarker, results should be replicated in different and even larger patient cohorts, ideally comparing miR-499-5p expression in different blood (PBMCs, serum, plasma, exosomes) and postmortem brain samples (PFC, ACC, amygdala, hippocampus).

miR-499-5p in rat and human cognition

Cognitive impairments, especially related to working memory and executive functioning, are among the core symptoms of BD in addition to mood disturbances (Solé *et al*, 2017). Our finding that overexpression of miR-499-5p in the hippocampus of *Cacna1c*$^{+/-}$ rats impairs short-term recognition memory argues in favor of an important contribution of miR-499 dysregulation to cognitive symptoms associated with BD. In the novel object recognition test, neither the genotype nor miR-499-5p overexpression alone affected the preference for the novel object compared to the familiar one. This observation agrees with a previous study which reported a normal preference for the novel object in *Cacna1c*$^{+/-}$ and WT rats of both sexes (Braun *et al*, 2018). Apparently, miR-499-5p-mediated reduction in calcium signaling is not sufficient to elicit LTP and associated memory impairments. In contrast, in *Cacna1c*$^{+/-}$ rats, overexpression of miR-499-5p might reduce the surface availability and activity of Ca_v1.2 channels to an extent where memory formation is no longer supported. *Cacna1c*$^{+/-}$ mice also showed deficits in spatial memory (Moosmang, 2005) and extinction of contextual fear (Temme & Murphy, 2017). Further behavioral tests are therefore warranted to obtain a more comprehensive picture of cognitive abilities of rats overexpressing miR-499-5p.

Besides cognition, dysregulation of emotional processing (e.g., mood instability, impulsivity, mania) is one of the hallmarks of BD. In this regard, deletion of *Cacna1c* in mice induces the expression of anxiety-like behaviors and has an anti-depressant effect (Dao *et al*, 2010), while in rats it was shown to impair social behavior (Kisko *et al*, 2018). In contrast, our preliminary behavioral testing did not provide indication for a role of miR-499-5p in anxiety-, depressive-, or manic behavior (Fig 4H, unpublished observation). However, we so far manipulated miR-499-5p exclusively in the hippocampus, which might not be the main brain area associated with these behaviors. In the future, miR-499-5p manipulation will be extended to cortical regions (PFC, ACC), amygdala and hypothalamus.

Intriguingly, our results from structural MRI suggest that miR-499-5p-mediated regulation of neuroplasticity might be conserved in humans. In agreement with the observed negative effect of miR-499-5p on dendritogenesis in rat hippocampal neurons, GMV in parts of the superior temporal gyrus (STG) was negatively correlated with miR-499-5p expression levels in healthy human subjects. The STG is involved in auditory processing, including language, but also has been implicated as a critical structure in social cognition. Reduced STG GMV has been observed in several neuropsychiatric disorders, including BD (Wang *et al*, 2019). In addition, impairments in auditory processing (Zenisek *et al*, 2015), verbal memory (Bora *et al*, 2009), and social cognition (Vlad *et al*, 2018) are frequently observed in BD. Together, these findings raise the possibility that chronically elevated miR-499-5p levels could underlie structural and cognitive impairments observed in BD patients.

In conclusion, we describe a novel mechanism for calcium signaling dysfunction in BD involving miRNA-dependent regulation of the BD risk gene *Cacnb2*. Stress-induced miR-499-5p represses *CACNB2* translation and reduces calcium influx required for dendritic development and cognitive function. Furthermore, circulating miR-499-5p could be used as a biomarker for BD diagnosis or to identify healthy individuals that are at elevated risk to develop a psychiatric condition and would therefore benefit from preventative therapies. Finally, miR-499-5p inhibition, for example, via stable brain delivery of a chemically modified, LNA-based miR-499-5p antisense oligonucleotide, could represent a new avenue for the treatment of cognitive impairments associated with BD and other neuropsychiatric disorders.

© 2022 The Authors Published under the terms of the CC BY 4.0 license

Materials and Methods

Human study

Bipolar disorder patients ($n = 26$ female, $n = 37$ male), MDD patients ($n = 18$ female, $n = 24$ female), and healthy controls ($n = 26$ female, $n = 31$ male), as well as healthy subjects with ($n = 17$) or without ($n = 18$) a history of childhood maltreatment, were obtained from the Departments of Psychiatry at the University of Marburg and the University of Münster, Germany, as part of the FOR2107 cohort (Kircher *et al*, 2019). The diagnosis was ascertained using the German version of the Structured Clinical Interview for DSM-IV (SCID-I; Wittchen *et al*, 1997) and subsequently translated to ICD-10 diagnoses (WHO, 1993). Subjects were excluded if they had substance-related disorders, severe neurological, or other medical disorders. Healthy control subjects underwent the same diagnosis procedure as the patients. Healthy subjects were included in the childhood maltreatment study if at least one subscale of the Childhood Trauma Questionnaire (CTQ) reached the threshold for maltreatment (Emotional Abuse ≥ 10, Physical Abuse ≥ 8, Sexual Abuse ≥ 8, Emotional Neglect ≥ 15, and Physical Neglect ≥ 8; Walker *et al*, 1999). The detailed demographic and clinical data are summarized in Appendix Tables S1 and S2. The procedures involving humans were approved by the ethics committees of the Medical Faculties of the Universities of Münster (2014-422-b-S) and Marburg (AZ: 07/14). Written informed consent was obtained from all participants before examination. Experiments conformed to the principles set out in the WMA Declaration of Helsinki and the Department of Health and Human Services Belmont Report.

Human peripheral blood mononuclear cell (PBMC) sample processing

Peripheral blood mononuclear cells were isolated from 10 ml of whole blood using the LeukoLOCK technology (Thermo Scientific) by the Biomaterialbank Marburg, Germany. Total RNA extraction from PBMCs was performed using the TRIzol™ Reagent (Thermo Fisher), according to the manufacturer's protocol. Samples were randomized using a webtool before RNA extraction.

MRI data acquisition, preprocessing, and statistical analyses

For BD patients ($n = 23$) and healthy controls ($n = 23$), multiple regression analyses were performed to investigate the association of miR-499-5p expression and gray matter volume (GMV). Therefore, T1-weighted images were acquired at two sites using a 3T MRI scanner (Münster: Prisma, Siemens, Erlangen, Germany, 20 channel head matrix Rx-coil; Marburg: Tim Trio, Siemens, Erlangen, Germany, 12 channel head matrix Rx-coil). MRI data were obtained according to an extensive quality assurance protocol (Vogelbacher *et al*, 2018). Image acquisition was performed using a fast gradient echo MP-RAGE sequence with a slice thickness of 1.0 mm consisting of 176 sagittal orientated slices in Marburg and 192 slices in Münster and a FOV of 256 mm and the following parameters at the two sites: Marburg: TR = 1.9 s, TE = 2.26 ms, TI = 900 ms, flip angle = 9°; Münster: TR = 2.13 s, TE = 2.28 ms, TI = 900 ms, flip angle = 8°. Images were preprocessed following the default parameters as implemented in the CAT12 toolbox (Computation Anatomy Toolbox for SPM, build 1184, Christian Gaser, Structural Brain Mapping group, Jena University Hospital, Germany; http://dbm.neuro.uni-jena.de/cat/) building on SPM12 (Statistical Parametric Mapping, Institute of Neurology, London, UK). In short, preprocessing included spatial registration, segmentation, and normalization of MRI data sets (Tohka *et al*, 2004; Ashburner & Friston, 2005; Ashburner, 2007). Smoothing was performed using a Gaussian kernel of 8 mm FWHM. After preprocessing three subjects of each group had to be excluded from brain structural analyses due to missing data or poor image quality.

Animal study

Ethics approval

All procedures were conducted in strict accordance with the National Institutes of Health Guidelines for the Care and Use of Laboratory Animals and the relevant local or national rules and regulations of Germany and were subject to prior authorization by the local government (MR 20/35 Nr. 19/2014 and G48/2019; Tierschutzbehörde, Regierungspräsdium Gieβen, Germany).

Cacna1c$^{+/-}$ animal breeding

Constitutive heterozygous *Cacna1c*$^{+/-}$ rats were generated by SAGE Labs (now Horizon Discovery Ltd.) on a Sprague Dawley background via zinc finger nucleases following a previously established protocol (Geurts *et al*, 2009). A heterozygous breeding protocol was used to obtain offspring from both *Cacna1c*$^{+/-}$ and *Cacna1c*$^{+/+}$ (WT) genotypes as previously established (Kisko *et al*, 2018). Rats were housed under standard laboratory conditions (22 ± 2°C and 40–70% humidity) with free access to standard rodent chow and water. Genotyping was performed as previously described (Kisko *et al*, 2018).

Juvenile social isolation paradigm

After weaning on PND 21, male rats were socially housed in groups of 4–6 with same-sex littermate partners (Group-housed, $n = 9$) or housed alone (Isolated, $n = 9$), applying a previously established protocol (Seffer *et al*, 2015). The hippocampus of the right hemispheres was removed immediately following the 4 weeks of exposure to the experimental housing conditions at ~2 months of age.

Rat primary cultures

Primary rat hippocampal and cortical neuronal cultures were prepared from E18 Sprague Dawley rats (Janvier Laboratories) as previously described (Schratt *et al*, 2006). For the preparation of primary cultures from *Cacna1c*$^{+/-}$ or WT rat embryos, the hippocampus from each embryo was dissected and collected separately for mechanical dissociation and plating while the cortex of each embryo was collected separately for genotyping. DNA was extracted from cortical tissue using the TriFast™ reagent protocol (VWR) and the *Cacna1c* gene was amplified with the PfuPlus! DNA Polymerase (Roboklon), according to the manufacturer's instructions, using the following primers: *Cacna1c* (369 bp) forward 5′-GCTGCTGAGCC TTTTATTGG-3′ and reverse 5′-GTCAGCAGCTATCCAGGAGG-3′.

Drug treatments

Neuronal-enriched cultures were obtained by treating cells with fluorodeoxyuridine (FUDR; Sigma) + uridine (Sigma; final concentration 10 μM) from the day *in vitro* 3 (DIV 3; glial-cell depleted). Treatment with Dexamethasone (DEX; Sigma) from DIV 5–10 to a

© 2022 The Authors Published under the terms of the CC BY 4.0 license

final concentration of 500 μM and re-applied every 2 days was used to activate glucocorticoid receptors (GRs; Seo *et al*, 2020). To control for vehicle effects, cells were treated with DMSO to a final volume concentration of 0.1% (v/v).

DNA constructs

The *Cacnb2* 3′UTR (Ensemble ID: ENSG00000165995) was amplified from a rat brain cDNA library and cloned into the pmirGLO dual-luciferase expression vector (Promega) using the PfuPlus! DNA Polymerase (Roboklon). Mutation of the miR-499-5p binding site was achieved using the Pfu Plus! DNA Polymerase Site-Directed Mutagenesis Protocol (Roboklon), according to manufacturer's instructions. For AAV-mediated overexpression of miR-499-5p *in vivo*, a chimeric miR-499 hairpin (AAV-miR-499) was generated as previously described (Christensen *et al*, 2010) Viral vectors were produced by the Viral Vector Facility of the Neuroscience Center Zurich (https://www.vvf.uzh.ch). The vector pβA-*CACNA1C*-HA ($Ca_v1.2$-HA) was kindly gifted by Prof. Amy Lee (Department of Neurology, University of Iowa, IA, USA). Primer sequences are provided in Appendix Table S3.

Transfections

Primary hippocampal neurons were transfected with plasmid DNA, miRNA mimics (Ambion™ Pre-miR miRNA Precursor, Thermo Fisher), and pLNAs (miRCURY LNA miRNA Power Inhibitors; QIAGEN) using the Lipofectamine 2000 reagent (Thermo Fisher). To measure *Cacnb2* mRNA levels upon miR-499-5p overexpression, hippocampal neurons were transfected at DIV7 with 10 nM of the miR-499-5p or control mimic (Ambion™ Pre-miR miRNA Precursor: miR-499-5p and Neg Control 1, Thermo Fisher) with the Lipofectamine RNAiMAX reagent (Thermo Fisher) and processed 7 days later for total RNA extraction. To validate the overexpression efficiency of the pMT2-*CACNB2* plasmid (Addgene, #107424), freshly isolated cortical neurons were transfected using the P3 Primary Cell 4D-Nucleofector Kit (Lonza, LZ-V4XP-3024) and the program DC-104 of the 4D-Nucleofector device (Lonza). After 5 DIV, cells were processed for Western Blot analysis.

Stereotaxic brain injections

Overexpression of miR-499-4p *in vivo* was carried out by injecting the chimeric miR-499 hairpin in the rat hippocampus as previously described (Zovoilis *et al*, 2011). Male and female WT and $Cacna1c^{+/-}$ rats (Sprague Dawley background) with 1.5–2 months were briefly anesthetized with isoflurane and placed in a stereotaxic frame. Stereotaxic surgery was performed under isoflurane anesthesia (Baxter Deutschland GmbH). For analgesia, animals received buprenorphine (0.05 mg/kg) 30 min before surgery and a subcutaneous injection of 0.4 ml of local anesthetic (Xylocaine 2% with Adrenaline 1:100,000) at the site of the incision immediately before surgery. Microinjections were delivered using a 30-gauge stainless steel infusion cannula connected to a 10-μl Hamilton syringe by polyethylene tube. One microliter of virus was injected in the dorsal hippocampus and 1 μl of virus in the ventral hippocampus (either AAV-miR-499 or AAV-Control) over 3 min via a microinjection pump (World Precision Instruments). The infusion cannula was left in place for an additional 3 min thereafter to allow diffusion. Hippocampal injections were performed bilaterally using the following coordinates, with bregma serving as reference: Dorsal hippocampus: anteroposterior = −3 mm, mediolateral = ±2 mm, and dorsoventral = +3.5 mm; Ventral hippocampus: anteroposterior = −4.8 mm, mediolateral = ±4.8 mm, and dorsoventral = +6.4 mm (Paxinos & Watson, 2007). Necessary pain management (buprenorphine) was applied, and the health of the animals was evaluated postoperative over the course of 7 consecutive days. The left hippocampus was freshly snap-frozen for biochemistry and the right hemisphere was fixed in 4% paraformaldehyde (PFA)-saline followed by dehydration in 30% sucrose-PBS solution for at least 2 days each. Brains mounted in tissue mounting fluid (Tissue-Tek O.C.T Compound, Sakura Finetek Europe B.V.) were sectioned (80 μm) using a cryostat (Histocom AG) and kept in a cryoprotectant solution for long-term storage. Images were acquired in a widefield microscope (Axio ObserverZ1/7, Zeiss).

Single-molecule fluorescence in situ hybridization (smFISH)

Single-molecule fluorescence in situ hybridization for miRNA detection on hippocampal cultures was performed using the QuantiGene ViewRNA miRNA Cell Assay Kit (Thermo Fisher) according to the manufacturer's protocol with slight modifications. To preserve dendrite morphology, protease treatment was reduced to a dilution of 1:10,000 in PBS for 45 s. The probes hsa-miR-499a-5p (Alexa Fluor 546; Thermo Fisher) and *CamK2* (Alexa Fluor 488; Thermo Fisher) were used for smFISH.

Immunostaining of primary cultures

Hippocampal neurons were co-transfected with 300 ng of $Ca_v1.2$-HA at DIV6 and 10 nM of the miR-499-5p or control mimic (AmbionTM Pre-miR miRNA Precursor: miR-499-5p and Neg Control 1, Thermo Fisher), and processed for immunostaining 13 days after. Cells were fixed in 4% PFA-4% sucrose in PBS for 15 min, rinsed with PBS, and permeabilized in 10% normal goat serum (NGS; vol/vol) containing 0.1% Triton (vol/vol) for 10 min. Blocking was performed for 30 min in 10% NGS. Cells were then sequentially labeled with Anti-HA High Affinity (1:100, Roche) and Anti-Rat, Alexa Fluor 546 (1:4,000, Thermo Fisher), both diluted in 10% NGS. After three washes with PBS, coverslips were mounted on microscope slides using AquaPoly/mount (Polysciences Inc.). Surface staining of $Ca_v1.2$-HA clusters was performed as previously described (Wang *et al*, 2017). Images were acquired in a confocal laser-scanning microscope (CLSM 880, Zeiss) and analyzed with a custom Python-script in the context of the ImageJ framework freely available via the ImageJ-update site (https://github.com/dcolam/Cluster-Analysis-Plugin).

Sholl analysis

Hippocampal neurons were transfected at DIV 5 with 100 ng of GFP alone or co-transfected with 10 nM of the miR-499-5p or control mimic (AmbionTM Pre-miR miRNA Precursor: miR-499-5p and Neg Control 1, Thermo Fisher). After 5 days of expression, the cells were fixed using 4% PFA-4% sucrose in PBS for 15 min, washed three times in PBS, and mounted on coverslips using Aqua-Poly/Mount (Chemie Brunschwig). Images were acquired in a widefield microscope (Axio ObserverZ1/7, Zeiss) and Sholl analysis was performed using the "Sholl Analysis" ImageJ plugin (Ferreira *et al*, 2014).

RT-qPCR

Total RNA extraction from brain tissue and primary cultures was performed using the TRIzol™ Reagent (Thermo Fisher) as per

© 2022 The Authors Published under the terms of the CC BY 4.0 license

manufacturer's instructions. RNA samples were first treated with the TURBO DNase enzyme (Thermo Fisher). To detect mRNAs, total RNA was reverse transcribed with the iScript cDNA synthesis kit (Bio-Rad) and RT-qPCR was performed using the iTaq SYBR Green Supermix with ROX (Bio-Rad) on the CFX384 Real-Time System (BioRad). To detect miRNAs, the TaqMan MicroRNA Reverse Transcription Kit (Thermo Fisher) and the TaqMan Universal PCR Master Mix (Thermo Fisher) were used, according to the manufacturer's instructions. Data were analyzed according to the ΔΔCt method, normalized first to the U6 snRNA housekeeping gene. Primer sequences are provided in Appendix Table S3.

Western blot

Proteins from hippocampal tissue were isolated from the phenol–ethanol supernatant saved from the RNA isolation using the TRIzol™ Reagent (Thermo Fisher) protocol, according to the manufacturer's directions. From neuronal cultures, protein extracts were obtained by lysing cells in lysis buffer (50 mM Tris-pH: 7.5, 150 mM NaCl, 1% Triton-X100, 1× Complete Protease Inhibitor Cocktail (Roche)). Typically, 10 μg of protein sample mixed with 4× Laemmli Sample Buffer (Bio-Rad) were separated on a 10% SDS-PAGE. After electrophoresis, proteins were transferred to a nitrocellulose membrane using a Trans-Blot Turbo system (Bio-Rad), according to the manufacturer's protocol. Membranes were blocked in 5% milk prepared in Tris-buffered saline containing 0.1% Tween20 and incubated in primary antibody solution overnight. Antibody dilutions (rabbit anti-*CACNB2* (1:1,000, Abcam), rabbit anti-*GAPDH* (1:2,000, Millipore), and mouse anti-*GFP* (1:1,000, Novus Biological)) were prepared in blocking solution. Membranes were washed five times in 5% milk before incubation in HRP (horseradish peroxidase)-conjugated secondary antibody prepared in blocking solution for 1 h. Following incubation, membranes were washed five times in TBS-T, developed with the Clarity™ Western ECL Substrate (Bio-Rad) according to manufacturer's guidelines, and visualized with the ChemiDocTM MP, Imaging System (BioRad).

Luciferase reporter assay

Hippocampal neurons were co-transfected at DIV 5 with 10 nM of the miR-499-5p or control mimic (AmbionTM Pre-miR miRNA Precursor: miR-499-5p or Neg Control 1, Thermo Fisher) and 100 ng of the *Cacnb2* 3′UTR luciferase reporters. To evaluate the effect of the miR-499-5p knockdown, neurons were co-transfected at DIV 19 with 10 nM of a miR-499-5p or control pLNA inhibitor (miRCURY LNA miRNA-499-5p Power Inhibitor: miR-499-5p or Neg Control A, QIAGEN). After 72 h of expression, cell lysis and luciferase assay were performed using the Dual-Luciferase Reporter Assay System (Promega), following a modified protocol (Baker & Boyce, 2014). Luciferase activity was measured on the GloMax Discover GM3000 (Promega), according to the manufacturer's instructions.

Electrophysiology

For the recording of ICa,L from primary cultured hippocampal neurons (DIV 15–16), transfected with the miR-499-5p or control mimic (AmbionTM Pre-miR miRNA Precursor: miR-499-5p or Neg Control 1, Thermo Fisher) for 5–6 days, whole-cell patch-clamp was performed as previously described (Hall *et al*, 2013). The extracellular solution was composed of (in mM) 110 NaCl, 2.5 KCl, 15 HEPES, 2 $CaCl_2$, 2 $MgCl_2$, 20 glucose, 20 TEA-Cl, and 5 4-AP (adjusted to pH 7.3 with NaOH) and intracellular solution 125 CsCl, 20 TEA-Cl, 0.5 EGTA, 10 HEPES, 4 Mg-ATP, 0.3 GTP (adjusted to pH 7.3 with CsOH). One microliter of TTX, 1 μM of Gabazine, 10 μM of CNQX, and 1 μM of ω-conotoxin (CTx) MVIIC, 1 μM of ω-CTxGVIA, and 30 μM of niflumic acid were added to the extracellular buffer to block to synaptic transmission, P/Q- and N-type Ca^{2+} channels and Ca^{2+}-activated Cl^{-} channels, respectively. Cells were held at −60 mV to inactivate T-type Ca^{2+} channels. Leak and capacitive currents were subtracted using the P/4 method. Series resistance compensation was enabled in all experiments (compensation 50–70%). The sampling frequency was 50 kHz and the filter frequency 10 kHz. For Ca^{2+} activation curves, Ca^{2+} currents were obtained by depolarizing cells from −60 mV to potentials between −60 and + 60 mV (in 5 mV increments). For Ca^{2+} inactivation, cells were held from -70 mV to 0 mV (in 5 mV increments), and after a depolarizing step to 10 mV cells were hyperpolarized to −70 mV. Peak Ca^{2+} currents were normalized to membrane capacitance and were plotted as a function of the membrane potential.

Behavioral paradigms

Approximately 1 week after injection of the chimeric miR-499-5p hairpin, male and female WT and *Cacna1c*$^{+/-}$ rats were tested in behavioral experiments during the light phase of a 12:12 h light/dark cycle (lights on at 07:00 h). Rats were handled for 3 consecutive days before behavioral testing in a standardized way for 5 min. Behavioral analyses were performed by an experienced observer blind to experimental conditions. In total, nine WT animals and five *Cacna1c*$^{+/-}$ animals were excluded from final statistical analyses since miR-499-5p expression did not exceed the mean expression of miR-499-5p + 2× SD of control animals (Appendix Figs S1 and S2A–D), resulting in inclusion of 36 WT and 33 *Cacna1c*$^{+/-}$ rats. We pooled sexes because in our previous study we did not obtain evidence for sex having a modulatory role on the effects of Cacna1c haploinsufficiency on object recognition memory, in our main outcome measure (Braun *et al*, 2018).

Open field test

General exploratory behavior was measured in rats on 2 consecutive days using an open field (40 cm × 40 cm × 40 cm) under dim red light, using a previously established protocol (Krug *et al*, 2020). Individual rats were placed randomly in one of the corners of the open field and were allowed to explore the apparatus for 10 min. Distance traveled (cm) were assessed on-line using an infrared sensor beam system mounted 2.5 cm above the floor of the arena (TruScan™, Photobeam Sensor-E63-22, Coulbourn Instruments, PA, USA).

Elevated plus maze

To measure anxiety-related behavior, rats were tested on an elevated plus maze (EPM), according to a previously established protocol (Krug *et al*, 2020). The EPM apparatus was made of plastic and consisted of two opposed open arms, two opposed enclosed arms (arm sizes: 50 cm × 10 cm), and an open square (10 cm × 10 cm) in the center. The maze, which was elevated 50 cm above the floor, was illuminated by white light (30 lux in the center of the maze) and was monitored through a video camera from above. The animals were placed into the center of the EPM, facing an open arm,

© 2022 The Authors Published under the terms of the CC BY 4.0 license

and had 5 min access to the maze. Number of entries into open or enclosed arms, and time spent on open or enclosed arms were analyzed from video files using Observer XT 12.5 software (Noldus Information Technology).

Novel object recognition

Recognition memory was assessed in male and female WT and *Cacna1c*$^{+/-}$ rats approximately 1 week after injection of the chimeric miR-499-5p hairpin. The novel object recognition test was performed as previously described (Braun *et al*, 2018). using Observer XT 12.5 software (Noldus Information Technology). One WT animal and two Cacna1c$^{+/-}$ animals were excluded from final analyses due to inadequate object exploration during the acquisition session.

Statistical analysis

Statistical tests were performed using SPSS and GraphPad Prism version 8.2.0 for Windows (GraphPad Software, San Diego, CA, USA, www.graphpad.com). The number of independent experiments is indicated in the plots. Bar graphs represent mean ± SD unless stated otherwise. Boxplots represent median (box: two quantiles around the median; whiskers: minimum and maximum value; points superimposed on the graph: individual values). Normality was tested using the Shapiro–Wilk test. Normally distributed data were tested using one or two sample Student's *t*-test (always two-sided) or ANOVA followed by *post hoc* Tukey test and otherwise for nonnormal data the nonparametric test Mann–Whitney U-test. Correlations were calculated using the Spearman correlation coefficient with two-tailed analysis. Significant changes in the BD and MDD patient group were determined by pairwise comparison using a non-parametric Wilcoxon rank sum test. To control for the effect of additional factors, a linear model of the form Fold Change ~ Group + Sex + Age + Antidepressant treatment was used. $P < 0.05$ was considered statistically significant. Correlational analyses of GMV and miR-499-5p expression in HC and BD subjects were performed using linear regression models in HC subjects, BD patients and HC + BD subjects in SPM 12 (v6906) running under Matlab. Covariates of no interest in the regression models were age, site, total intracranial volume, and the change of one gradient coil. As recommended for voxel-based-morphometry analyses, absolute threshold masking with a threshold value of 0.1 was used (http://dbm.neuro.uni-jena.de/cat/). Results were considered significant at $P < 0.05$ cluster-level family-wise error-corrected (FWE) for multiple comparisons after an initial threshold of $P < 0.001$ uncorrected. Significant clusters were labeled with the Dartel space Neuromorphometrics atlas (http://www.neuromorphometrics.com/).

Data availability

No large-scale datasets which need to be deposited in public repositories have been generated in the context of this study.

Expanded View for this article is available online.

Acknowledgements

The authors acknowledge the excellent technical assistance provided by Tatjana Wüst and Christina Furler. David Colameo wrote a Python script for Image Analysis. The authors are further grateful for the kind gift of the HA-Cacna1c expression plasmid by Amy Lee (Iowa). The authors thank Christine Hohmeyer, Marcella Rietschel, and Stephanie H. Witt for their help with genotyping and Christina Werning for help in data analysis. This work was supported by grants from the Schweizerischer Nationalfonds zur Förderung der Wissenschaftlichen Forschung (SNF 310030E_179651, 32NE30_189486) to GS. This work is further part of the Forschergruppe "Neurobiology of affective disorders: Translational perspectives on brain structure and function" (FOR 2107) and was supported by grants from the Deutsche Forschungsgemeinschaft to RS (DFG 559/14-1 and DFG 559/14-2), to MW (DFG WO 1732/4-1 and DFG WO 1732/4-2) and to TK (DFG KI 588/14-1 and KI 588/14-2). MW is further supported by grants from the Fonds Wetenschappelijk Onderzoek—Vlaanderen (FWO; Research Foundation—Flanders; e.g., G0C0522N) and a start-up grant at KU Leuven (PXF-E0120-STG/20/062). Open access funding provided by Eidgenossische Technische Hochschule Zurich.

Author contributions

Helena C Martins: Investigation; writing – original draft. **A Özge Sungur:** Supervision; investigation. **Carlotta Gilardi:** Investigation; writing – review and editing. **Michael A Pelzl:** Investigation. **Silvia Bicker:** Investigation; methodology. **Fridolin Gross:** Formal analysis. **Jochen Winterer:** Supervision; investigation; methodology. **Theresa M Kisko:** Investigation. **Natalia Malikowska-Racia:** Investigation. **Moria D Braun:** Investigation. **Katharina Brosch:** Investigation. **Igor Nenadic:** Supervision. **Frederike Stein:** Data curation; formal analysis. **Susanne Meinert:** Data curation; formal analysis. **Rainer KW Schwarting:** Conceptualization; supervision. **Udo Dannlowski:** Conceptualization; supervision. **Tilo Kircher:** Conceptualization; supervision; project administration. **Markus Wöhr:** Conceptualization; supervision; writing – review and editing. **Gerhard Schratt:** Conceptualization; supervision; writing – original draft; writing – review and editing.

Disclosure and competing interests statement

The authors declare that they have no conflict of interest.

References

Agnew-Blais J, Danese A (2016) Childhood maltreatment and unfavourable clinical outcomes in bipolar disorder: a systematic review and meta-analysis. *Lancet Psychiatry* 3: 342 – 349

Altier C, Dubel SJ, Barrère C, Jarvis SE, Stotz SC, Spaetgens RL, Scott JD, Cornet V, De Waard M, Zamponi GW *et al* (2002) Trafficking of L-type calcium channels mediated by the postsynaptic scaffolding protein AKAP79. *J Biol Chem* 277: 33598 – 33603

Altier C, Garcia-Caballero A, Simms B, You H, Chen L, Walcher J, Tedford HW, Hermosilla T, Zamponi GW (2011) The Cavβ subunit prevents RFP2-mediated ubiquitination and proteasomal degradation of L-type channels. *Nat Neurosci* 14: 173 – 182

Ashburner J (2007) A fast diffeomorphic image registration algorithm. *Neuroimage* 38: 95 – 113

Ashburner J, Friston KJ (2005) Unified segmentation. *Neuroimage* 26: 839 – 851

Baker JM, Boyce FM (2014) High-throughput functional screening using a homemade dual-glow luciferase assay. *J Vis Exp* 88: 50282

Banach E, Dmitrzak-Weglarz M, Pawlak J, Kapelski P, Szczepankiewicz A, Rajewska-Rager A, Slopien A, Skibinska M, Czerski P, Hauser J (2017) Dysregulation of miR-499, miR-708 and miR-1908 during a depression episode in bipolar disorders. *Neurosci Lett* 654: 117 – 119

© 2022 The Authors Published under the terms of the CC BY 4.0 license

Banigan MG, Kao PF, Kozubek JA, Winslow AR, Medina J, Costa J, Schmitt A, Schneider A, Cabral H, Cagsal-Getkin O *et al* (2013) Differential expression of Exosomal microRNAs in prefrontal cortices of schizophrenia and bipolar disorder patients. *PLoS One* 8: e48814

Bartel DP (2018) Metazoan microRNAs. *Cell* 173: 20 – 51

Begni V, Sanson A, Pfeiffer N, Brandwein C, Inta D, Talbot SR, Riva MA, Gass P, Mallien AS (2020) Social isolation in rats: effects on animal welfare and molecular markers for neuroplasticity. *PLoS One* 15: e0240439

Bichet D, Cornet V, Geib S, Carlier E, Volsen S, Hoshi T, Mori Y, De Waard M (2000) The I-II loop of the ca^{2+} channel α 1 subunit contains an endoplasmic reticulum retention signal antagonized by the β subunit. *Neuron* 25: 177 – 190

Bigos KL, Mattay VS, Callicott JH, Straub RE, Vakkalanka R, Kolachana B, Hyde TM, Lipska BK, Kleinman JE, Weinberger DR (2010) Genetic variation in CACNA1C affects brain circuitries related to mental illness. *Arch Gen Psychiatry* 67: 939 – 945

Bora E, Yucel M, Pantelis C (2009) Cognitive endophenotypes of bipolar disorder: a meta-analysis of neuropsychological deficits in euthymic patients and their first-degree relatives. *J Affect Disord* 113: 1 – 20

Braun MD, Kisko TM, Vecchia DD, Andreatini R, Schwarting RKW, Wöhr M (2018) Sex-specific effects of Cacna1c haploinsufficiency on object recognition, spatial memory, and reversal learning capabilities in rats. *Neurobiol Learn Mem* 155: 543 – 555

Braun MD, Kisko TM, Witt SH, Rietschel M, Schwarting RKW, Wohr M (2019) Long-term environmental impact on object recognition, spatial memory and reversal learning capabilities in Cacna1c-haploinsufficient rats. *Hum Mol Genet* 28: 4113 – 4131

Broadbent NJ, Gaskin S, Squire LR, Clark RE (2010) Object recognition memory and the rodent hippocampus. *Learn Mem* 17: 5 – 11

Chen H, Wang N, Burmeister M, McInnis MG (2009) MicroRNA expression changes in lymphoblastoid cell lines in response to lithium treatment. *Int J Neuropsychopharmacol* 12: 975 – 981

Chen X, Zhang L, Su T, Li H, Huang Q, Wu D, Yang C, Han Z (2015) Kinetics of plasma microRNA-499 expression in acute myocardial infarction. *J Thorac Dis* 7: 890 – 896

Christensen M, Larsen LA, Kauppinen S, Schratt G (2010) Recombinant adeno-associated virus-mediated microRNA delivery into the postnatal mouse brain reveals a role for miR-134 in dendritogenesis *in vivo*. *Front Neural Circuits* 3: 16

Cross-Disorder Group of the Psychiatric Genomics Consortium, Lee SH, Ripke S, Neale BM, Faraone SV, Purcell SM, Perlis RH, Mowry BJ, Thapar A, Goddard ME *et al* (2013) Genetic relationship between five psychiatric disorders estimated from genome-wide SNPs. *Nat Genet* 45: 984 – 994

Dao DT, Mahon PB, Cai X, Kovacsics CE, Blackwell RA, Arad M, Shi J, Zandi PP, O'Donnell P, Knowles JA *et al* (2010) Mood disorder susceptibility gene CACNA1C modifies mood-related behaviors in mice and interacts with sex to influence behavior in mice and diagnosis in humans, biological psychiatry. *Biol Psychiatry* 68: 801 – 810

Dedic N, Pöhlmann ML, Richter JS, Mehta D, Czamara D, Metzger MW, Dine J, Bedenk BT, Hartmann J, Wagner KV *et al* (2018) Cross-disorder risk gene CACNA1C differentially modulates susceptibility to psychiatric disorders during development and adulthood. *Mol Psychiatry* 23: 533 – 543

Dienes KA, Hazel NA, Hammen CL (2013) Cortisol secretion in depressed, and at-risk adults. *Psychoneuroendocrinology* 38: 927 – 940

Dolmetsch R (2003) Excitation-transcription coupling: signaling by ion channels to the nucleus. *Sci Signal* 2003: PE4

Dong M, Lu L, Zhang L, Zhang Q, Ungvari GS, Ng CH, Yuan Z, Xiang Y, Wang G, Xiang Y-T (2020) Prevalence of suicide attempts in bipolar disorder: a systematic review and meta-analysis of observational studies. *Epidemiol Psychiatr Sci* 29: e63

Ferreira TA, Blackman AV, Oyrer J, Jayabal S, Chung AJ, Watt AJ, Sjöström PJ, van Meyel DJ (2014) Neuronal morphometry directly from bitmap images. *Nat Methods* 11: 982 – 984

Finsterwald C, Alberini CM (2014) Stress and glucocorticoid receptor-dependent mechanisms in long-term memory: from adaptive responses to psychopathologies. *Neurobiol Learn Mem* 112: 17 – 29

Fone KCF, Porkess MV (2008) Behavioural and neurochemical effects of post-weaning social isolation in rodents—relevance to developmental neuropsychiatric disorders. *Neurosci Biobehav Rev* 32: 1087 – 1102

Forstner AJ, Hofmann A, Maaser A, Sumer S, Khudayberdiev S, Mühleisen TW, Leber M, Schulze TG, Strohmaier J, Degenhardt F *et al* (2015) Genome-wide analysis implicates microRNAs and their target genes in the development of bipolar disorder. *Transl Psychiatry* 5: e678

Fries GR, Carvalho AF, Quevedo J (2018) The miRNome of bipolar disorder. *J Affect Disord* 233: 110 – 116

Gershon ES, Grennan K, Busnello J, Badner JA, Ovsiew F, Memon S, Alliey-Rodriguez N, Cooper J, Romanos B, Liu C (2014) A rare mutation of CACNA1C in a patient with bipolar disorder, and decreased gene expression associated with a bipolar-associated common SNP of CACNA1C in brain. *Mol Psychiatry* 19: 890 – 894

Geurts AM, Cost GJ, Freyvert Y, Zeitler B, Miller JC, Choi VM, Jenkins SS, Wood A, Cui X, Meng X *et al* (2009) Knockout rats via embryo microinjection of zinc-finger nucleases. *Science* 325: 433

Hall DD, Dai S, Tseng P-Y, Malik Z, Nguyen M, Matt L, Schnizler K, Shephard A, Mohapatra DP, Tsuruta F *et al* (2013) Competition between α-actinin and Ca^{2+}-Calmodulin Controls Surface Retention of the L-type Ca^{2+} Channel CaV1.2. *Neuron* 78: 483 – 497

Harrison PJ, Hall N, Mould A, Al-Juffali N, Tunbridge EM (2019) Cellular calcium in bipolar disorder: systematic review and meta-analysis. *Mol Psychiatry* 26: 4106 – 4116

Hell JW, Westenbroek RE, Warner C, Ahlijanian MK, Prystay W, Gilbert MM, Snutch TP, Catterall WA (1993) Identification and differential subcellular localization of the neuronal class C and class D L-type calcium channel α1 subunits. *J Cell Biol* 123: 949 – 962

Herman JP, McKlveen JM, Ghosal S, Kopp B, Wulsin A, Makinson R, Scheimann J, Myers B (2016) *Regulation of the hypothalamic-Pituitaryr-adrenocortical stress response, comprehensive physiology*, pp 603 – 621. Hoboken, NJ: John Wiley & Sons, Inc.

Johansson V, Kuja-Halkola R, Cannon TD, Hultman CM, Hedman AM (2019) A population-based heritability estimate of bipolar disorder – In a Swedish twin sample. *Psychiatry Res* 278: 180 – 187

Kabir ZD, Che A, Fischer DK, Rice RC, Rizzo BK, Byrne M, Glass MJ, De Marco Garcia NV, Rajadhyaksha AM (2017) Rescue of impaired sociability and anxiety-like behavior in adult cacna1c-deficient mice by pharmacologically targeting eIF2alpha. *Mol Psychiatry* 22: 1096 – 1109

Kircher T, Wohr M, Nenadic I, Schwarting R, Schratt G, Alferink J, Culmsee C, Garn H, Hahn T, Muller-Myhsok B *et al* (2019) Neurobiology of the major psychoses: a translational perspective on brain structure and function-the FOR2107 consortium. *Eur Arch Psychiatry Clin Neurosci* 269: 949 – 962

Kisko TM, Braun MD, Michels S, Witt SH, Rietschel M, Culmsee C, Schwarting RKW, Wöhr M (2018) Cacna1c haploinsufficiency leads to pro-social 50-kHz ultrasonic communication deficits in rats. *Dis Model Mech* 11: dmm034116

Krey JF, Paşca SP, Shcheglovitov A, Yazawa M, Schwemberger R, Rasmusson R, Dolmetsch RE (2013) Timothy syndrome is associated with activity-

 © 2022 The Authors Published under the terms of the CC BY 4.0 license

dependent dendritic retraction in rodent and human neurons. *Nat Neurosci* 16: 201 – 209

Krug A, Wohr M, Seffer D, Rippberger H, Sungur AO, Dietsche B, Stein F, Sivalingam S, Forstner AJ, Witt SH *et al* (2020) Advanced paternal age as a risk factor for neurodevelopmental disorders: a translational study. *Mol Autism* 11: 54

Lacinova L, Moosmang S, Langwieser N, Hofmann F, Kleppisch T (2008) Cav1.2 calcium channels modulate the spiking pattern of hippocampal pyramidal cells. *Life Sci* 82: 41 – 49

Ling T-Y, Wang X-L, Chai Q, Lu T, Stulak JM, Joyce LD, Daly RC, Greason KL, Wu L-Q, Shen W-K *et al* (2017) Regulation of cardiac CACNB2 by microRNA-499: potential role in atrial fibrillation. *BBA Clin* 7: 78 – 84

Lopez JP, Fiori LM, Cruceanu C, Lin R, Labonte B, Cates HM, Heller EA, Vialou V, Ku SM, Gerald C *et al* (2017) MicroRNAs 146a/b-5 and 425-3p and 24-3p are markers of antidepressant response and regulate MAPK/Wnt-system genes. *Nat Commun* 8: 15497

Martins HC, Schratt G (2021) MicroRNA-dependent control of neuroplasticity in affective disorders. *Transl Psychiatry* 11: 263

Matkovich SJ, Hu Y, Eschenbacher WH, Dorn LE, Dorn GW (2012) Direct and indirect involvement of MicroRNA-499 in clinical and experimental cardiomyopathy. *Circ Res* 111: 521 – 531

McIntyre RS, Berk M, Brietzke E, Goldstein BI, López-Jaramillo C, Kessing LV, Malhi GS, Nierenberg AA, Rosenblat JD, Majeed A *et al* (2020) Bipolar disorders. *Lancet* 396: 1841 – 1856

Millan MJ, Rivet J-M, Gobert A (2016) The frontal cortex as a network hub controlling mood and cognition: probing its neurochemical substrates for improved therapy of psychiatric and neurological disorders. *J Psychopharmacol* 30: 1099 – 1128

Seo MK, Hien LT, Park MK, Choi AJ, Seog D-H, Kim S-H, Park SW, Lee JG (2020) AMPA receptor-mTORC1 signaling activation is required for neuroplastic effects of LY341495 in rat hippocampal neurons. *Sci Rep* 10: 993

Moosmang S (2005) Role of hippocampal Cav1.2 Ca^{2+} channels in NMDA receptor-independent synaptic plasticity and spatial memory. *J Neurosci* 25: 9883 – 9892

Moskvina V, Craddock N, Holmans P, Nikolov I, Pahwa JS, Green E, Owen MJ, O'Donovan MC (2009) Gene-wide analyses of genome-wide association data sets: evidence for multiple common risk alleles for schizophrenia and bipolar disorder and for overlap in genetic risk. *Mol Psychiatry* 14: 252 – 260

Mullins N, Forstner AJ, O'Connell KS, Coombes B, Coleman JRI, Qiao Z, Als TD, Bigdeli TB, Børte S, Bryois J *et al* (2021) Genome-wide association study of more than 40,000 bipolar disorder cases provides new insights into the underlying biology. *Nat Genet* 53: 817 – 829

Paxinos G, Watson C (2007) *The rat brain in stereotaxic coordinates*, 6th edn, p 456 . San Diego, CA: Academic Press

Phillips ML, Swartz HA (2014) A critical appraisal of neuroimaging studies of bipolar disorder: toward a new conceptualization of underlying neural circuitry and a road map for future research. *Am J Psychiatry* 171: 829 – 843

Pisu MG, Dore R, Mostallino MC, Loi M, Pibiri F, Mameli R, Cadeddu R, Secci PP, Serra M (2011) Down-regulation of hippocampal BDNF and arc associated with improvement in aversive spatial memory performance in socially isolated rats. *Behav Brain Res* 222: 73 – 80

Redmond L, Ghosh A (2005) Regulation of dendritic development by calcium signaling. *Cell Calcium* 37: 411 – 416

Redmond L, Kashani AH, Ghosh A (2002) Calcium regulation of dendritic growth via CaM kinase IV and CREB-mediated transcription. *Neuron* 34: 999 – 1010

van Rooij E, Quiat D, Johnson BA, Sutherland LB, Qi X, Richardson JA, Kelm RJ, Olson EN (2009) A family of microRNAs encoded by myosin genes governs myosin expression and muscle performance. *Dev Cell* 17: 662 – 673

Schratt G (2009) microRNAs at the synapse. *Nat Rev Neurosci* 10: 842 – 849

Schratt GM, Tuebing F, Nigh EA, Kane CG, Sabatini ME, Kiebler M, Greenberg ME (2006) A brain-specific microRNA regulates dendritic spine development. *Nature* 439: 283 – 289

Seffer D, Rippberger H, Schwarting RKW, Wôhr M (2015) Pro-social 50-kHz ultrasonic communication in rats: post-weaning but not post-adolescent social isolation leads to social impairments—phenotypic rescue by re-socialization. *Front Behav Neurosci* 9: 1 – 14

Smalheiser NR, Lugli G, Zhang H, Rizavi H, Cook EH, Dwivedi Y (2014) Expression of microRNAs and other small RNAs in prefrontal cortex in schizophrenia, bipolar disorder and depressed subjects. *PLoS One* 9: e86469

Smedler E, Louhivuori L, Romanov RA, Masini D, Dehnisch Ellstrom I, Wang C, Caramia M, West Z, Zhang S, Rebellato P *et al* (2022) Disrupted Cacna1c gene expression perturbs spontaneous ca^{2+} activity causing abnormal brain development and increased anxiety. *Proc Natl Acad Sci USA* 119: e2108768119

Solé B, Jiménez E, Torrent C, Reinares M, Bonnin CM, Torres I, Varo C, Grande I, Valls E, Salagre E *et al* (2017) Cognitive impairment in bipolar disorder: treatment and prevention strategies. *Int J Neuropsychopharmacol* 20: 670 – 680

Temme SJ, Murphy GG (2017) The L-type voltage-gated calcium channel Ca V 1.2 mediates fear extinction and modulates synaptic tone in the lateral amygdala. *Learn Mem* 24: 580 – 588

Tian Y, Voineagu I, Paşca SP, Won H, Chandran V, Horvath S, Dolmetsch RE, Geschwind DH (2014) Alteration in basal and depolarization induced transcriptional network in iPSC derived neurons from Timothy syndrome. *Genome Med* 6: 75

Toda T, Parylak SL, Linker SB, Gage FH (2019) The role of adult hippocampal neurogenesis in brain health and disease. *Mol Psychiatry* 24: 67 – 87

Tohka J, Zijdenbos A, Evans A (2004) Fast and robust parameter estimation for statistical partial volume models in brain MRI. *Neuroimage* 23: 84 – 97

Tsumagari K, Baribault C, Terragni J, Varley KE, Gertz J, Pradhan S, Badoo M, Crain CM, Song L, Crawford GE *et al* (2013) Early de novo DNA methylation and prolonged demethylation in the muscle lineage. *Epigenetics* 8: 317 – 332

Valluy J, Bicker S, Aksoy-Aksel A, Lackinger M, Sumer S, Fiore R, Wust T, Seffer D, Metge F, Dieterich C *et al* (2015) A coding-independent function of an alternative Ube3a transcript during neuronal development. *Nat Neurosci* 18: 666 – 673

Vlad M, Raucher-Chene D, Henry A, Kaladjian A (2018) Functional outcome and social cognition in bipolar disorder: is there a connection? *Eur Psychiatry* 52: 116 – 125

Vogelbacher C, Mobius TWD, Sommer J, Schuster V, Dannlowski U, Kircher T, Dempfle A, Jansen A, Bopp MHA (2018) The Marburg-Munster affective disorders cohort study (MACS): a quality assurance protocol for MR neuroimaging data. *Neuroimage* 172: 450 – 460

Walker EA, Gelfand A, Katon WJ, Koss MP, Von Korff M, Bernstein D, Russo J (1999) Adult health status of women with histories of childhood abuse and neglect. *Am J Med* 107: 332 – 339

Wang S, Stanika RI, Wang X, Hagen J, Kennedy MB, Obermair GJ, Colbran RJ, Lee A (2017) Densin-180 controls the trafficking and signaling of L-type voltage-gated ca v 1.2 ca^{2+} channels at excitatory synapses. *J Neurosci* 37: 4679 – 4691

© 2022 The Authors Published under the terms of the CC BY 4.0 license

Wang X, Luo Q, Tian F, Cheng B, Qiu L, Wang S, He M, Wang H, Duan M, Jia Z (2019) Brain grey-matter volume alteration in adult patients with bipolar disorder under different conditions: a voxel-based meta-analysis. *J Psychiatry Neurosci* 44: 89–101

White JA, McKinney BC, John MC, Powers PA, Kamp TJ, Murphy GG (2008) Conditional forebrain deletion of the L-type calcium channel CaV1.2 disrupts remote spatial memories in mice. *Learn Mem* 15: 1–5

WHO (1993) *The ICD-10 classification of mental and Behavioural disorders: diagnostic criteria for research (DCR-10)*. Geneva: World Health Organization

Wittchen H-U, Wunderlich U, Gruschwitz S, Zaudig M (1997) SKID I. Strukturiertes Klinisches Interview für DSM-IV. Achse I: Psychische Störungen. Interviewheft und Beurteilungsheft. Eine deutschsprachige, erweiterte Bearb. d. amerikanischen Originalversion des SKID I. Göttingen: Hogrefe. eDoc: 396152

Xu Y, Liu H, Li F, Sun N, Ren Y, Liu Z, Cao X, Wang Y, Liu P, Zhang K (2010) A polymorphism in the microRNA-30e precursor associated with major depressive disorder risk and P300 waveform. *J Affect Disord* 127: 332–336

Yoshimizu T, Pan JQ, Mungenast AE, Madison JM, Su S, Ketterman J, Ongur D, McPhie D, Cohen B, Perlis R *et al* (2015) Functional implications of a psychiatric risk variant within CACNA1C in induced human neurons. *Mol Psychiatry* 20: 162–169

Zenisek R, Thaler NS, Sutton GP, Ringdahl EN, Snyder JS, Allen DN (2015) Auditory processing deficits in bipolar disorder with and without a history of psychotic features. *Bipolar Disord* 17: 769–780

Zhou R, Yuan P, Wang Y, Hunsberger JG, Elkahloun A, Wei Y, Damschroder-Williams P, Du J, Chen G, Manji HK (2009) Evidence for selective microRNAs and their effectors as common long-term targets for the actions of mood stabilizers. *Neuropsychopharmacology* 34: 1395–1405

Zovoilis A, Agbemenyah HY, Agis-Balboa RC, Stilling RM, Edbauer D, Rao P, Farinelli L, Delalle I, Schmitt A, Falkai P *et al* (2011) microRNA-34c is a novel target to treat dementias. *EMBO J* 30: 4299–4308

License: This is an open access article under the terms of the Creative Commons Attribution License, which permits use, distribution and reproduction in any medium, provided the original work is properly cited.

© 2022 The Authors Published under the terms of the CC BY 4.0 license

Expanded View Figures

Figure EV1. Gene expression analysis in the JSI rat model and primary rat hippocampal neurons.

A Schematic representation of juvenile social isolation (JSI) experiment.

B–E qPCR analysis for c-fos (B), arc (C), miR-146b (D), and miR-30e-5p (E) using total RNA isolated from the hippocampus of male rats that were either group-housed or socially isolated for 4 weeks postweaning (n = 9 rats per group). Data are represented as box plot with whiskers (+: mean, line: median; whiskers: Tukey) (*P = 0.0207 (c-fos), *P = 0.0207 (arc); ***P = 0.0006 (miR-146-5p); P = 0.1049 (miR-30e-5p); Mann–Whitney U-test). Fold changes represent changes in gene expression relative to the control condition. U6 snRNA was used for normalization. ns = not significant.

F Relative expression of miR-499-5p in primary hippocampal neurons at different *DIVs* (n = 3 independent experiments). Total RNA was obtained from nontreated developing hippocampal neurons or treated from DIV 3 with FUDR to stop the proliferation of nonneuronal glial cells at the six indicated time points. Fold changes represent changes in miR-499-5p expression relative to DIV 4. Data are represented on XY graph as mean ± SD.

G Relative expression of miR-499-5p is significantly induced in hippocampal neurons treated with DEX compared to DMSO-treated neurons (n = 4; Paired two-sample t-test, *P = 0.0462). Data are represented as scattered dot plots with bar, mean ± SD. Fold change represents changes in miR-499-5p expression of DEX-treated neurons relative to DMSO-treated neurons.

H Relative expression of miR-499-5p is significantly induced in hippocampal neurons transfected with miR-499-5p mimics (n = 3 independent experiments; Ratio paired t-test, ***P = 0.003). Data are represented as scattered dot plots with bar, mean ± SD. Fold change represents changes in miR-499-5p expression of miR-499-5p mimic transfected-neurons relative to control mimic-transfected-neurons.

I Mean of the Sholl profile averages from biological replicates of Fig 1D and E. Data are represented on XY graph as mean ± SD. *P < 0.05.

© 2022 The Authors Published under the terms of the CC BY 4.0 license

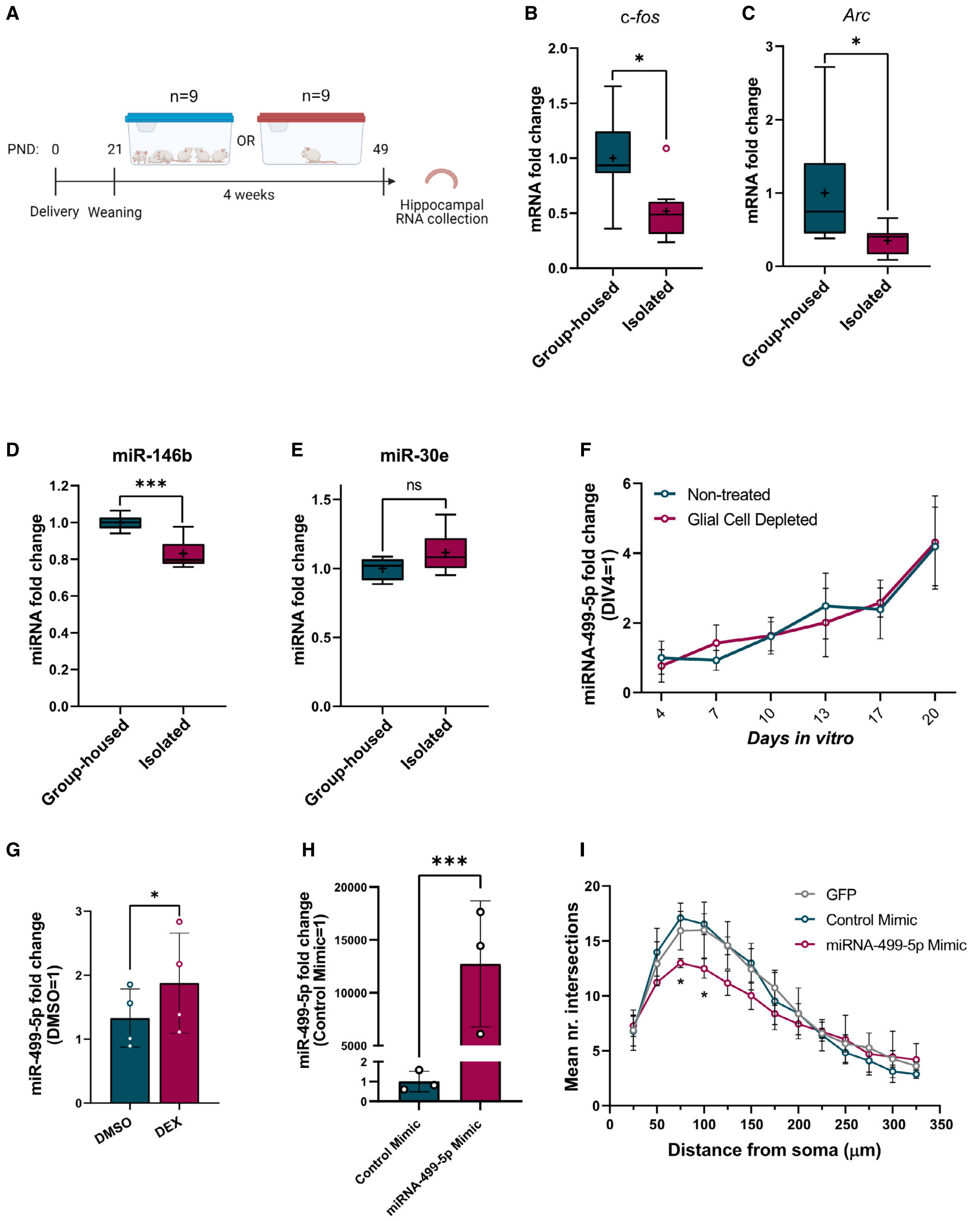

Figure EV1.

 © 2022 The Authors Published under the terms of the CC BY 4.0 license

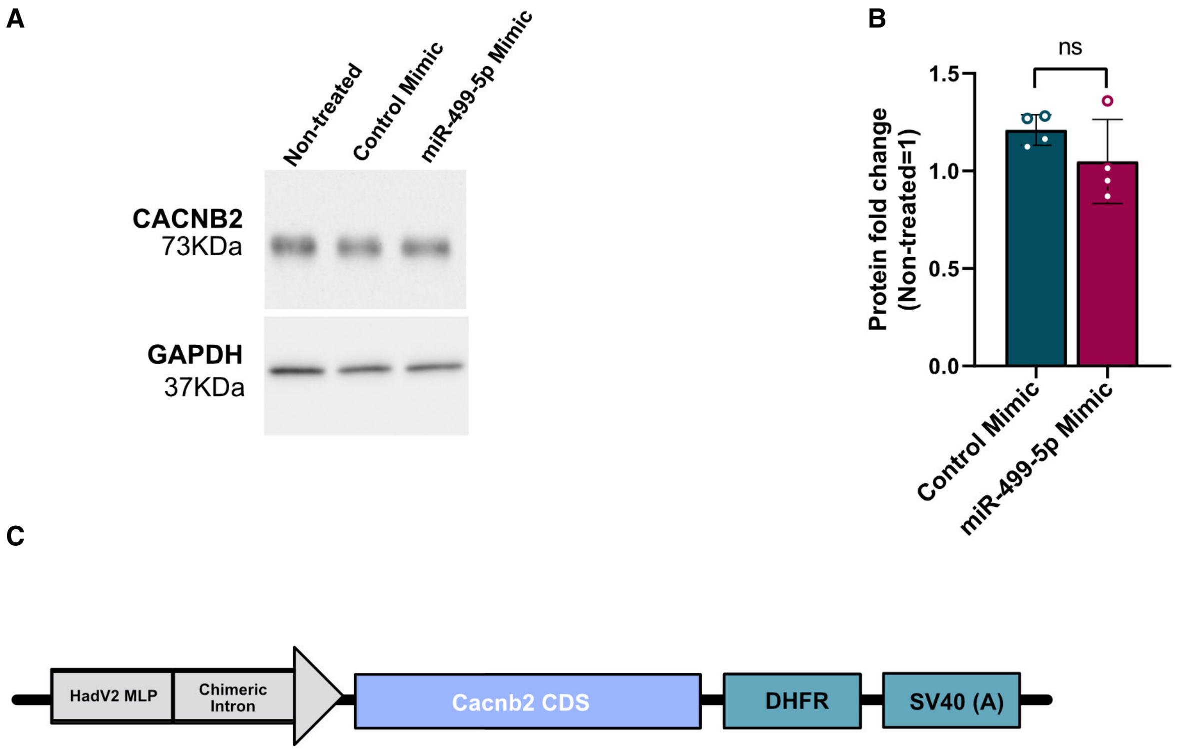

Figure EV2. CACNB2 protein expression upon miR-499-5p overexpression in rat hippocampal neurons.

A Representative western blot image of CACNB2 (upper panel) and GAPDH (lower panel) protein expression levels in hippocampal neurons (DIV14) that were transfected with the miR-499-5p mimic or with the control mimic at DIV 7. GAPDH was used as a loading control.

B Western blot analysis showed that miR-499-5p overexpression did not significantly change the CACNB2 expression compared to neurons expressing the control mimic (n = 4 independent experiments; Unpaired two-sample t-test, P = 0.2094). Data are represented as scattered dot plots with bar, mean ± SD.

C Schematic illustration of the pMT2-Cacnb2 overexpressing construct (hadV2 MLP: HadV2 Major Late Promoter; Cacnb2 CDS: Cacnb2 Coding Sequence; DHFR: mouse Dihydrofolate reductase).

Figure EV3. Quantification of $Ca_v1.2$ cell surface expression and channel properties upon miR-499-5p overexpression in hippocampal neurons.

A, B Quantification of the total levels of Cav1.2 channels of live-stained neurons transfected as in 3A. Neurons transfected with miR-499-5p mimic showed significant reductions in (A) the integrated density of surface Cav1.2-HA (n = 4; Paired two-sample t-test *P = 0.0385) and (B) the area of surface Cav1.2-HA (n = 4 independent experiments; Paired two-sample t-test *P = 0.0381). Data are represented as scattered dot plots with bar, mean ± SD.

C Representative images of *DIV* 19 rat hippocampal neurons co-transfected with GFP (green channel) and Cav1.2-HA (red channel), together with either control or miR-499-5p mimics. After 12–13 days of expression, labeling with Anti-HA antibodies was performed under permeabilized conditions to identify total levels of Cav1.2 channels. Scale bars = 20 μm.

D–F Quantification of the total levels of Cav1.2 channels of permeabilized neurons transfected as in Fig EV3C. No significant changes were found for (D) the total levels of Cav1.2 (n = 4 independent experiments; Paired two-sample t-test, P = 0.9776), (E) the integrated density (n = 4 independent experiments; Paired two-sample t-test, P = 0.3213), (F) the area (n = 4 independent experiments; Paired two-sample t-test, P = 0.3182) of total Cav1.2 channels. Data are represented as scattered dot plots with bar, mean ± SD.

G I/V curves before and after bath application with the LVGCC blocker Nifedipine (20 μM) (n = 3 cells nontreated vs. n = 3 cells treated with 20 μM Nifedipine). Data are represented on XY graph as mean ± SEM.

H Hippocampal neurons transfected with the miR-499-5p mimic did not show a different activation curve compared to the activation curves of cells transfected with the Control mimic or GFP alone (GFP: n = 9; NC mimic: n = 10, miR-499-5p mimic: n = 10). Data are represented on XY graph as mean ± SEM.

I Hippocampal neurons overexpressing miR-499-5p did not show a different inactivation curve compared to the inactivation curves of control cells (GFP: n = 9; NC mimic: n = 10, miR-499-5p mimic: n = 10). Data are represented on XY graph as mean ± SEM.

J MiR-499-5p overexpression tended to decrease capacitance (GFP: n = 9; NC mimic: n = 10, miR-499-5p mimic: n = 10; Unpaired two-sample t-test, P = 0.0698). Data are represented as box plot with whiskers and data points (line: median; whiskers: minimum and maximum values).

▶

© 2022 The Authors Published under the terms of the CC BY 4.0 license

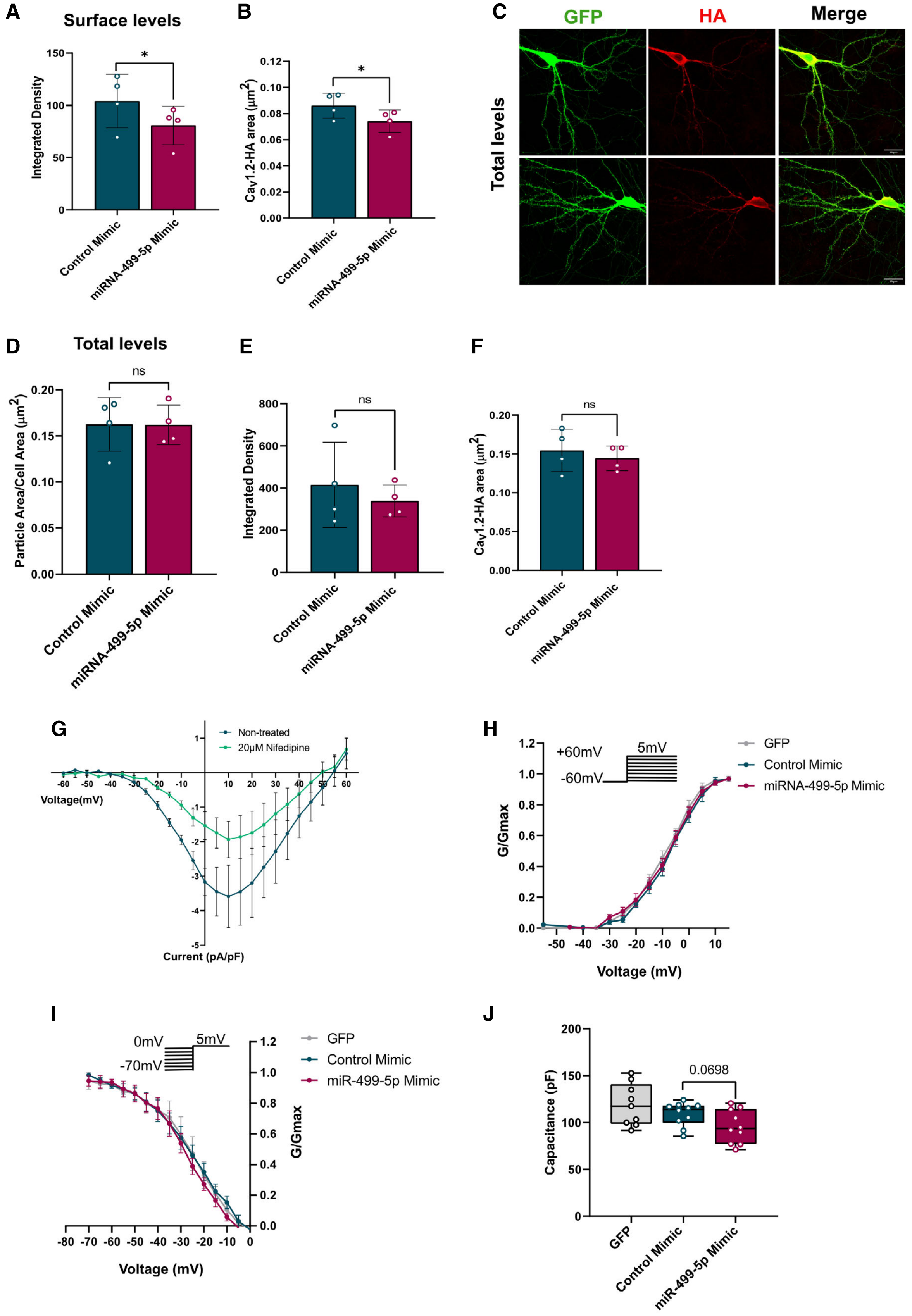

Figure EV3.

 © 2022 The Authors Published under the terms of the CC BY 4.0 license

Figure EV4. Additional biochemical and behavioral characterization of WT and Cacna1c$^{+/-}$ rats overexpressing miR-499-5p.

A, B Significant negative correlation between relative miR-499-5p expression and *CACNB2* protein levels in the hippocampus of WT (A) and Cacna1c$^{+/-}$ rats (B) injected with AAV-Control (red) and AAV-miR-499 (blue). Spearman correlation coefficient with two-tailed analysis is provided in the legend.

C Object acquisition task. Total time (seconds) WT or *Cacna1c*$^{+/-}$ rats injected with the indicated AAV spent exploring the objects. Data are presented as box and whisker plot with median, mean, and minimum and maximum values. ns: not significant, one-way ANOVA; WT_Control: n = 18 (eight males, 10 females); WT_miR-499: n = 16 (seven males, nine females); *Cacna1c*$^{+/-}$_Control: n = 15 (five males, 10 females); *Cacna1c*$^{+/-}$_miR-499: n = 16 (10 males, six females). Main effect Genotype: $F_{1,61}$ = 0.501, P = 0.482; main effect Treatment: $F_{1,61}$ = 0.929, P = 0.339; Interaction Genotype × Treatment: $F_{1,61}$ = 0.098, P = 0.756.

D Total arms entries during Elevated Plus Maze test did not differ between genotypes and treatment groups (as in Fig 4H). Data are presented as box and whisker plot with median, mean, and minimum and maximum values. Ns: not significant, one-way ANOVA. Main effect Genotype: $F_{1,64}$ = 0.257, P = 0.614, main effect Treatment: $F_{1,64}$ = 0.007, P = 0.934, interaction Genotype × Treatment: $F_{1,64}$ = 0.005, P = 0.946.

E Open field test. Total distance traveled (cm) by WT or *Cacna1c*$^{+/-}$ rats injected with the indicated AAV over a time course of 10 min on 2 consecutive days. Data are presented as means ± SD. Repeated measures ANOVA; WT_Control: n = 19 (nine males, 10 females); WT_miR-499: n = 16 (seven males, nine females); *Cacna1c*$^{+/-}$_Control: n = 17 (seven males, 10 females); *Cacna1c*$^{+/-}$_miR-499: n = 16 (10 males, six females). Main effect Genotype: P = 0.138; main effect Treatment: P = 0.672; interaction Genotype × Treatment: P = 0.124.

© 2022 The Authors Published under the terms of the CC BY 4.0 license

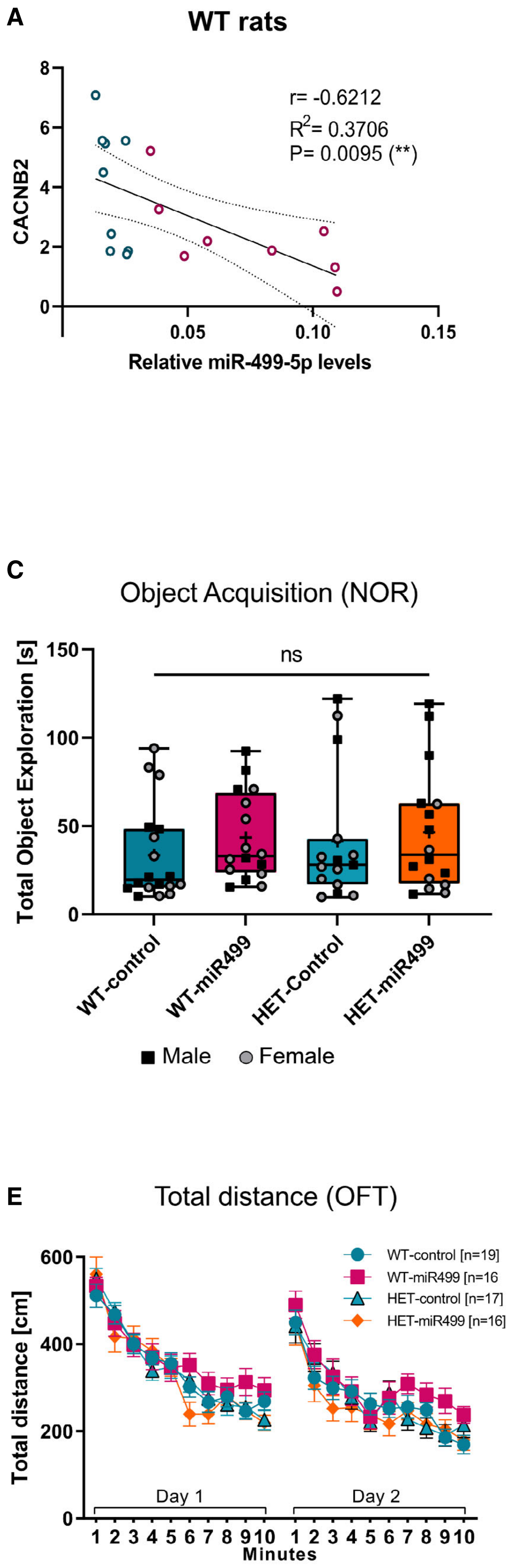

Figure EV4.

 © 2022 The Authors Published under the terms of the CC BY 4.0 license

Figure EV5. Additional analysis of miR-499-5p expression in different healthy control and patient subgroups.

A Schematic illustration of the experimental workflow. Total RNA was isolated from PBMCs of psychiatrically healthy subjects (Controls or maltreated), BD and MDD patients for miRNA expression analysis.

B miR-499-5p qPCR analysis of total RNA isolated from PBMCs of control (female = 26, male = 31), BD subjects (female = 19, male = 21), or BD subjects under Antidepressant (AD) treatment (female = 7, male = 16) subjects. Two-way ANOVA, Main effect Sex: $P = 0.378$; main effect Group: ****$P = 1.68e-11$; Interaction group × sex: $P = 0.663$. Tukey's HSD: control versus BD and control versus BD + AD: ****$P < 0.00001$; BD versus BD + AD *$P = 0.02526$. Data are presented as violin plots with median, quartiles and data points.

C miR-499-5p qPCR analysis of total RNA isolated from PBMCs of control (female = 26, male = 31), MDD subjects (female = 1, male = 9), or MDD subjects under Antidepressant (AD) treatment (female = 15, male = 16) subjects. Two-way ANOVA, Main effect Sex: **$P = 0.003247$; main effect Group: *$P = 0.010018$; Interaction group × sex: ***$P = 0.000297$. Tukey's HSD: control versus MDD: $P = 0.5190$; control versus MDD + AD: *$P = 0.0306$; MDD versus MDD + AD: *$P = 0.0274$. Data are presented as violin plots with median, quartiles, and data points.

D miR-499-5p qPCR analysis of total RNA isolated from PBMCs of control −CMT (female = 10, male = 16), control +CMT (female = 11, male = 9), BD −CMT (female = 18, male = 24), and BD + CMT (female = 8, male = 13). Three-way ANOVA, main effect Sex: $P = 0.4991$, main effect CMT: $P = 0.5434$; main effect Group: $P < 0.0001$; interaction CMT × Group: $P = 0.7532$; interaction CMT × Sex: $P = 0.3259$; interaction Group × Sex: $P = 0.3259$; interaction CMT × Group × Sex: $P = 0.9703$. Tukey's HSD: control CMT− versus BD CMT−: ****$P < 0.0001$; control CMT+ versus BD CMT+: **$P = 0.0013$. Data are presented as violin plots with median, quartiles, and data points.

E, F miR-499-5p expression does not correlate with Young Mania Rating Scale or Beck's Depression Inventory scores from BD patients. Spearman correlation coefficient with two-tailed analysis is provided in the legend. Squares: males, circles: females.

© 2022 The Authors Published under the terms of the CC BY 4.0 license

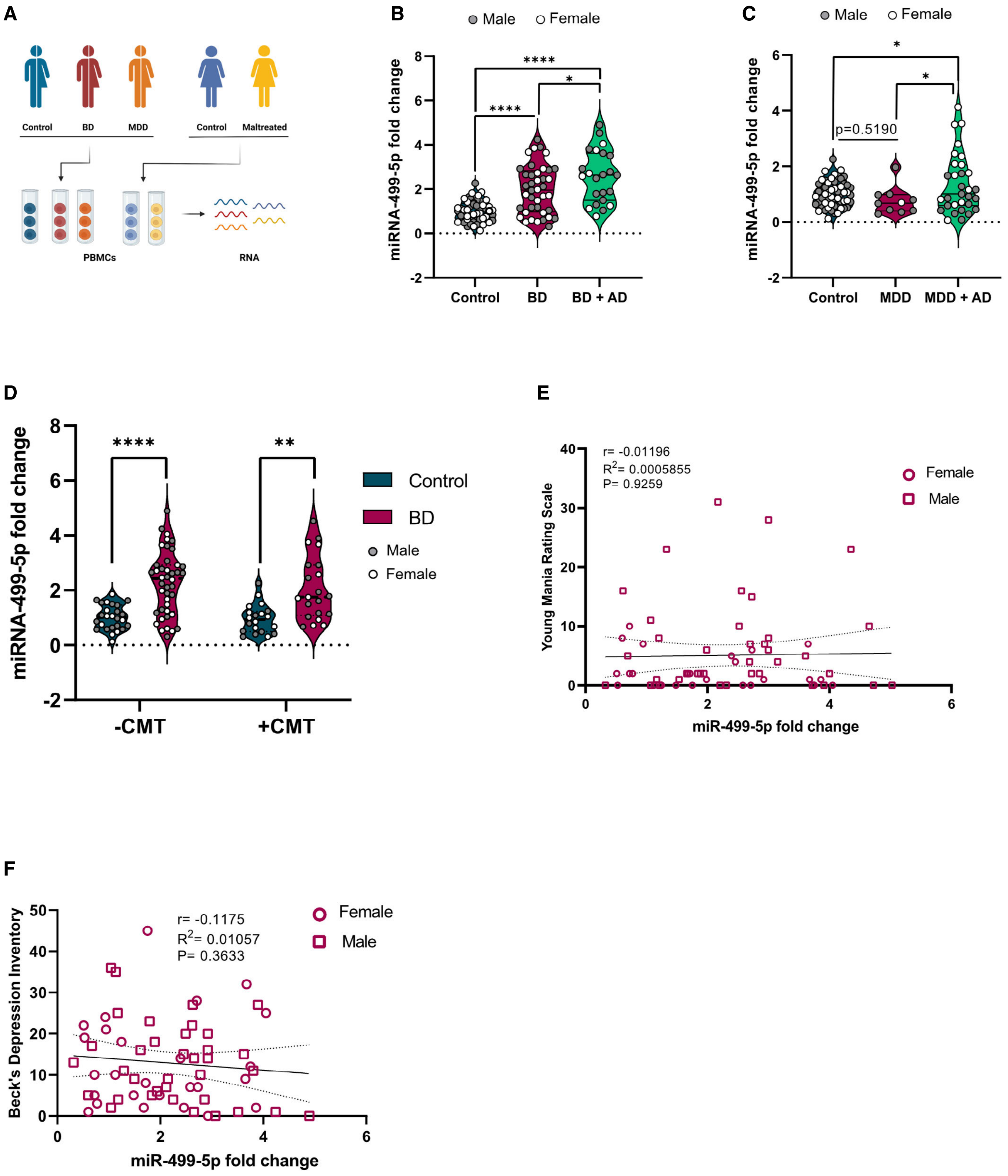

Figure EV5.

 © 2022 The Authors Published under the terms of the CC BY 4.0 license

Table of Contents

Appendix Figure S1. Validation of rAAV expression via GFP in rat hippocampus 2

Appendix Figure S2. miR-499-5p expression in WT and *Cacna1c*$^{+/-}$ in rat hippocampus upon rAAV injection 3

Appendix Figure S3. Correlation analyses between peripheral miR-499-5p expression and CTQ, BDI and HAMD scores 4

Appendix Figure S4. miR-499-5p expression in human female and male subjects 6

Appendix Table S1a and S1b. Clinical data of human patients (Control, BD, and MDD) 9

Appendix Table S2. Bipolar Disorder subtypes and mood states of BD patients 11

Appendix Table S3. Clinical data of human healthy controls 11

Appendix Figure S1

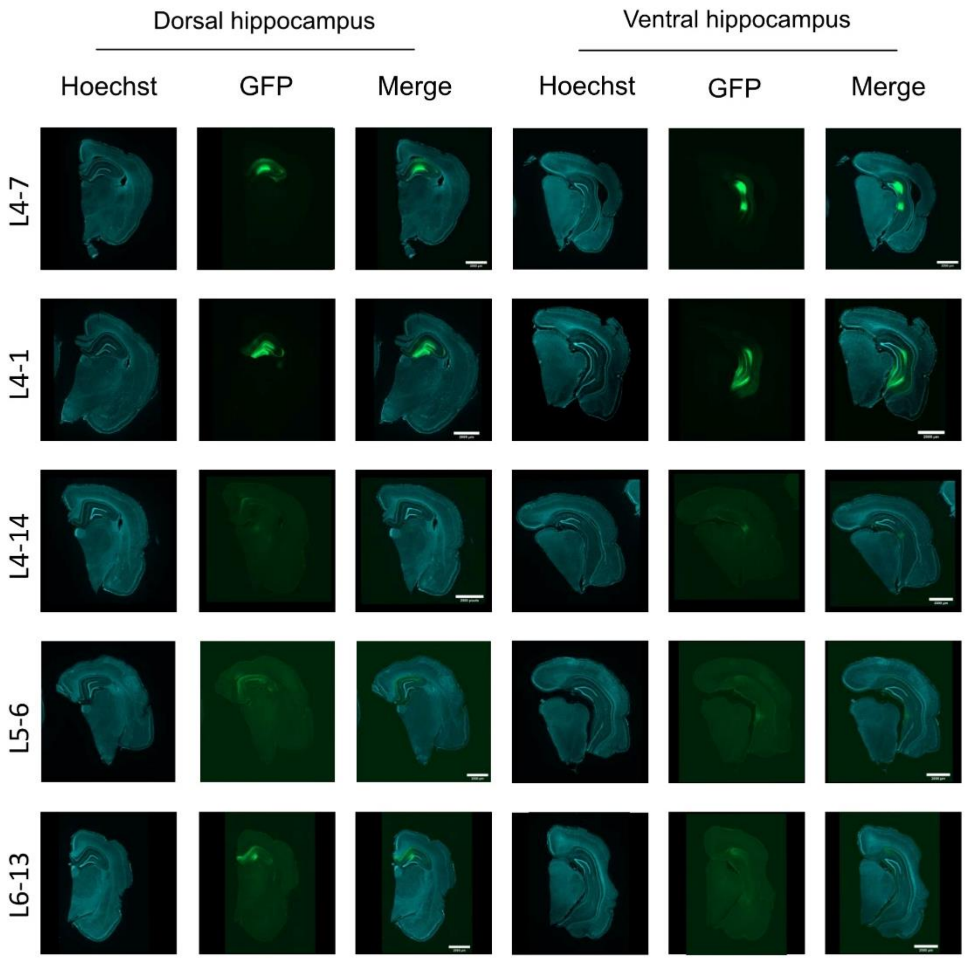

Validation of hippocampal rAAV expression by the presence of GFP fluorescence in the left hemisphere of rats expressing chimeric hairpins. Left hemispheres were dissected from rats injected bilaterally in the dorsal and ventral hippocampus with either miRNA-499 hairpin or control hairpin. Coronal brain sections of 80 µm were stained with Hoechst. Green fluorescence represents the GFP native signal of the chimeric hairpin; L4-7, L4-1, L4-14, L5-6 and L6-13: left hemisphere of rats injected with the chimeric miR-499 hairpin showing strong GFP fluorescence in both dorsal and ventral hippocampi. Scale bar: 2000 µm.

Appendix Figure S2

A-D) Fold expression of miR-499-5p in WT (A and C) and *Cacna1c*$^{+/-}$ (B and D) rats relative to control sequence expression from chimeric hairpins (blue: AAV-control; green: AAV-miR-499.Red arrows highlight animals that were excluded from behavioral analysis since miR-499-5p expression was lower than the average miR-499-5p expression of animals injected with the control hairpin + 2 S.D.

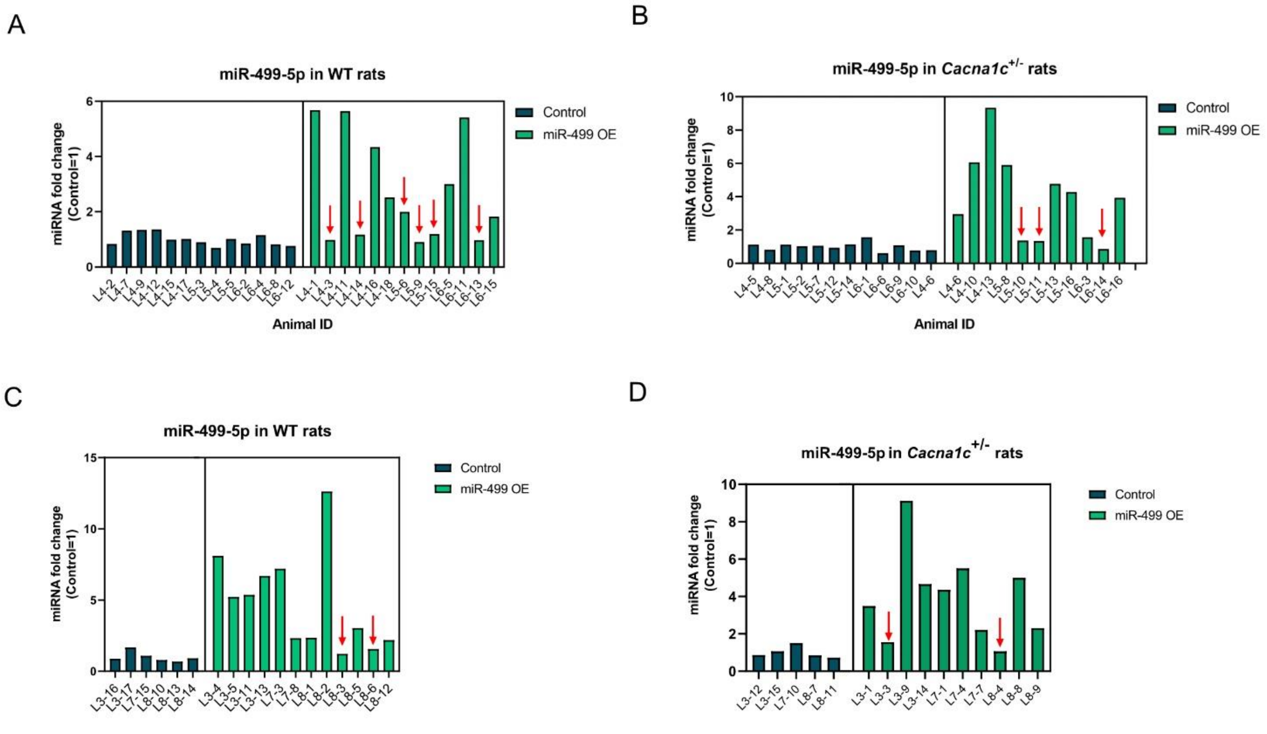

Appendix Figure S3

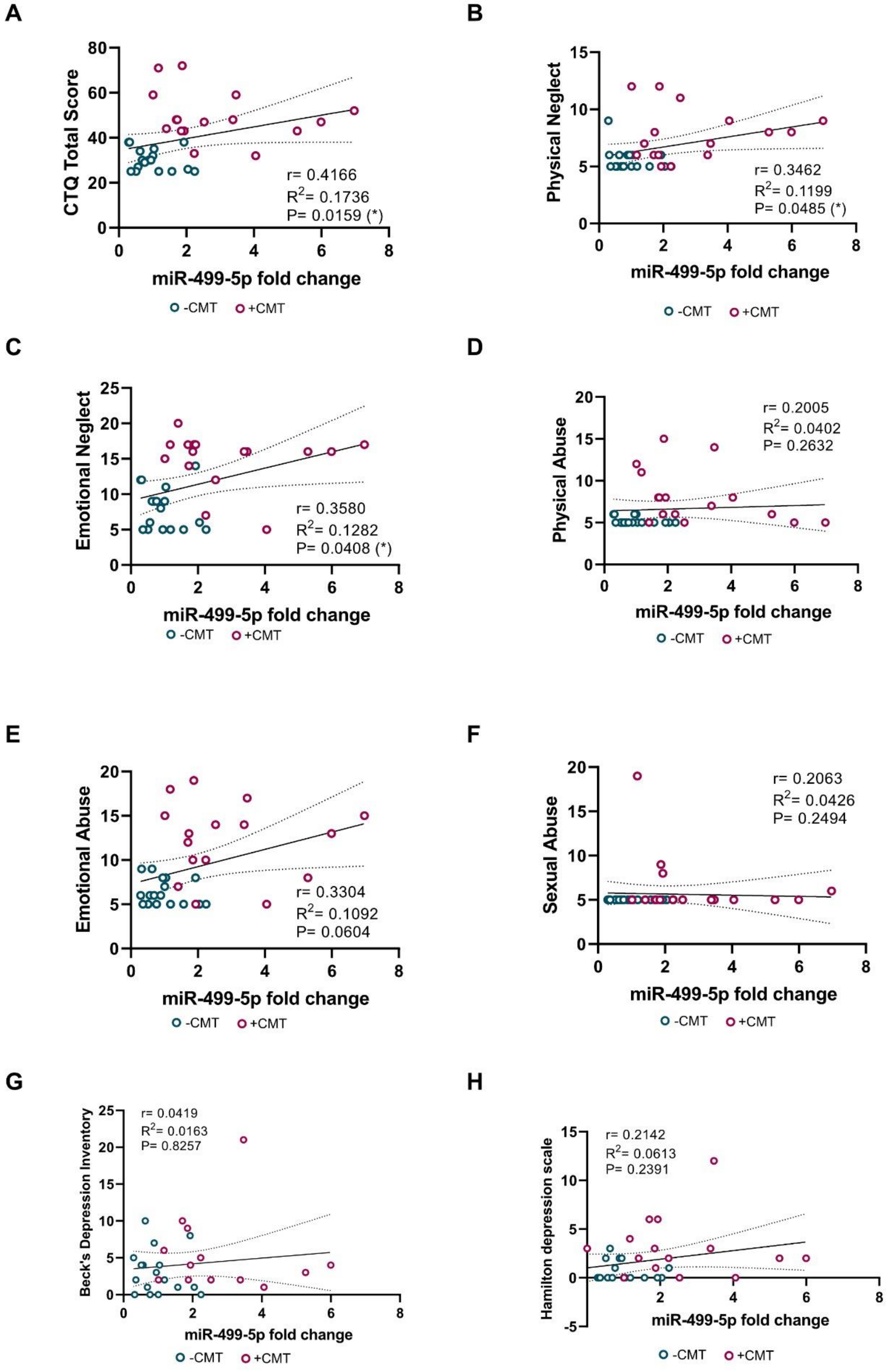

A-F) Positive correlation between the peripheral levels of miR-499-5p in control (-CMT) (blue) or maltreated (+CMT) (red) healthy individuals and A) the sum of all subscale CTQ scores (CTQ Total Score), B) the scores for physical neglect, and C) the scores for emotional neglect. No significant correlation was found between the peripheral levels of miR-499-5p and the scores for D) physical abuse, E) emotional abuse and F) sexual abuse. Spearman correlation coefficient with two-tailed analysis. Data are presented as XY tables.

G-H) No correlation between the peripheral levels of miR-499-5p in control (-CMT) (blue) or maltreated (+CMT) (red) healthy individuals and G) Becks's depression Inventory (BDI) and H) Hamilton depression scale (HAMD) scores. Spearman correlation coefficient with two-tailed analysis. Data are presented as XY tables.

Appendix Figure S4

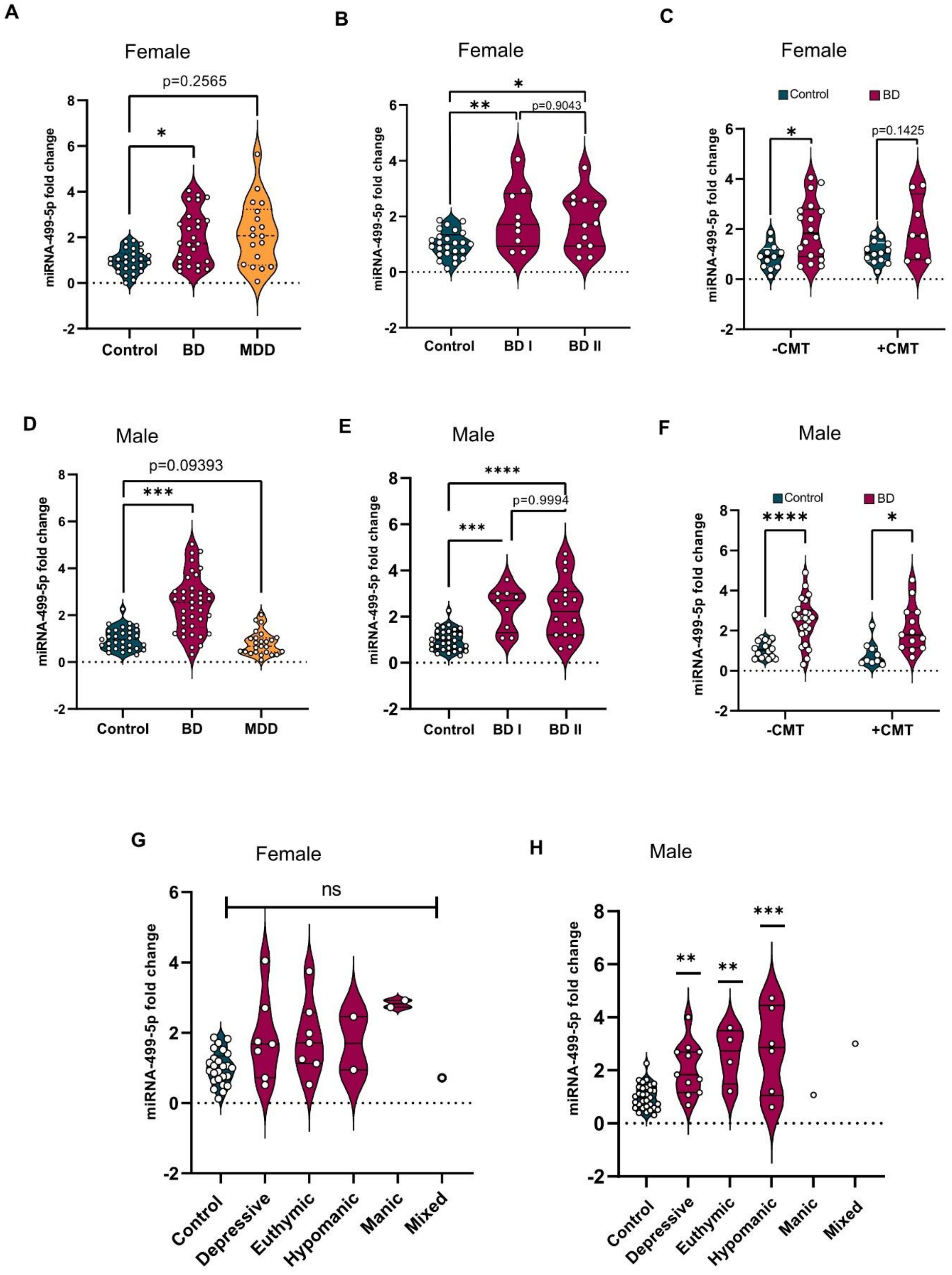

A) miR-499-5p qPCR analysis of total RNA isolated from PBMCs of female subjects (control, n= 26; BD, n=26; MDD, n=18). Peripheral miR-499-5p is significantly up-regulated in BD patients (**p=0.00824, Wilcoxon rank-sum test) but not in MDD patients (p=0.11457, Wilcoxon rank-sum test) compared to control subjects after correction for age and antidepressant treatment (linear model of the form FC ~ Group + Age + Antidepressant treatment). Data are presented as violin plots with median, quartiles and data points.

B) miR-499-5p qPCR analysis of total RNA isolated from PBMCs of female subjects (control, n=26; BD I, n=8; BD II, n=12). One-way ANOVA, Post hoc Tukey test: Control vs BD I: **p=0.0099, Control vs BD II: *p=0.0170, BD I and BD II p=0.9043). Data are presented as violin plots with median, quartiles and data points.

C) miR-499-5p qPCR analysis of total RNA isolated from PBMCs of female subjects (control -CMT: n=10; control +CMT: n= 11; BD -CMT: n=18; BD +CMT: n=8). Two-way ANOVA, Post-hoc Tukey Test. Main effect CMT: p= 0.74151, main effect Group: ***p=0.0006. Control -CMT vs BD -CMT: *p=0.0146; Control +CMT vs BD +CMT: ns, p=0.1425. Data are presented as violin plots with median, quartiles and data points.

D) miR-499-5p qPCR analysis of total RNA isolated from PBMCs male subjects (control, n= 31; BD, n=37; MDD, n=24). Peripheral miR-499-5p is significantly up-regulated in BD patients (****p= 4.38e-07, Wilcoxon rank-sum test) but not in MDD patients (p= 0.09393, Wilcoxon rank-sum test) compared to control subjects after correction for age and antidepressant treatment (linear model of the form delta FC~ Group + Age + Antidepressant treatment). Data are presented as violin plots with median, quartiles and data points.

E) miR-499-5p qPCR analysis of total RNA isolated from PBMCs of male subjects (control, n=31; BDI, n= 9; BD II, n=16). One-way ANOVA, Post hoc Tukey test: Control vs BD I: ***p=0.0003, Control vs BD II: ****p=0.00001, BD I and BD II p=0.9994). Data are presented as violin plots with median, quartiles and data points.

F) miR-499-5p qPCR analysis of total RNA isolated from PBMCs of male subjects (control -CMT: n=16; control +CMT: n= 9; BD -CMT: n=24; BD +CMT: n=13). Two-way ANOVA, Post-hoc Tukey Test. Main effect CMT: p= 0.2593, main effect Group: ****p= 4.01e-07, interaction CMT x Group: ns, p=0.790. Control -CMT vs BD -CMT: ****p=0.00010; Control +CMT vs BD +CMT: *, p= 0.0120. Data are presented as violin plots with median, quartiles and data points.

G) miR-499-5p qPCR analysis of total RNA isolated from PBMCs of female subjects (control, n= 26) and BD subjects in different mood states (depressive, n=7; euthymic, n= 7; hypomanic, n=2; manic, n=2; mixed, n=1). One-way ANOVA, Post hoc Tukey test. Data are presented as violin plots with median, quartiles and data points.

H) miR-499-5p qPCR analysis of total RNA isolated from PBMCs of male subjects (control, n= 31) and BD subjects in different mood states (depressive, n=11; euthymic, n= 4; hypomanic, n=6; manic, n=1; mixed, n=1). One-way ANOVA, Post hoc Tukey test: Control vs. Depressive, **p=0.0071; Control vs. Euthymic, **p=0.0098; Control vs. Hypomanic, ***p=0.0002. Data are presented as violin plots with median, quartiles and data points.

Appendix Table S1A and S1B

Subjects used for miR-499-5p expression analysis on psychiatrically healthy controls (Control), Bipolar disorder patients (BD) or Major Depressive Disorder patients (MDD). One-way-ANOVA was performed to evaluate significant differences between groups. S.D.: standard deviation, CTQ: Childhood Maltreatment Questionnaire, HAMD: Hamilton Depression Rating Scale, YMRS: Young Mania Rating Scale, BDI: Beck's Depression Inventory, AD: antidepressant use.

Table S1A	Control	BD	MDD	P value
**n**	26	26	18	N/A
**Sex**	Female	Female	Female	N/A
**Age ± S.D.**	29.4 ± 5.4	29.9 ± 5.4	29.6± 4.9	0.94
**CTQ ± S.D. (%)**	40.7 ± 11.4 (53.8%)	42.3 ± 13.2 (30.8%)	50.2 ± 20.2 (61%)	0.1067
**Family history of Affective Disorders (%)**	8 (30.8%)	10 (38.5%)	5 (27.8%)	N/A
**HAMD ±S.D.**	2.7 ± 3.9	7.5 ± 6.2	14.8 ± 6.7	<0.0001
**YMRS ± S.D.**	0.5 ± 1.1	2.4 ± 2.9	1.4 ± 1.6	<0.01
**BDI ± S.D.**	6.1 ± 6.1	12.9 ± 11.1	26.6 ± 11.0	<0.0001
**AD (%)**	0 (0%)	7 (26.9%)	16 (88.9%)	N/A
**Antipsychotic (%)**	0 (0%)	12 (46.15%)	7 (38.89%)	N/A
**Lithium (%)**	0 (0%)	4 (15.38%)	1 (5.56%)	N/A
**Anticonvulsive (%)**	0 (0%)	6 (23.08%)	1 (5.56%)	N/A
**Stimulants (%)**	0 (0%)	0 (0%)	0 (0%)	N/A
**Benzodiazepine (%)**	0 (0%)	0 (0%)	1 (5.56%)	N/A
**Z substance (%)**	0 (0%)	0 (0%)	1 (5.56%)	N/A

Table S1B	Control	BD	MDD	P value
n	26	26	18	N/A
**Sex**	Female	Female	Female	N/A
**Age ± S.D.**	29.4 ± 5.4	29.9 ± 5.4	29.6± 4.9	0.94
**CTQ ± S.D. (%)**	40.7 ± 11.4 (53.8%)	42.3 ± 13.2 (30.8%)	50.2 ± 20.2 (61%)	0.1067
**Family history of Affective Disorders (%)**	8 (30.8%)	10 (38.5%)	5 (27.8%)	N/A
**HAMD ±S.D.**	2.7 ± 3.9	7.5 ± 6.2	14.8 ± 6.7	<0.0001
**YMRS ± S.D.**	0.5 ± 1.1	2.4 ± 2.9	1.4 ± 1.6	<0.01
**BDI ± S.D.**	6.1 ± 6.1	12.9 ± 11.1	26.6 ± 11.0	<0.0001
**AD (%)**	0 (0%)	7 (26.9%)	16 (88.9%)	N/A
**Antipsychotic (%)**	0 (0%)	12 (46.15%)	7 (38.89%)	N/A
**Lithium (%)**	0 (0%)	4 (15.38%)	1 (5.56%)	N/A
**Anticonvulsive (%)**	0 (0%)	6 (23.08%)	1 (5.56%)	N/A
**Stimulants (%)**	0 (0%)	0 (0%)	0 (0%)	N/A
**Benzodiazepine (%)**	0 (0%)	0 (0%)	1 (5.56%)	N/A
**Z substance (%)**	0 (0%)	0 (0%)	1 (5.56%)	N/A

Appendix Table S2

BD subtype (I/II/NA) and Mood state (d: depressive, e: euthymic, h: hypomanic, m: manic; mi: mixed) of the BD subjects used for miR-499-5p expression analysis; NA: information not available.

**Table S2**	**Female subjects**	**Male subjects**
**BD subtype (I/II/NA)**	9/12/5	11/16/10
**Mood state (d/e/h/m/mi/NA)**	7/7/2/2/1/7	11/4/6/1/1/14

Appendix Table S3

Subjects used for miR-499-5p expression analysis in healthy controls (Control) and subjects with a history of childhood maltreatment (Maltreated). Unpaired t-Test was performed to evaluate significant differences between groups. S.D.: standard deviation, CTQ: Childhood Maltreatment Questionnaire, AD: antidepressant use.

**Table S3**	**Control**	**Maltreated**	**P value**
n	18	17	N/A
**Sex**	Female	Female	N/A
**Age ± S.D.**	27.4 ± 7.2	32.3 ± 10.0	0.11
**CTQ ± S.D. (%)**	29.8 ± 4.9 (0%)	49.3 ± 10.9 (100%)	<0.0001
