# Supplementary material for: Bipolar‐associated miR‐499‐5p controls neuroplasticity by downregulating the Cav1.2 subunit CACNB2
Source: EMBO Rep. 2022 Aug 15;23(10):e54420. doi: 10.15252/embr.202154420 (PMC9535808; doi:10.15252/embr.202154420)
Supplement: Supplementary file 4 [file EMBR-23-e54420-s002.pdf]

EMBO reports

Bipolar-associated miR-499-5p controls neuroplasticity by downregulating the Cav1.2 subunit CACNB2

Helena Martins, Carlotta Gilardi, Özge Sungur, Jochen Winterer, Michael Pelzl, Silvia Bicker, Fridolin Gross, Theresa Kisko, Natalia Malikowska-Racia, Moria Braun, Katharina Brosch, Igor Nenadic, Frederike Stein, Susanne Meinert, Rainer Schwarting, Udo Dannlowski, Tilo Kircher, Markus Wöhr, and Gerhard Schratt
DOI: 10.15252/embr.202154420

Corresponding author(s): Gerhard Schratt (gerhard.schratt@hest.ethz.ch)

Review Timeline:

Submission Date:	1st Dec 21
Editorial Decision:	3rd Dec 21
Revision Received:	27th May 22
Editorial Decision:	7th Jul 22
Revision Received:	21st Jul 22
Accepted:	26th Jul 22

Editor: Esther Schnapp

Transaction Report:

Please note that the manuscript was previously reviewed at another journal outside EMBO press and the reports were taken into account in the decision making process at EMBO Reports. Since the original reviews are not subject to EMBO Press' transparent review process policy, the reports and author response cannot be published.

Dear Gerhard,

Thank you for the submission of your manuscript with referee reports and your proposed revision plan to EMBO reports.

I read all files and discussed it with the EMBO reports team, and we all agree that the manuscript will make a nice contribution to our scientific reports/article section. I think all referee reports are very good, and we agree with your proposed revision plan.

I would thus like to invite you to revise your manuscript as you propose for publication here. Please address all referee concerns in a complete point-by-point response. Acceptance of the manuscript will depend on a positive outcome of a second round of review. I will contact Mol Psychiatry and see if they are willing to share the referee identities with us, so that I can send your revised manuscript back to the same referees. If this is not possible, I will involve one advisor to assess your final manuscript.

It is EMBO reports policy to allow a single round of major revision only and acceptance or rejection of the manuscript will therefore depend on the completeness of your responses included in the next, final version of the manuscript.

Revised manuscripts should be submitted within three months of a request for revision; however, given that you have started the revisions already, we can may be agree on a submission in 1 or 2 months from now? Please let me know what you think.

You can either publish the study as a short report or as a full article. For short reports, the revised manuscript should not exceed 27,000 characters (including spaces but excluding materials & methods and references) and 5 main plus 5 expanded view figures. The results and discussion sections must further be combined, which will help to shorten the manuscript text by eliminating some redundancy that is inevitable when discussing the same experiments twice. For a normal article there are no length limitations, but it should have more than 5 main figures and the results and discussion sections must be separate.

IMPORTANT NOTE: we perform an initial quality control of all revised manuscripts before re-review. Your manuscript will FAIL this control and the handling will be DELAYED if the following APPLIES:
1) A data availability section providing access to data deposited in public databases is missing. If you have not deposited any data, please add a sentence to the data availability section that explains that.
2) Your manuscript contains statistics and error bars based on n=2. Please use scatter blots in these cases. No statistics should be calculated if n=2.

When submitting your revised manuscript, please carefully review the instructions that follow below. Failure to include requested items will delay the evaluation of your revision.

1) a .docx formatted version of the manuscript text (including legends for main figures, EV figures and tables). Please make sure that the changes are highlighted to be clearly visible.

2) individual production quality figure files as .eps, .tif, .jpg (one file per figure). See https://wol-prod-cdn.literatumonline.com/pb-assets/embo-site/EMBOPress_Figure_Guidelines_061115-1561436025777.pdf for more info on how to prepare your figures.

3) We replaced Supplementary Information with Expanded View (EV) Figures and Tables that are collapsible/expandable online. A maximum of 5 EV Figures can be typeset. EV Figures should be cited as 'Figure EV1, Figure EV2" etc... in the text and their respective legends should be included in the main text after the legends of regular figures.

- For the figures that you do NOT wish to display as Expanded View figures, they should be bundled together with their legends in a single PDF file called *Appendix*, which should start with a short Table of Content. Appendix figures should be referred to in the main text as: "Appendix Figure S1, Appendix Figure S2" etc. See detailed instructions regarding expanded view here: <https://www.embopress.org/page/journal/14693178/authorguide#expandedview>

- Additional Tables/Datasets should be labeled and referred to as Table EV1, Dataset EV1, etc. Legends have to be provided in a separate tab in case of .xls files. Alternatively, the legend can be supplied as a separate text file (README) and zipped together with the Table/Dataset file.

4) a .docx formatted letter INCLUDING the reviewers' reports and your detailed point-by-point responses to their comments. As part of the EMBO Press transparent editorial process, the point-by-point response is part of the Review Process File (RPF), which will be published alongside your paper.

5) a complete author checklist, which you can download from our author guidelines <https://www.embopress.org/page/journal/14693178/authorguide>. Please insert information in the checklist that is also reflected in the manuscript. The completed author checklist will also be part of the RPF.

6) Please note that all corresponding authors are required to supply an ORCID ID for their name upon submission of a revised

manuscript (<https://orcid.org/>). Please find instructions on how to link your ORCID ID to your account in our manuscript tracking system in our Author guidelines <https://www.embopress.org/page/journal/14693178/authorguide#authorshipguidelines>

7) Before submitting your revision, primary datasets produced in this study need to be deposited in an appropriate public database (see https://www.embopress.org/page/journal/14693178/authorguide#datadeposition). Please remember to provide a reviewer password if the datasets are not yet public. The accession numbers and database should be listed in a formal "Data Availability" section placed after Materials & Method (see also https://www.embopress.org/page/journal/14693178/authorguide#datadeposition). Please note that the Data Availability Section is restricted to new primary data that are part of this study. * Note - All links should resolve to a page where the data can be accessed. *
If your study has not produced novel datasets, please mention this fact in the Data Availability Section.

8) We would also encourage you to include the source data for figure panels that show essential data. Numerical data should be provided as individual .xls or .csv files (including a tab describing the data). For blots or microscopy, uncropped images should be submitted (using a zip archive if multiple images need to be supplied for one panel). Additional information on source data and instruction on how to label the files are available at <https://www.embopress.org/page/journal/14693178/authorguide#sourcedata>.

9) Our journal also encourages inclusion of *data citations in the reference list* to directly cite datasets that were re-used and obtained from public databases. Data citations in the article text are distinct from normal bibliographical citations and should directly link to the database records from which the data can be accessed. In the main text, data citations are formatted as follows: "Data ref: Smith et al, 2001" or "Data ref: NCBI Sequence Read Archive PRJNA342805, 2017". In the Reference list, data citations must be labeled with "[DATASET]". A data reference must provide the database name, accession number/identifiers and a resolvable link to the landing page from which the data can be accessed at the end of the reference. Further instructions are available at https://www.embopress.org/page/journal/14693178/authorguide#referencesformat

10) Regarding data quantification (see Figure Legends: https://www.embopress.org/page/journal/14693178/authorguide#figureformat)

The following points must be specified in each figure legend:

- the name of the statistical test used to generate error bars and P values,

- the number (n) of independent experiments (please specify technical or biological replicates) underlying each data point,

- the nature of the bars and error bars (s.d., s.e.m.),

- If the data are obtained from n {less than or equal to} 2, use scatter blots showing the individual data points.

Discussion of statistical methodology can be reported in the materials and methods section, but figure legends should contain a basic description of n, P and the test applied.

- Please also include scale bars in all microscopy images.

We would also welcome the submission of cover suggestions, or motifs to be used by our Graphics Illustrator in designing a cover.

As part of the EMBO publication's Transparent Editorial Process, EMBO reports publishes online a Review Process File (RPF) to accompany accepted manuscripts. This File will be published in conjunction with your paper and will include the referee reports, your point-by-point response and all pertinent correspondence relating to the manuscript. Of course, in your case, we will have to obtain consent from the referees first.

You are able to opt out of this by letting the editorial office know (emboreports@embo.org). If you do opt out, the Review Process File link will point to the following statement: "No Review Process File is available with this article, as the authors have chosen not to make the review process public in this case."

I look forward to seeing a revised form of your manuscript when it is ready. Please use this link to submit your revision: https://embor.msubmit.net/cgi-bin/main.plex

Best regards,
Esther

Point-by-point response

We thank the reviewers for their overall extremely positive feedback and the constructive criticisms which helped to further improve our manuscript. Our specific responses to the points raised by the individual reviewers are shown in italics below. Newly added figures are shown in bold.

Reviewer #1 (Comments to the Author):

Martins et al. show increased expression of miR-499-5p in bipolar patients and healthy subjects with a history of childhood maltreatment. By backtranslation to rodents, they demonstrate a parallel upregulation of miR-499-5p in the hippocampus of rats after juvenile social isolation. Furthermore, miR-499-5p reduces surface expression and activity of L-type Ca channels due to targeting CACNB2.
Given the lack of insight on the pathophysiology of bipolar disorder in the absence of a suitable animal model for BD, the findings regarding miR-499-5p in bipolar humans; and the demonstration of a clear target and MOA of this miRNA by backtranslation to rodents is of major importance and near to ground-breaking. However, the manuscript could be further improved:
The relationship between BD and childhood maltreatment (CMT) is not straightforward. As the authors state, BD is one of the most "genetic" and heritable disorders. On the other hand, CMT is clearly environmental. There is a chance that BD parents have a greater risk to maltreat their offspring, e.g. by neglect during mood episodes. Moreover, CMT might impair the clinical course of BD, as stated in reference 11 or 62, 63. CMT is clearly involved in the etiology of chronic depression, but not in BD, even if it worsens the clinical course. Therefore, there is no clear causal connection between the two entities, BD and CMT. The authors should either consider to eliminate the CMT story line in this manuscript; or to use it as a substitute animal model for BD in the absence of an established animal model.

- *We agree that the relationship between BD and CMT is highly complex, and that a causal role for CMT in BD development has not been formerly demonstrated. In fact, our new data (**new Fig. EV5D**) shows that miR-499-5p is upregulated in BD patients irrespective of their history of CMT, which would further support that CMT is not the main driver of miR-499-5p expression in BD. Nevertheless, CMT clearly has an effect on miR-499-5p in healthy subjects (**new Fig. 5E**), suggesting that it might still contribute to miR-499-5p dysregulation. To account for these findings, we decided to de-emphasize the role of CMT in BD throughout the manuscript, but still keep the most important pieces of data. The following amendments were made: 1) We have re-phrased the introduction, now focusing on the fact that CMT has an adverse influence on BD disease progression and severity (Refs. 11, 62), rather than etiology (p.3). 2) We have moved some figures related to CMT in humans and juvenile social isolation in rats to the Expanded/Appendix material (**new Fig. EV1, EV5, Appendix Fig. S3**). 3) we no longer use the CMT/JSI data as a rationale to study the involvement of miR-499-5p in BD pathogenesis.*

In order to clarify if miR-499-5p is increased in BD by itself or only in BD individuals with CMT, or only in the depressed phase of BD; the authors should show miR-499-5p in subgroups of BD (<u>with/out CMT</u>, euthymia, depression, mania). For this, numbers might be increased. Or, to put it differently: is there a causal relation between Fig. 1 B and C?

- *We completely agree with this reviewer and have now further stratified the data in the revised manuscript, considering the effect of CMT **(new Fig. EV5D; Appendix Fig. S3)**, different BD subtypes (BDI, BDII; **new Fig. 5C; Appendix Fig. S3**), as well as different BD stages (depressive, euthymic, hypomanic, manic) **(new Fig. 5D;***

Appendix Fig. S3). *This analysis revealed that miR-499 is significantly upregulated in both the BD/CMT and BD/no CMT groups. Moreover, there is no significant difference in miR-499 expression between BDI/BII or dependent on the stage of the disease, although the sample size for the "manic" and "mixed" patient subgroups is insufficient for a conclusive statistical assessment. Together, our results suggest that miR-499 is rather a "trait biomarker" than a "state biomarker" in BD, and that CMT is not the main driver of miR-499 dysregulation in BD (see also our comments to reviewer 3).*

The authors show convincingly that miR-499-5p regulates CACNB2 and impair the activity of L-type Ca-channels. L-type channels are involved in synaptic plasticity (Ref 7, 8, PMID: 29174591); synaptic plasticity is an attractive candidate to explain the pathophysiology of affective disorders and the modulation of mood-related networks by environment and stress (e.g. PMID: 15738959). This could be further explored in rAAV-treated animals in Fig. 5 by direct assessment of calcium currents and hippocampal synaptic plasticity to bridge the gap between Ca-channel impairment, plasticity-related behavior (Fig. 5E), and structural changes as shown in Fig. 5F. Moreover, such experiments could help to answer questions on the direction of changes in calcium concentrations as discussed on p17.

- *We completely agree that it is important to provide cellular correlates of impaired plasticity in the rodent model. The proposed assessment of calcium currents is interesting, but technically extremely challenging, since it would require the measurement of calcium currents in acute slices. We obtained fixed slices from the rat cohort from our collaboration partner (Prof. Wöhr, Leuven), but these couldn't be used anymore for recordings. On the other hand, our collaborator is not set up to do such experiments and transferring acute slices to our laboratory was logistically impossible. Therefore, we focused on the structural aspect, and measured dendritic morphology in fixed slices from rAAV injected animals by confocal microscopy, similar to our data from miR-499 transfected primary neurons. Unfortunately, we were unable to reliably re-construct dendrites from rAAV-injected rats, probably due to a poor fixation of the slices which severely compromised neuronal morphology (data not shown). Therefore, we had to perform independent injections of rAAV-miR-499 (hp499) into the hippocampus of wild-type mice at our laboratory at ETH Zurich. Thereby, we detected a slight reduction in mean intersection number by Sholl analysis in hp499 compared to hpCTL injected mice, in particular at large distances from the neuronal cell soma (see below)[Figures for referees not shown.] . However, the difference was not statistically significant, probably due to an insufficient sample size with respect to the observed variance. We couldn't achieve higher cell numbers, since re-construction of intact dendritic trees required a technically challenging biocytin filling of individual hippocampal neurons using an electrophysiology set-up. Taken together, we cannot draw strong conclusions from this dataset at present and decided not to include it into the current manuscript.*

- *See our comments above on the measurement of dendrite structure. Animal MRT is currently not set up in our laboratories and therefore considered out of the scope.*

Reviewer #2 (Comments to the Author):

This is an interesting study on miRNA-499-5p as a regulator of the voltage-gated calcium channel CACNB2 in the context of dendritic branching and bipolar disorder (BD), especially following early childhood neglect. The authors correctly acknowledge the complexity of the processes involved, and selected one specific pathway to zoom into these processes and provide comprehensive multi-leveled information on its consequences. They identified miRNA-499-5p, which had been known before as associated with BD, as a regulator of the voltage-gated CACNB2 channel, which was identified before as a BD risk gene; and focused on the corresponding neuronal structure-function links.

Briefly, the authors experimentally demonstrated direct targeting of the neuronal Cav1.2 channel by miRNA-499-5p, showed the relevance of this interaction for electrophysiology changes and dendritic branching modifications of neurons, and used engineered rats with haplo-insufficient CACNB2 channel and impaired behavior to indicate a direct link between the studied miRNA, channel and mental consequences characteristic of BD. Last, but not least they present volume changes in the left transverse/superior temporal gyrus region of human BD patients, which is compatible with the proposed context, and demonstrate elevated levels of miRNA-499-5p in blood cells from BD patients, especially those who suffered from child neglect; which is new and interesting but of translational, rather than mechanistic level and will better belong at the end of the Results section. Notably, the impressive strength points of this study raise several queries, responding to which can improve the impact of
this manuscript substantially; as is listed below.

- *We thank the reviewer for the suggestion to move the human data at the end of the manuscript and have re-arranged the figures accordingly.*

1. miRNA-499-5p is most likely one of many miRNAs that associate with BD; short

RNA-sequencing of the studied cells and tissues can show if its differential expression level exceeds that of other BD-related miRNAs, as compared to patients' brain RNA-seq studies and Resource materials; and the biological pathways targeted by these miRNAs can also provide interesting value.

- *We agree with this reviewer that miR-499 is likely not the only miRNA involved in BD and that a more global view of miRNA expression in BD models would be informative. However, in this study, we focused on miR-499 as a candidate since it was identified as one of the three most promising candidates after correction for multiple testing in a large-scale BD GWAS (Forstner et al., 2015), and follow-up studies provided further support for an involvement of the miR-499 regulated network in BD (Reinbold et al., Front. Psych. 2018; Tielke et al., Transl. Psych., in revision). Furthermore, miR-499 differential expression level exceeded that of other disease-related microRNAs based on our qPCR analysis* ***(new Fig. 1B, Fig. EV1D, E)****, further suggesting a prominent role of miR-499. In addition, we are currently investigating other BD-associated miRNAs and their downstream biological pathways. These results, which also include RNA-seq of mouse tissue with manipulated miRNA activity, are however beyond the scope of the present study and will be part of a separate manuscript.*

2. Likewise, the neuronal Cav1.2 channel is surely one of many modified mRNAs in these systems; long RNA-seq or analysis of datasets thereof can readily point at those, and the transcriptomic analyses will put the clinical implications of this study in a more rationalized context.

- *We again agree that long RNA-seq of the rat or human BD models would have been informative. However, in the context of this project, we decided to focus on the impact on neuronal Cav1.2 activity. This decision was based on multiple reasons: 1) Cacnb2 had already been identified as a miR-499 target in the cardiac system (Ling et al., BBA Clin. 2017); 2) Cav1.2 subunits, in particular Cacna1c, have been repeatedly associated with BD and SCZ (e.g. Craddock and Sklar, Lancet 2013). 3) cellular and behavioural phenotypes of miR-499 overexpression (e.g. reduced dendritogenesis, reduced LVGCC activity, impaired short-term memory) are consistent with reduced Cav1.2 activity. Beyond the scope of this study, we are currently exploring miR-499-dependent regulation of gene expression in a more global fashion to identify additional targets and pathways potentially involved in BD. Long-RNA seq from miR-499 overexpressing primary rat cortical neurons already revealed numerous new potential miR-499 targets which can be followed-up mechanistically in the future. This has now been mentioned in the discussion of the revised manuscript.*

3. Other genes and proteins were shown to relate to dendritic branching of brain neurons; the extent of their impact compared to the currently studied voltage-gated channel should be tested for several of those, quantified and compared to the outcome of the cited articles.

- *We agree that numerous genes have been implicated in the control of dendritic branching, including ion channels, cytoskeletal regulators, metabolic genes, etc. Characterizing those in great detail would be clearly beyond the scope of the present study, which focusses on the characterization of the novel miR-499-Cacnb2-Cacna1c pathway (see also above).*

4. The relevance of the cellular processes modified in male and female BD patients should be tested separately, and studies referring to the distinct miRNAs expressed in the brain of men and women BD patients must be cited and discussed as well.

- *We agree that sex-specific effects should be considered. Therefore, we have now assessed miR-499 expression separately in male and female patients suffering from BD, MDD and SCZ* ***(new Fig. 5A-D; Appendix Fig. S3)****. Interestingly, miR-499 was significantly elevated in both male and female BD patients* ***(new Appendix Fig. S3)****, suggesting that there were no sex-dependent effects in miR-499 regulation in the context of BD. In contrast, miR-499 was not differentially expressed in either male or female MDD and SCZ patients (****new Appendix Fig. S3****), although there might be a trend for miR-499 upregulation in female MDD patients. We are also not aware of any studies which address sex-dependent regulation of microRNAs in the context of BD that could be cited and discussed.*

5. That both increases and decreases of the studied voltage-gated channel may cause mental impairments is of special interest and the potential processes involved should be discussed.

- *We agree that this dichotomy is highly interesting and have therefore devoted an entire paragraph in the discussion to it.*

6. Methods: please use 3-4, and surely more than one normalizing gene in qPCR tests.

- *We have extensive experience with the quantification of microRNAs from different sources by qPCR, and U6 snRNA in our hands is extremely stable across tissues and environmental conditions. We therefore don't see the need for performing additional qPCR reactions with different normalizing genes.*

7. Cell culture tests: were the cells of male or female origins? In either case, cells originated from the opposite sex should be studied in comparison.

- *Primary neuron cultures are of mixed sex, since they are derived from multiple rat embryos of both sexes which are combined during the preparation. Distinguishing between the sexes at the cell culture level would represent a significant work burden. First, dissections would have to be performed at the single embryo level to avoid mixing of the embryos during preparation. Second, the production of neurons would have to be significantly scaled up to achieve the same amount of material necessary for the analysis. This would not only increase the costs significantly, but also lead to an increased use of animals in conflict with the RRR criteria for animal research. Also in light of the lack of sex-dependent effects in our rat and human data, we felt that the benefit-cost ratio is too low to justify these additional experiments.*

Reviewer #3 (Comments to the Author):

The study by Martin et al. investigates miR-499-5p in the nervous system. The approach is based on GWAS data showing that miR-499-5p is associated with bipolar disease (BD). They observe increased expression of miR-499-5p in PBMC of BD patients, show that social isolation increases miR-499-5p in the rat hippocampus and subsequently perform a number of functional experiments to demonstrate that miR-499-5p controls the levels of Cacnb2 and moreover affects neuronal plasticity and short-term memory in rats. The study is very interesting and novel. The authors establish miR-499-5p as an important factor in affective disorders. At the same time, as the study stands at present, the various observations are not always well connected and some data appear preliminary. Thus, while I am very enthusiastic about the overall manuscript, some questions remain.

Major issues to be addressed:

1.
That the author's aim to link miR-499-5p expression in BD to childhood maltreatment and thereby verify the chosen animal model is understandable. However, this reviewer is not convinced that the corresponding data are convincingly presented. The authors base their decision to study juvenile social isolation as a model for depression on the observation in BD patients and especially those suffering from childhood maltreatment. However, regarding the data show in Fig 1B, C: How does the expression of miR-449-5p present when BD patients without documented history of childhood maltreatment are compared to patients that suffered from maltreatment? Is the dataset "BD" in panel B significant when compared to dataset "maltreated" in panel C. Looking at the data, this is not very likely. Thus, we can only conclude form these data that miR-499-5 expression is increased in PBMCs of BD patients irrespective of the exposure to childhood maltreatment.

- *As suggested by this reviewer and reviewer 1, we have now analysed miR-499 expression separately in BD patients with or without a history of childhood maltreatment **(new Fig. EV5D)**. Thereby, we did not observe an effect of CMT, suggesting that miR-499 is increased in PBMCs of BD patients irrespective of the exposure to childhood maltreatment, in agreement with the assumption of this reviewer.*

2. Along the same line, this reviewer wonders if the data shown in supplemental figure 1 is truly relevant as presented. It should be tested if the expression levels of miR-499-5p are similarly correlated with other phenotypic data such as BDI etc.

- *According to the suggestion of this reviewer, we have now also tested a potential correlation between miR-499 expression levels and the Beck Depression Inventory (BDI; a measure for the severity of depression) and the Young Mania rating scale (YMRS; a measure of manic symptoms) in BD patients **(new Fig. EV5E, F)**. Interestingly, we did not find a significant correlation for both BDI and YMRS with miR-499 expression levels in PBMCs, further supporting the idea that miR-499 is not a state-dependent biomarker in BD.*

3. Please provide the information in which phase of the disease, blood was collected from BD patients.

- *We have now included this information in **Appendix Table T2**. In addition, we have provided miR-499 expression levels for the different subgroups of BD patients **(new Fig. 5D; Appendix Fig. S3)**.*

4. Please provide information what type of mediation was taken by the patients described in supplementary table 1 and indicate how many patients received medication for the data shown in supplemental table 2.

- *We have now included this information in the supplementary table. In addition, we have now provided miR-499 expression levels for BD/MDD patients with and without antidepressant (AD) treatment **(new Fig. EV5B, C)**. Based on this analysis, AD treatment does not appear to affect miR-499 expression in BD/MDD patients. However, we note that the number of MDD patients not receiving medication is rather low (in particular for females), making it difficult to draw strong conclusions.*

5. The fact that childhood maltreatment does not seem to drive miR-499-5p expression in BD patients within the analyzed dataset, does not necessarily suggest that childhood maltreatment is not relevant for aberrant miR-499-5p expression. In fact, the rat model

supports such a role and to some extend also the DEX treatment. As a reader, I would still wonder if other environmental risk factors would affect miR-499-5p expression. There are other animal models to induced depressive-like phenotypes and even pharmacological models of BD that aim to mimic manic symptoms. It would be very interesting to see if such environmental factors also affect miR-499-5p levels. This would also help to better understand if miR-499-5p expression is specifically linked to BD or is more generally affected for example in patients suffering from major depression or other neuropsychiatric disease that share a neurodevelopmental component.

- *We agree that the scope of miR-499 regulation by environmental risk factors and in the context of other neuropsychiatric diseases could be expanded. Unfortunately, other genetic or pharmacological animal models of BD were not established in our laboratory at the time of revision. The set-up of such models is clearly beyond the scope of this project, given the extremely long time periods required to establish new mouse/rat cohorts and to obtain the respective governmental approvals to perform animal experiments. However, we were fortunate to obtain human PBMC material from patients suffering from MDD and SCZ through our collaboration with the FOR2107 consortium. Analysis of miR-499 levels by qPCR in these samples revealed that miR-499 was not differentially expressed between MDD/SCZ and control subjects* ***(new Fig. 5A, B; Fig. EV5A-C)****. Together, these results suggest a rather specific upregulation of miR-499 in PBMCs of BD subjects.*

6. It would be important to test if emotional neglect in the employed rat model also leads to increased miR-499-5p expression in PBMCs.

- *we agree that this would represent an interesting piece of data, but unfortunately, PBMC isolation from rats is currently not established in the laboratory and we do not have an animal license to perform such experiments. Moreover, the newly generated 2nd cohort alone would not allow to obtain conclusive data due to the limited number of additional animals (see also our statement below).*

7. The human data stems exclusively from female individuals. The rat data shown in Fig 1 was generated however exclusively from male rats while the data show in Fig. 2A is exclusively based on brains from female rats. Finally, the novel object recognition test is performed in “male and female WT and Canca1c+/- rats” with no further information on the sex distribution. One wonders why? Please address.

- *We thank the reviewer for pointing this out and have now attempted to achieve an equal distribution of both sexes for the rat and human datasets. For the rat behavioural data (****new Fig. 4G, H; Fig. EV4C-E****), we have indicated the sex of each of the animals with different symbols. Even though we substantially increased the number of animals in our study by adding a second cohort of 33 animals, the number of animals per condition is still too low when split by sex. For example, in Cacna1c+/-_Control group even though we have an N of 15 animals, we only have 5 males and 10 females, which makes statistical comparisons unreliable.*
  Furthermore, in our previous study on object recognition memory in male and female Cacna1c rats we did not obtain evidence for sex effects despite a substantially high number of subjects, with 20 animals per sex and genotype combination, that is in total 80 animals. (Braun et al, Neurobiol Learn Mem, 2018).
  For the human data, we have now in addition measured 92 male BD, MDD and healthy control patients ***(new Appendix Fig. S3)****. Statistical assessment of this data didn’t reveal any major sex-dependent effects on miR-499 expression in PBMCs.*

8. Fig.2 Please demonstrate that administration of miR-499-5p mimics leads to increased levels of this construct in the treated cells.

- *Following the suggestion of this reviewer, we have now measured miR-499 levels by qPCR in neurons transfected with miR-499 mimics and observed a highly significant upregulation compared to control-transfected neurons* ***(new Fig. EV1H)*** *by qPCR.*

9. Panel 2G is mentioned in the main text but seems to be missing from Fig. 2.

- *We apologize for this mistake and now provided the missing panel (schematic of the miR-499 binding site in the Cacnb2 3'UTR) in the revised manuscript* ***(new Fig. 2A)****.*

10. Fig 3: Please show that Cacnb2 protein levels change in response to miR-499-5p mimic.

- *We have already provided data which shows Cacnb2 protein downregulation upon miR-499 expression in vivo* ***(new Fig. 4D-F)****. This has now been complemented with Western blot data for miR-499 mimic transfection in primary rat hippocampal neurons* ***(new Fig. EV2A, B)****.*

11. In the context of Fig.3 the authors refer to Supplementary Figure 5 which is supposed to show the pR2-CACNB2 construct. I don't think this is what's shown in the corresponding figure. Please clarify. I guess the Cacnb2 overexpression construct does not contain the 3' UTR with the miR-499 binding site?

- *We apologize for the confusion, the respective figure showing the Cacnb2 expression construct was in fact not shown in the previous version but has now been included in the revised version* ***(new Fig. EV2C)****. And yes, the construct doesn't include a 3'UTR to avoid targeting by miR-499 in the rescue experiment.*

12. Fig.4A. The authors state "In accordance with previous work showing a post-synaptic localization of Cav1.2 at dendritic shafts and cell bodies (60), we detected Cav1.2-HA puncta in both compartments (Figure 4A)". Please provide corresponding co-staining to claim post-synaptic localization or reword.

- *in fact, we didn't intend to make claims about (post)synaptic localization of Cav1.2-HA, but rather dendritic vs. somatic localization which can be inferred without co-stainings (please bear in mind that the HA-construct is co-transfected with GFP* ***(new Fig. 3A)****, which rules out the possibility that the observed puncta are localized presynaptically in non-transfected neurons contacting these cells. We have adjusted the wording in the revised version accordingly.*

13. Fig. 4B. The significant difference shown in the quantification in is difficult to appreciate in panel A. Please provide high magnification images in addition.

- *We agree and have now provided higher magnification images as insets in the corresponding figure* ***(new Fig. 3A)****.*

14. Fig. 4 D, E. Please show that these phenotypes are rescued by overexpression of Cacnb2 as shown in Fig. 3 F-G.

- *As suggested by this reviewer, we have now attempted to rescue miR-499-dependent reduction of L-type channel-mediated calcium currents in cultured hippocampal neurons by co-expression of Cacnb2* ***(new Fig. 3E, F)****. Thereby, we observed a strong upregulation of calcium currents upon Cacnb2 overexpression*

irrespective of miR-499 mimic co-transfection. Thus, Cacnb2 expression not only rescues the observed phenotype, but leads to a gain-of-function, probably since Cacnb2 expression levels exceed those observed under physiological conditions in hippocampal neurons. This in turn further promotes Cav1.2 membrane localization and/or stimulates channel conductance.

15. Fig 5E. To better appreciate the fact that miR-449-5p mimics only produce a phenotype in Cancna1c+/- mutant mice, it would be important to assays Cacnb2 protein levels in these groups as shown in panel E. In turn, the analysis of Cancna1c protein levels in response to miR-499-5p mimics (as shown in panel E) would help to appreciate better the impact of miR-499-5p on the function of Cav1.2.

- ➢ *As suggested by this reviewer, we have now performed in addition Cacnb2 Western blot in lysates obtained from Cacna1c+/- brains* ***(new Fig. 4D-F)****. As expected, we observed a similar decrease in Cacnb2 expression induced by miR*-499 in WT and Cacna1c +/- *brains, consistent with miR-499/Cacnb2 being upstream of Cacna1c. This data suggests that, in order to produce a memory phenotype in rats, reduced Cacnb2 expression induced by miR-499 has to interact with reduced levels of the Cav1.2 pore-forming subunit Cacna1c. If both are reduced below a certain threshold, Cav1.2 surface expression might become too low to support plasticity processes involved in memory. This has now been discussed in the revised manuscript.*

16. Fig 5F. Please provide the corresponding correlations of miR-499 expression with imaging parameters as scatter plots within Fig. 5.

- ➢ *We have now provided a modified version of this Fig. as scatter plot* ***(new Fig. 5G)****.*

17. This reviewer really appreciates that the authors performed behavior studies to elucidate the role of miR-499-5p in BD. At the same time, the presented findings raise a number of questions that should be addressed. (1) The study seems to be underpowered. Why would the WT–AAV-Control group not learn the test? This experimental group is even the largest with n=12. (2) Especially the key findings in Cacna1c+/-_AAV-miR-499 mice are based on n=8, which – taken together- does not give much confidence in the data at present.
In addition, defects in short term memory does not come to mind as a most specific BD-like phenotype that can be measured in rodents. It would be helpful to measure for example social interaction and/or depressive-like phenotypes.

- ➢ *We agree that the previous dataset might have been slightly underpowered, which mainly was a result of technical difficulties encountered during stereotactic injections of rats. Consequently, a number of animals had to be excluded from the analysis due to insufficient expression of the transgene in the hippocampus* ***(new Appendix Fig. S1 and S2)****. To address this, we have now optimized our injection protocol and performed injections of a 2nd rat cohort to increase overall sample size. The aggregated results of these cohorts (total n=16-19) are now shown in* ***new Fig. 4G, H and Fig. EV4C-E****. The main conclusions from the data remain unchanged, namely that novel object recognition (NOR) memory is selectively abolished upon miR-499 expression in Cacna1c+/-, but not WT rats (see also above). Importantly, we now observe a statistically significant difference between observation times of the novel vs. familiar object in WT control rats, suggesting that this group effectively learned the test.*
- ➢ *Concerning BD-specificity, we agree that defects in short-term memory (as assessed e.g. by NOR) are not the most specific phenotype associated with BD. Nevertheless, as mentioned in the introduction, cognitive deficits are frequently observed in BD patients. Moreover, we were guided by our observation of miR-499-dependent*

downregulation of Cav1.2 channel activity, the latter of which has been frequently associated with memory- and anxiety-related processes *<u>in the hippocampus</u>*. *For these reasons, we focused on the characterization of memory and anxiety-like behaviour for this initial characterization of miR-499 function* ***(new Fig. 4G, H and Fig. EV4C-E)****.*

- *In the meantime, we have already started the analysis of social and depressive-like phenotypes in our model. For example, forced swim test (FST) was performed to assess behavioural despair (see below)[Figures for referees not shown.]. No effects of genotype or miR-499 expression were observed in this paradigm. However, these preliminary results do not allow to draw conclusions about the role of miR-499 in emotional processing in general, since these behaviours might be affected by brain regions outside the hippocampus in which miR-499-5p levels were unaltered. We therefore decided to omit this data from the current manuscript and rather show it in the context of a more comprehensive characterization of BD-associated behaviours in a separate future manuscript.*

18. Does administration of mir-499-5p to the hippocampus affect motorfunction or other behaviors that could affect performance in the NOR test?

- *We have now assessed motor function (i.e. total distance travelled) in the open field test* ***(new Fig. EV4E)*** *and did not find any miR-499- or Cacna1c-dependent effects in total distance travelled during a 10-min time period on two consecutive days. In addition, we did not find any differences in total arm entries in the EPM upon miR-499 administration* ***(new Fig. EV4D)****. We are therefore confident that our results from NOR are not confounded by genotype- and/or miR-499-dependent effects on motor*

*function of rats. Moreover, miR-499 administration also did not significantly change anxiety levels **(new Fig. 4H)**, which could also affect performance in the NOR test.*

19. In the discussion the authors address the potential reason for the elevated miR-499-5p levels in the blood and cite data to support a hypothesis that "chronic stress might lead to aberrant miRNA-499-5p expression in the heart and secretion of miR-499-5p into the bloodstream inside exosomes that then reach the brain". Why is miR-499-5p then increased in PBMCs as measured in the present study?

- *We agree with this reviewer that upregulation of miR-499 in PBMCs (and potentially neurons) might happen by an alternative mechanism, e.g. stress-mediated epigenetic modifications at the miR-499 gene locus. We have rephrased this part of the discussion accordingly.*

Minor issues:
1. Please show violin plot rather than box plots to appreciate the distribution of the data (Figs 1B, C.)

- *We thank the reviewer for this suggestion and have now created violin plots for all the human data **(new Fig. 5A-E; Fig. EV5B-D; Appendix Fig. S3)**.*

2. When discussing the rat models the authors sometimes refer to early life stress, physical or emotional neglect. Please clarify and use consistent terminology.

- *We have now attempted to use consistent terminology throughout the text in the revised manuscript.*

3. Fig 2B: It seems that miR-499-5p is also present in neurites and maybe synapses. I suggest analyze this in more detail via co-staining with corresponding marker proteins for the pre and postsynapse. Is miR-499-5p a synaptic microRNA?

- *In fact, our previous data does not provide strong support for a synaptic localization of miR-499 and its target Cacnb2 mRNA. For example, we did not detect an enrichment of miR-499 in synaptoneurosomes, compared to other synaptic miRNAs (e.g. miR-138), in the rat forebrain (Siegel et al., NCB 2009). Also, the Cacnb2 mRNA was not strongly expressed in dendrites based on our RNA-seq data of compartmentalized neurons (Colameo et al., EMBO Rep. 2021). Therefore, we did not see a strong rationale for studying synaptic localization of miR-499 in further detail.*

4. Supplementary Fig. 2B. Please plot control and DEX groups separately

- *We have now plotted these groups separately in the revised manuscript **(new Fig. EV1G)**.*

5. Does social isolation increases miR-499-5p levels specifically in neurons or also other cell types?

- *Addressing this question would require FISH experiments in rat hippocampal slices, which are currently not set up in the laboratory. Especially given the fact that miR-499 is expressed in the brain at rather low levels compared to other microRNAs, establishing this assay would likely require extensive efforts which are beyond the scope of the current study.*

Dear Gerhard,

Thank you for the submission of your revised manuscript. We have now received the enclosed report from referee 1 who has also reviewed the first version of your manuscript and who assessed your response to all referee concerns. Referee 1 is satisfied with the revised study and we can therefore in principle accept it. Only a few minor editorial requests will still need to be addressed.

- Your manuscript has 5 main figures but is layed out as a full article. Please either add one more main figure or combine the results and discussion sections and reduce the total word count to 27.000 characters (excluding materials and methods and references) to publish it as a short report.

- Please reduce the number of keywords to 5.

- Please correct the subheading of the conflict of interest statement to "Disclosure and Competing Interest Statement"

- Please add the contributions of Susanne Meinert and Udo Dannlowski to the author credits.

- REFERENCE FORMAT: The year is not always bracketed. Please correct.

- Please add callouts for all Appendix figure panels.

- Appendix table callouts need correcting to 'Appendix Table S#'.
Appendix table names need correcting to 'Appendix Table S#'.

- Appendix Fig S1 - scale bars missing, please add.

I attach to this email a related manuscript file with comments by our data editors. Please address all comments in the final manuscript.

EMBO press papers are accompanied online by A) a short (1-2 sentences) summary of the findings and their significance, B) 2-3 bullet points highlighting key results and C) a synopsis image that is exactly 550 pixels wide and 200-600 pixels high (the height is variable). You can either show a model or key data in the synopsis image. Please note that text needs to be readable at the final size. Please send us this information along with the revised manuscript.

I look forward to seeing a final version of your manuscript as soon as possible. Please use this link to submit your revision: https://embor.msubmit.net/cgi-bin/main.plex

Best wishes,
Esther

Esther Schnapp, PhD
Senior Editor
EMBO reports

Referee #1:

The authors have more than adequately responded to the points of the reviewers, they have provided new experimental series and figures and revised the wording so that the manuscript has gained a lot.

The authors have addressed all minor editorial requests.

Prof. Gerhard Schratt
ETH Zurich - D-HEST
Systems Neuroscience, Bldg Y17 L48
Institute for Neuroscience
Winterthurerstr. 190
Zurich, Zurich 8057
Switzerland

Dear Gerhard,

I am very pleased to accept your manuscript for publication in the next available issue of EMBO reports. Thank you for your contribution to our journal.

At the end of this email I include important information about how to proceed. Please ensure that you take the time to read the information and complete and return the necessary forms to allow us to publish your manuscript as quickly as possible.

As part of the EMBO publication's Transparent Editorial Process, EMBO reports publishes online a Review Process File to accompany accepted manuscripts. As you are aware, this File will be published in conjunction with your paper and will include the referee reports, your point-by-point response and all pertinent correspondence relating to the manuscript.

If you do NOT want this File to be published, please inform the editorial office within 2 days, if you have not done so already, otherwise the File will be published by default [contact: emboreports@embo.org]. If you do opt out, the Review Process File link will point to the following statement: "No Review Process File is available with this article, as the authors have chosen not to make the review process public in this case." Please note that the author checklist will still be published even if you opt out of the transparent process.

Thank you again for your contribution to EMBO reports and congratulations on a successful publication. Please consider us again in the future for your most exciting work.

Best regards,
Esther

Esther Schnapp, PhD
Senior Editor
EMBO reports

****

THINGS TO DO NOW:

Please note that you will be contacted by Wiley Author Services to complete licensing and payment information. The required 'Page Charges Authorization Form' is available here: https://www.embopress.org/pb-assets/embo-site/er_apc.pdf - please download and complete the form and return to embopressproduction@wiley.com

You will receive proofs by e-mail approximately 2-3 weeks after all relevant files have been sent to our Production Office; you should return your corrections within 2 days of receiving the proofs.

Please inform us if there is likely to be any difficulty in reaching you at the above address at that time. Failure to meet our deadlines may result in a delay of publication, or publication without your corrections.

All further communications concerning your paper should quote reference number EMBOR-2021-54420V3 and be addressed to emboreports@wiley.com.

Should you be planning a Press Release on your article, please get in contact with emboreports@wiley.com as early as possible, in order to coordinate publication and release dates.

EMBO Press Author Checklist

Corresponding Author Name: Gerhard Schratt	
Journal Submitted to: EMBO Reports	
Manuscript Number: EMBOR-2021-54420V1	

USEFUL LINKS FOR COMPLETING THIS FORM
The EMBO Journal - Author Guidelines
EMBO Reports - Author Guidelines
Molecular Systems Biology - Author Guidelines
EMBO Molecular Medicine - Author Guidelines

Reporting Checklist for Life Science Articles (updated January 2022)
This checklist is adapted from Materials Design Analysis Reporting (MDAR) Checklist for Authors. MDAR establishes a minimum set of requirements in transparent reporting in the life sciences (see Statement of Task: 10.31222/osf.io/9sm4x). Please follow the journal's guidelines in preparing your manuscript.
Please note that a copy of this checklist will be published alongside your article.

Abridged guidelines for figures
1. Data
The data shown in figures should satisfy the following conditions:
- the data were obtained and processed according to the field's best practice and are presented to reflect the results of the experiments in an accurate and unbiased manner.
- ideally, figure panels should include only measurements that are directly comparable to each other and obtained with the same assay.
- plots include clearly labeled error bars for independent experiments and sample sizes. Unless justified, error bars should not be shown for technical replicates.
- if n<5, the individual data points from each experiment should be plotted. Any statistical test employed should be justified.
- Source Data should be included to report the data underlying figures according to the guidelines set out in the authorship guidelines on Data Presentation.

2. Captions
Each figure caption should contain the following information, for each panel where they are relevant:
- a specification of the experimental system investigated (eg cell line, species name).
- the assay(s) and method(s) used to carry out the reported observations and measurements.
- an explicit mention of the biological and chemical entity(ies) that are being measured.
- an explicit mention of the biological and chemical entity(ies) that are altered/varied/perturbed in a controlled manner.
- the exact sample size (n) for each experimental group/condition, given as a number, not a range;
- a description of the sample collection allowing the reader to understand whether the samples represent technical or biological replicates (including how many animals, litters, cultures, etc.).
- a statement of how many times the experiment shown was independently replicated in the laboratory.
- definitions of statistical methods and measures:
  - common tests, such as t-test (please specify whether paired vs. unpaired), simple χ2 tests, Wilcoxon and Mann-Whitney tests, can be unambiguously identified by name only, but more complex techniques should be described in the methods section;
  - are tests one-sided or two-sided?
  - are there adjustments for multiple comparisons?
  - exact statistical test results, e.g., P values = x but not P values < x;
  - definition of 'center values' as median or average;
  - definition of error bars as s.d. or s.e.m.

Please complete ALL of the questions below.
Select "Not Applicable" only when the requested information is not relevant for your study.

Materials

Newly Created Materials	Information included in the manuscript?	In which section is the information available? (Reagents and Tools Table, Materials and Methods, Figures, Data Availability Section)
New materials and reagents need to be available; do any restrictions apply?	Yes	Materials and Methods

Antibodies	Information included in the manuscript?	In which section is the information available? (Reagents and Tools Table, Materials and Methods, Figures, Data Availability Section)
For **antibodies** provide the following information:<br>- Commercial antibodies: RRID (if possible) or supplier name, catalogue number and or/clone number<br>- Non-commercial: RRID or citation	Yes	Materials and Methods

DNA and RNA sequences	Information included in the manuscript?	In which section is the information available? (Reagents and Tools Table, Materials and Methods, Figures, Data Availability Section)
**Short novel DNA or RNA including primers, probes**: provide the sequences.	Yes	Materials and Methods

Cell materials	Information included in the manuscript?	In which section is the information available? (Reagents and Tools Table, Materials and Methods, Figures, Data Availability Section)
**Cell lines:** Provide species information, strain. Provide accession number in repository **OR** supplier name, catalog number, clone number, and/**OR** RRID.	Not Applicable	
**Primary cultures:** Provide species, strain, sex of origin, genetic modification status.	Yes	Materials and Methods
Report if the cell lines were recently **authenticated** (e.g., by STR profiling) and tested for mycoplasma contamination.	Not Applicable	

Experimental animals	Information included in the manuscript?	In which section is the information available? (Reagents and Tools Table, Materials and Methods, Figures, Data Availability Section)
**Laboratory animals or Model organisms:** Provide species, strain, sex, age, genetic modification status. Provide accession number in repository **OR** supplier name, catalog number, clone number, **OR** RRID.	Yes	Materials and Methods
**Animal observed in or captured from the field:** Provide species, sex, and age where possible.	Not Applicable	
Please detail **housing and husbandry conditions**.	Yes	Materials and Methods

Plants and microbes	Information included in the manuscript?	In which section is the information available? (Reagents and Tools Table, Materials and Methods, Figures, Data Availability Section)
**Plants:** provide species and strain, ecotype and cultivar where relevant, unique accession number if available, and source (including location for collected wild specimens).	Not Applicable	
**Microbes:** provide species and strain, unique accession number if available, and source.	Not Applicable	

Human research participants	Information included in the manuscript?	In which section is the information available? (Reagents and Tools Table, Materials and Methods, Figures, Data Availability Section)
If collected and within the bounds of privacy constraints report on age, sex and gender or ethnicity for all study participants.	Yes	Supplementary Table 1

Core facilities	Information included in the manuscript?	In which section is the information available? (Reagents and Tools Table, Materials and Methods, Figures, Data Availability Section)
If your work benefited from core facilities, was their service mentioned in the acknowledgments section?	Yes	Materials and Methods

Design

Study protocol	Information included in the manuscript?	In which section is the information available? (Reagents and Tools Table, Materials and Methods, Figures, Data Availability Section)
If study protocol has been **pre-registered, provide DOI in the manuscript**. For clinical trials, provide the trial registration number **OR** cite DOI.	Not Applicable	
Report the **clinical trial registration number** (at ClinicalTrials.gov or equivalent), where applicable.	Not Applicable	

Laboratory protocol	Information included in the manuscript?	In which section is the information available? (Reagents and Tools Table, Materials and Methods, Figures, Data Availability Section)
Provide DOI OR other citation details if **external detailed step-by-step protocols** are available.	Not Applicable	

Experimental study design and statistics	Information included in the manuscript?	In which section is the information available? (Reagents and Tools Table, Materials and Methods, Figures, Data Availability Section)
Include a statement about **sample size** estimate even if no statistical methods were used.	Yes	Materials and Methods
Were any steps taken to minimize the effects of subjective bias when allocating animals/samples to treatment (e.g. **randomization procedure**)? If yes, have they been described?	Yes	Materials and Methods
Include a statement about **blinding** even if no blinding was done.	Yes	Materials and Methods
Describe **inclusion/exclusion criteria** if samples or animals were excluded from the analysis. Were the criteria pre-established?<br><br>If sample or data points were omitted from analysis, report if this was due to attrition or intentional exclusion and provide justification.	Yes	Materials and Methods, Appendix Fig. S1, 2
For every figure, are **statistical tests** justified as appropriate? Do the data meet the assumptions of the tests (e.g., normal distribution)? Describe any methods used to assess it. Is there an estimate of variation within each group of data? Is the variance similar between the groups that are being statistically compared?	Yes	Materials and Methods, Section "Statistical Analysis"

Sample definition and in-laboratory replication	Information included in the manuscript?	In which section is the information available? (Reagents and Tools Table, Materials and Methods, Figures, Data Availability Section)
In the figure legends: state number of times the experiment was **replicated** in laboratory.	Yes	Figure legends
In the figure legends: define whether data describe **technical or biological replicates**.	Yes	Figure legends

Ethics

Ethics	Information included in the manuscript?	In which section is the information available? (Reagents and Tools Table, Materials and Methods, Figures, Data Availability Section)
Studies involving **human participants**: State details of **authority granting ethics approval** (IRB or equivalent committee(s), provide reference number for approval.	Yes	Materials and Methods
Studies involving **human participants**: Include a statement confirming that **informed consent** was obtained from all subjects and that the experiments conformed to the principles set out in the WMA Declaration of Helsinki and the Department of Health and Human Services Belmont Report.	Yes	Materials and Methods
Studies involving **human participants:** For publication of **patient photos**, include a statement confirming that consent to publish was obtained.	Not Applicable	
Studies involving experimental **animals**: State details of **authority granting ethics approval** (IRB or equivalent committee(s), provide reference number for approval. Include a statement of compliance with ethical regulations.	Yes	Materials and Methods
Studies involving **specimen and field samples:** State if relevant **permits** obtained, provide details of authority approving study; if none were required, explain why.	Not Applicable	

Dual Use Research of Concern (DURC)	Information included in the manuscript?	In which section is the information available? (Reagents and Tools Table, Materials and Methods, Figures, Data Availability Section)
Could your study fall under dual use research restrictions? Please check biosecurity documents and list of **select agents and toxins** (CDC): https://www.selectagents.gov/sat/list.htm	Not Applicable	
If you used a select agent, is the security level of the lab appropriate and reported in the manuscript?	Not Applicable	
If a study is subject to dual use research of concern regulations, is the name of the **authority granting approval and reference number** for the regulatory approval provided in the manuscript?	Not Applicable	

Reporting

The MDAR framework recommends adoption of discipline-specific guidelines, established and endorsed through community initiatives. Journals have their own policy about requiring specific guidelines and recommendations to complement MDAR.

Adherence to community standards	Information included in the manuscript?	In which section is the information available? (Reagents and Tools Table, Materials and Methods, Figures, Data Availability Section)
State if relevant guidelines or checklists (e.g., **ICMJE, MIBBI, ARRIVE, PRISMA**) have been followed or provided.	Not Applicable	
For **tumor marker prognostic studies,** we recommend that you follow the **REMARK** reporting guidelines (see link list at top right). See author guidelines, under 'Reporting Guidelines'. Please confirm you have followed these guidelines.	Not Applicable	
For **phase II and III randomized controlled trials**, please refer to the **CONSORT** flow diagram (see link list at top right) and submit the CONSORT checklist (see link list at top right) with your submission. See author guidelines, under 'Reporting Guidelines'. Please confirm you have submitted this list.	Not Applicable	

Data Availability

Data availability	Information included in the manuscript?	In which section is the information available? (Reagents and Tools Table, Materials and Methods, Figures, Data Availability Section)
Have **primary datasets** been deposited according to the journal's guidelines (see 'Data Deposition' section) and the respective accession numbers provided in the Data Availability Section?	Not Applicable	
Were **human clinical and genomic datasets** deposited in a public access-controlled repository in accordance to ethical obligations to the patients and to the applicable consent agreement?	Not Applicable	
Are **computational models** that are central and integral to a study available without restrictions in a machine-readable form? Were the relevant accession numbers or links provided?	Not Applicable	
If publicly available data were reused, provide the respective **data citations in the reference list**.	Not Applicable	
